# Systematic Studies on Anti-Cancer Evaluation of Stilbene and Dibenzo[*b,f*]oxepine Derivatives

**DOI:** 10.3390/molecules28083558

**Published:** 2023-04-18

**Authors:** Filip Borys, Piotr Tobiasz, Marcin Poterała, Hanna Fabczak, Hanna Krawczyk, Ewa Joachimiak

**Affiliations:** 1 Department of Organic Chemistry, Faculty of Chemistry, Warsaw University of Technology, 00-664 Warsaw, Poland; 2 Nencki Institute of Experimental Biology, Polish Academy of Sciences, 02-093 Warsaw, Poland

**Keywords:** dibenzo[*b,f*]oxepine, biological activity, cancer cell, microtubule, tubulin, colchicine-binding site

## Abstract

Cancer is one of the most common causes of human death worldwide; thus, numerous therapies, including chemotherapy, have been and are being continuously developed. In cancer cells, an aberrant mitotic spindle—a microtubule-based structure necessary for the equal splitting of genetic material between daughter cells—leads to genetic instability, one of the hallmarks of cancer. Thus, the building block of microtubules, tubulin, which is a heterodimer formed from α- and β-tubulin proteins, is a useful target in anti-cancer research. The surface of tubulin forms several pockets, i.e., sites that can bind factors that affect microtubules’ stability. Colchicine pockets accommodate agents that induce microtubule depolymerization and, in contrast to factors that bind to other tubulin pockets, overcome multi-drug resistance. Therefore, colchicine-pocket-binding agents are of interest as anti-cancer drugs. Among the various colchicine-site-binding compounds, stilbenoids and their derivatives have been extensively studied. Herein, we report systematic studies on the antiproliferative activity of selected stilbenes and oxepine derivatives against two cancer cell lines—HCT116 and MCF-7—and two normal cell lines—HEK293 and HDF-A. The results of molecular modeling, antiproliferative activity, and immunofluorescence analyses revealed that compounds **1a**, **1c**, **1d**, **1i**, **2i**, **2j**, and **3h** were the most cytotoxic and acted by interacting with tubulin heterodimers, leading to the disruption of the microtubular cytoskeleton.

## 1. Introduction

Cancer, the leading cause of human death worldwide, remains difficult to treat, despite a huge effort to develop novel therapies [1,2,3,4,5,6]. Chemotherapy, a traditional and commonly used anti-cancer treatment, targets specific proteins and, as a consequence, cellular structures or processes [7]. Among the most common targets of chemotherapy are proteins involved in cell cycle progression [8]. In cancer cells, the mitotic spindle—a structure that is indispensable for cell division—frequently shows abnormalities, as it causes the improper division of genetic material between daughter cells and, as a consequence, genomic instability [8]. The scaffold of the mitotic spindle is formed by microtubules (MTs), which are cylindrical, hollow biopolymers that are continuously polymerized and depolymerized. MTs are composed of tubulin heterodimers consisting of two ~55 kDa proteins, α- and β-tubulin. In interphase cells, MTs form a network involved in different cellular processes, including the establishment and maintenance of cell shape, intracellular transport, organelle distribution, and cell motility [9]. Thus, in both interphase and dividing cells, tubulin is among the most useful targets in chemotherapy [7].

The surface of tubulin contains so-called “pockets” or “sites” that can accommodate small molecules, which are called microtubule-targeting agents (MTAs). Intercalation of MTAs with the pockets affects MTs’ polymerization/depolymerization, leading to changes in the stability of MTs [9,10]. Of the six agent-binding pockets that have been identified, four bind microtubule-destabilizing agents (MDAs): vinca, colchicine, maytansine, and pironetin sites [9,10]. The binding of an MDA to tubulin causes MT depolymerization or the inhibition of its polymerization, thus affecting microtubule-dependent processes and, as a consequence, leading to cell death. The constant search for new and more specific agents targeting MDA-accommodating pockets has driven many studies [11,12,13,14,15,16]. As a result, several new compounds have been approved for cancer therapy [17,18].

**Figure 1:**
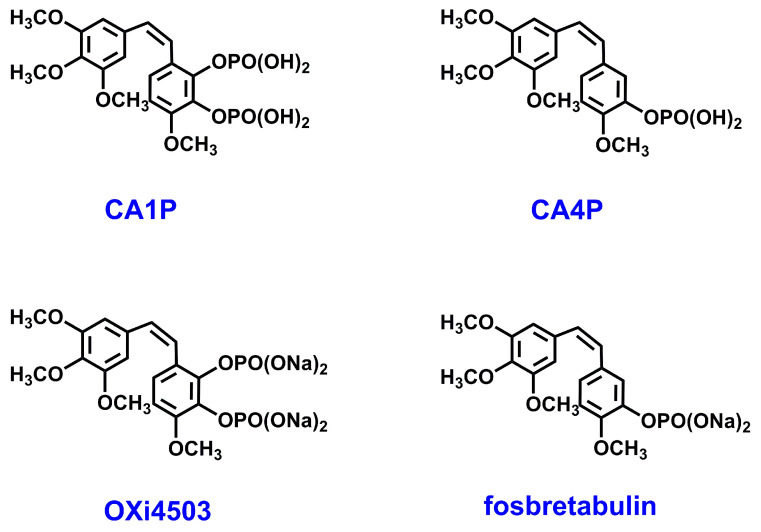
Structure of natural stilbenoids with anti-cancer activity: combretastatin CA1 and CA4 phosphate (**CA1P** and **CA4P**, respectively), as well as their disodium salts (**OXi4503** and **fosbretabulin**, respectively).

Colchicine pockets, which are the largest MDA-accommodating sites in tubulin [9,10], bind structurally diverse molecules, which usually contain one phenyl ring and another aromatic ring—phenyl, indole, thiophene, or imidazole, which are separated by a linker [18]. Colchicine-site-binding agents (CSBAs) have drawn attention because, in contrast to other MTAs, they overcome multidrug resistance [19]. Additionally, it was shown that CSBAs may target not only the microtubular cytoskeletons of cancer cells, but also those of endothelial cells that line the tumor vasculature (so-called vascular disrupting agents—VDAs); thus, by disrupting existing vessels, they can reduce the feeding of a tumor and lead to its necrosis [19,20]. Of the different types of double-target CSBAs, a large group, that of natural stilbenoids and their derivatives, such as the combretastatin family (**CA1P**, **CA4P**, **OXI4503** (Figure 1) [21,22]), have been studied as potential anti-cancer drugs [23,24]. In fact, currently, a methoxy stilbenoid, combretastatin A-4 phosphate **CA4P**/**fosbretabulin** (Figure 1), is the only CSBA that is currently approved by FDA for cancer treatment as a VDA [18,21].

On the other hand, CSBAs that include stilbenoids are poorly soluble in water, and they cause strong side effects [19]. For example, **CA4P**/**fosbretabulin**, which is generally well tolerated, causes acute but transient hypertension [25]. Therefore, efforts are being made to develop new stilbenoid derivatives in the search for more potent anti-cancer agents with improved features [26,27,28,29,30,31].

It was shown that the addition of functional groups, e.g., methyl, prenyl, O-allyl, or methoxy groups, can enhance the biological activity of natural stilbenoids [32,33,34,35,36,37,38].

In our previous study [39,40], we synthesized and evaluated the cytotoxicity of five stilbenes (**1a**–**1e**) and six dibenzo[*b*,*f*]oxepines (**2a**–**2e**, **2h**) (the latter are frequently used as scaffolds in medicinal chemistry, they have a (*Z*)-stilbene motif in their skeleton, and their aromatic rings are connected by oxygen). We showed that one substituted dibenzo[*b*,*f*]oxepine and two stilbene derivatives, while operating through tubulin binding, acted more selectively toward cancer cell lines (HeLa and U87) than toward normal cell lines. In search of novel compounds with higher cytotoxicity and selectivity toward cancer cells, we studied previously obtained compounds and synthesized new derivatives of hydroxy, methoxy, nitro, and amine groups with 4 stilbenes (**1f**–**1i**) and 13 dibenzo[*b*,*f*]oxepines (**2f**, **2g**, **2i**, **2j**, **3a**–**3j**). For the new compounds, we determined their structure by using NMR and theoretical calculations. For all 28 compounds, we evaluated cytotoxicity for the HCT116 and MCF-7 cell lines. We collected these compounds to conduct a systematic study of stilbenes with methoxy and nitro groups and of dibenzo[*b*,*f*]oxepines with nitro, methoxy, and amino groups. Our results indicated that the addition of a methoxy group could maintain or abolish the cytotoxicity of stilbene derivatives in a position-dependent manner, while, with some exceptions, the restriction of the double bonds that connected phenyl rings to create a (*Z*)-stilbene motif with an oxygen bridge decreased the compounds’ activity. Moreover, we demonstrated that three of the most active agents were selective toward cancer cell lines and that they targeted tubulin, thus disrupting the microtubular cytoskeleton.

## 2. Results and Discussion

### 2.1. Chemistry and NMR Spectra

**Scheme 1:**
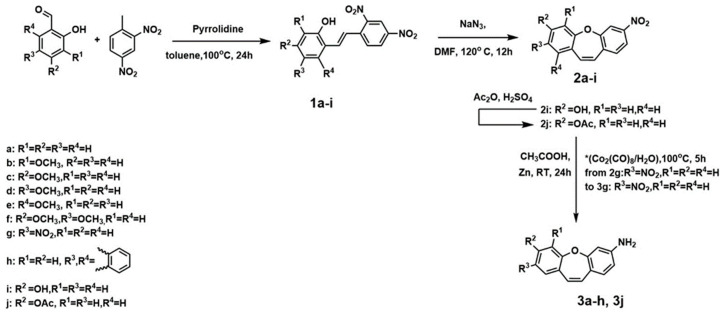
Synthesis of stilbene **1a**–**i** and dibenzo[*b,f*]oxepine **2a**–**j, 3a**–**h, 3j** derivatives.

We synthesized a series of compounds **3a**–**h**, **3j** in three synthetic steps. The synthetic routes are summarized in Scheme 1. Various stilbene moieties **1a**–**i** were obtained by starting from the condensation of suitable 2-hydroxyaldehyde and 2,4-dinitrotoluene with pyrrolidine as a catalyst to obtain the substituted stilbene. The next step was the reaction between a derivative of 2,4-dinitrostilbene **1a**–**i** and sodium azide. In the reactions, the corresponding nitrodibenzo[*b*,*f*]oxepine **2a**–**i** appeared. Nitrodibenzo[*b*,*f*]oxepine bearing a hydroxyl substituent **2i** was protected in the reaction with acetic anhydride and catalytic amounts of concentrated sulfuric acid, which yielded an acetoxy derivative of dibenzo[*b*,*f*]oxepine **2j**. Nitrodibenzo[*b*,*f*]oxepines **2a**–**f**, **2h**, **2j** were converted into amino derivatives **3a**–**f**, **3h**, **3j** in the presence of Zn in acetic acid. Nitrodibenzo[*b*,*f*]oxepine with two nitro groups **2g** was selectively reduced to **3g** by using dicobalt octocarbonyl in water.

In order to determine the structures of the reaction products of the derivatives of stilbenes **1a**–**i** and dibazo[*b*,*f*]oxepines **2a**–**j**, **3a**–**h**, **3j** in solution, the ^1^H and ^13^C NMR spectra of all of the products were measured (see Figures S8–S61 in the Supplementary Materials). The coupling constants (^1^H-^1^H) were measured directly by using the resolution-enhanced 1D spectra and were confirmed, when necessary, through homo-decoupling. The coupling constants (^1^H–^1^H) for olefin protons α and β in compounds **1a**–**i** were about 16 Hz (*E* configuration) and changed to about 11 Hz for molecules **2a**–**j**, **3a**–**h**, **3j** (*Z* configuration). In summary, we developed a method with mild conditions and operational simplicity for the versatile synthesis of derivatives of stilbenes or dibenzo[*b,f*]oxepines **1a**–**i**, **2a**–**2j**, **3a**–**h**, **3j** from substituted benzaldehydes and 2,4-dinitrotoluene.

### 2.2. Anti-Cancer Potential of Stilbenes and Oxepines

**Figure 2:**
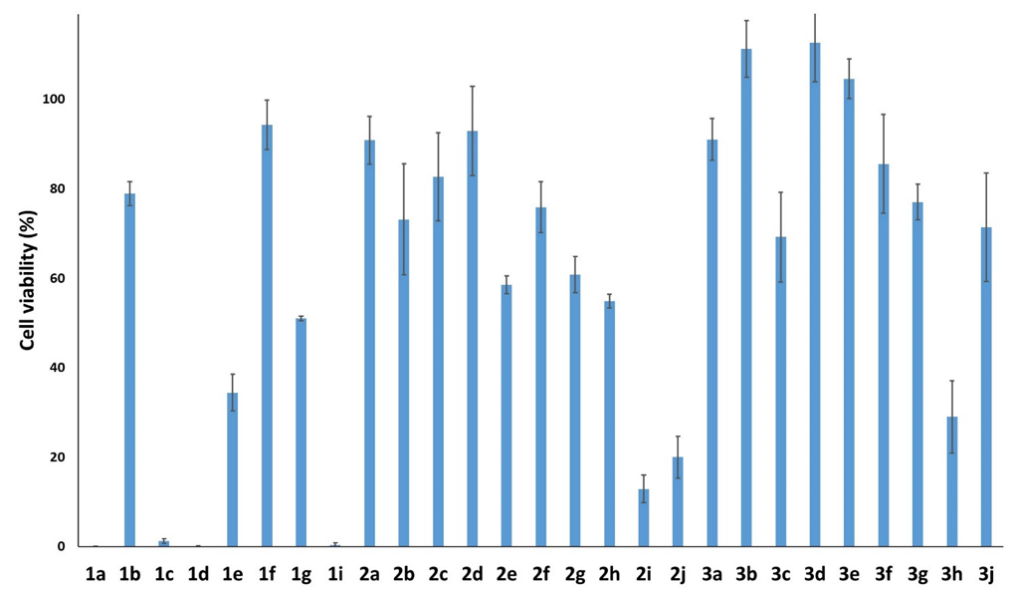
The cytotoxicity effect of tested compounds **1a**–**g**, **1i**, **2a**–**2j**, **3a**–**3h, 3j** at 100 μM on cancer HCT116 cells based on MTT assay after 48h of treatment.

To verify if the synthesized compounds showed cytotoxic effects in cancer cells, we determined the viability of compound-treated HTC116 cells by performing an MTT colorimetric assay (Figure 2) [41]. Besides compound **1a**, in which only the hydroxy group was present at position 2, the most active stilbenes were **1c**, **1d**, **1i**; e.g., compounds that were also at position 4 substituted with methoxy or hydroxy groups in **1c**, **1i** or in the compound **1d** containing a methoxy group at position 5. Generally, we observed that the stilbene derivatives were more cytotoxic than the dibenzo[*b,f*]oxepine derivatives and that the substitution of -NO_2_ with -NH_2_ decreased oxepine activity, but often improved its solubility in organic solvents (such as dimethyl sulfoxide). The opposite tendency was observed in the **2h**, **3h** series, but these were the only derivatives in which the phenyl ring was substituted by the naphthalene ring. In two series of derivatives, **1b**, **2b**, **3b** and **1f**, **2f**, **3f**, cell viability was only minimally affected. These agents had a methoxy group in the *ortho* position or two methoxy groups in the *meta* and *para* positions. Therefore, these substitutions seemed to abolish the anti-cancer activity of stilbene and oxepine derivatives.

Interestingly, a previous report on the anti-cancer activity of compound **2h** in U87 (glioblastoma) and HeLa (cervical cancer) cell lines [39] showed that it was significantly more active than in the HTC116 (colon carcinoma) cells used in this study. On the other hand, **2h** also showed severe activity in normal cell lines [39].

Among the tested compounds, four stilbenes **1a**, **1c**, **1d**, **1i** and three dibenzo[*b,f*]oxepine derivatives **2i**, **2j**, **3h** showed high potency against cancer cell lines (cell viability < 30%); hence, these molecules were selected for the next part of the study.

**Figure 3:**
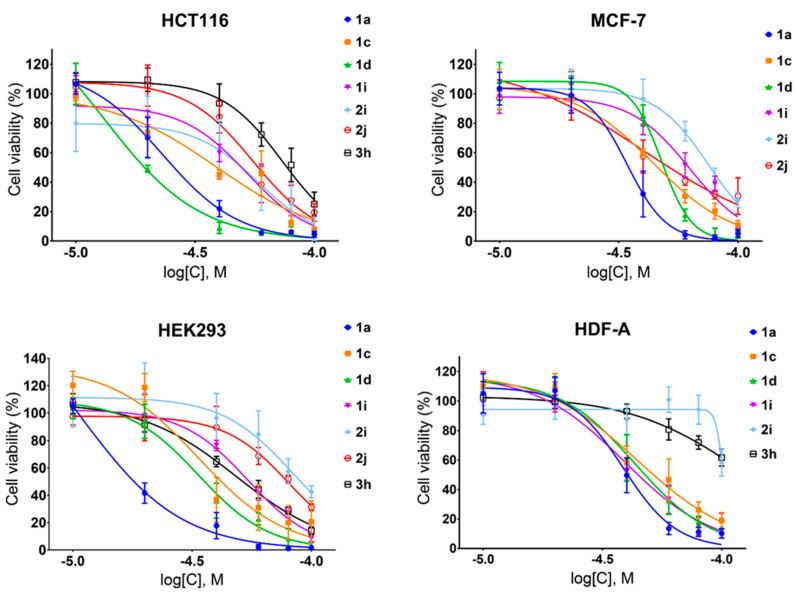
Activity of compounds **1a**, **1c**, **1d**, **1i**, **2i**, **2j**, and **3h**, in cancer cell lines (HCT116 and MCF-7) and normal cell lines (HEK293 and HDF-A) as determined with an MTT assay after 48 h of treatment. Experiments were performed in triplicate (N = 3). Data are presented as the mean ± SD.

To determine the selectivity of the chosen stilbenes and dibenzo[*b,f*]oxepine derivatives **1a**, **1c**, **1d**, **1i**, **2i**, **2j**, **3h** toward cancer cells, we estimated the cell viability in a series of increasing concentrations of these compounds by using two cancer cell lines ( human colon carcinoma (HCT116) and human breast adenocarcinoma (MCF-7)) and two normal cell lines (human embryonic kidney (HEK293) and human dermal fibroblasts (HDF-A)) (Figure 3). The tested compounds inhibited cell growth dose-dependently, and generally, a stronger effect was observed for HCT166 cells than for MCF-7 cells and for HEK293 cells than for HDF-A cells.

Next, based on the data presented above, we calculated the half-maximal inhibitory concentration (IC_50_) and selectivity index (SI). As observed here (Figure 2) and in previous studies [39], in most cases, stilbenes exhibited stronger cytotoxicity than dibenzo[*b,f*]oxepines did (Figure 3), but, with the exception of compound **1d**, they showed no selectivity toward cancer cells (Table 1). The highest selectivity indexes were obtained for stilbene **1d** and dibenzo[*b,f*]oxepine derivatives **2i**, **2j**, which varied between 1.4 and 2.4 (Table 1).

### 2.3. The Structure of Microtubular Cytoskeleton in HT116 Cells Treated with Compound (**1d**)

**Figure 4:**
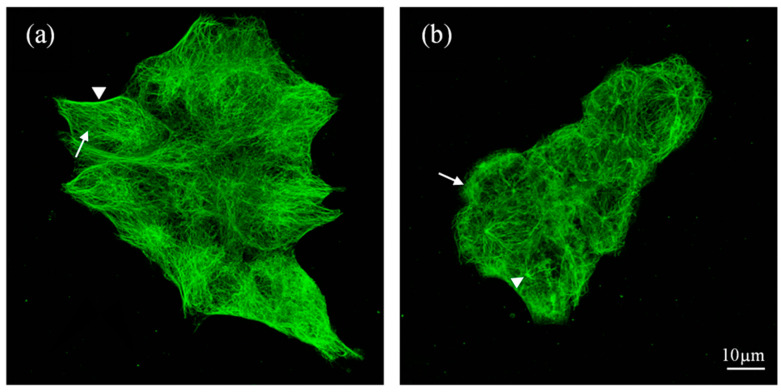
Microtubular networks in the control and **1d**-treated HTC116 cells visualized by staining with anti-α-tubulin 12g10 Ab. (**a**) Control cells—arrow: MTs arranged along the long cell axis; arrowhead: MTs lining the cell border. (**b**) Cells treated with compound **1d**—arrow: amorphous material; arrowhead: center of microtubule organization with the arising microtubules.

The IC_50_ value of stilbene **1d** obtained for HTC116 cells (18 µM) was the lowest obtained in cancer cells for all tested compounds, while the selectivity index was among the highest reached for HTC116 cells with respect to both types of control cells. Therefore, we decided to visualize the microtubular network within the control and **1d**-treated HCT116 cells. Until now, the arrangement of microtubules during treatment with dibenzo[*b,f*]oxepine derivatives has not been analyzed, but several results exist for stilbenes [42,43,44]. Immunostaining of α-tubulin showed that the control cells contained a well-developed microtubular cytoskeleton with dense microtubules that were arranged along the long axis and on the cells’ borders in elongated cells (the arrow and arrowhead, respectively, in Figure 4a).

In contrast, the HCT116 cells treated with 60 µm of compound **1d** were rounded, and the density of the microtubular network was decreased (Figure 4b). Moreover, in many cells, anti-α-tubulin stained an amorphous material, which was probably depolymerized tubulin (the arrow in Figure 4b). In contrast to the control cells, in the **1d**-treated cells, the point of the organization of MTs, which was presumably a centrosome, was well defined, with several MTs (probably the most stable) arising from it (the arrowhead in Figure 4b). This observation confirmed that compound **1d** affected the microtubular cytoskeleton, which, in turn, could lead to the cells’ death.

### 2.4. Computational Analysis and Molecular Docking Simulations

**Figure 5:**
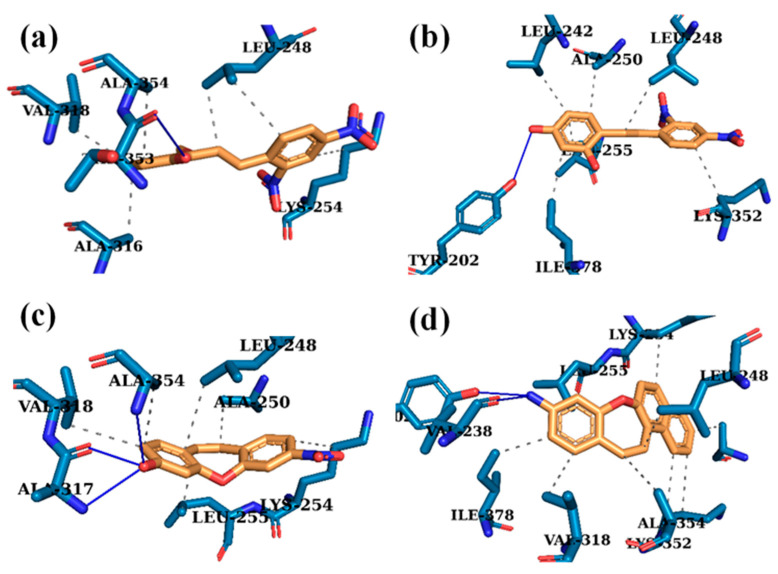
Stick representation of the top-ranked docking poses (orange) and predicted interactions of compounds (**a**) **1d**, (**b**) **1i**, (**c**) **2i**, and (**d**) **3h** with αβ-tubulin (PDB code: 1SA0). For clarity, only amino acids interacting with ligands are shown. Blue lines represent hydrogen bond interactions; dashed gray lines represent hydrophobic interactions.

The combination of experimental and computational methodologies has been of considerable importance in the discovery and further improvement of new candidates for potent anti-cancer compounds [45,46]. Molecular docking methods rely on the exploration of the ligand conformation within the active site of a protein or macromolecule until the minimum energy is achieved. Notably, this technique allows the computation of the binding free energy by including processes that are responsible for molecular recognition [47,48]. As mentioned earlier, in the Z derivatives of the stilbenes combretastatin CA1 and CA4 phosphate (**CA1P** and **CA4P**, respectively) and their disodium salts (**OXi4503** and **fosbretabulin**, respectively), as well as dibenzo[*b,f*]oxepines, which have a (*Z*)-stilbene in their skeleton, the motif docks at the colchicine-binding site [9,49]. Based on the available literature and our cytotoxicity results, we expected that selected stilbene and dibenzo[*b,f*]oxepine derivatives could be potent tubulin inhibitors that bind to colchicine pockets [39,50,51,52,53]. Therefore, the interactions between the most active compounds **1a**, **1c**, **1d**, **1i**, **2i**, **2j**, **3h** and tubulin heterodimer (crystal structure from PDB: 1SA0) were analyzed with molecular modeling. The optimal structures of the compounds with the best biological activity were calculated by using the DFT B3LYP/6-311G 6-311++g (2d,p) method (and with a polarizable continuum model (PCM), Gaussian 03W) [54,55] (Figures S1–S7, Tables S1–S7). Molecular docking was performed by simulating the incorporation of compounds **1a**, **1c**, **1d**, **1i**, **2i**, **2j**, **3h** into the colchicine-binding site in tubulin (Figure 5, Table S8). The docking protocol was validated by re-docking the DAMA–colchicine that was extracted from PDB crystal structure. In the crystal structure, the tropolone ring of colchicine is stabilized by van der Waals contacts with Val181, Ser178, and Val315. The carbonyl group is stabilized by a hydrogen bond with Val181. The ring with trimethoxy substituents is buried in the aliphatic part confined by Lys352, Asn350, Leu378, Ala316, Leu255, Lys254, Ala250, and Leu242. The methoxy group at position 3 participated in a hydrogen bond with the -SH group of Cys241 [56]. The algorithm that was applied correctly reproduced the binding mode of the native ligand. The binding poses of all tested compounds exhibited a high affinity for the target protein. The estimated binding free energy varied from −7.7 to −9.3 kcal/mol, in contrast to the −8.9 kcal/mol of redocked DAMA–colchicine (for a detailed list of the estimated binding free energies and the predicted interactions, see the supporting information). Analysis of the binding model of the most active compounds and tubulin allowed the discovery of several interactions with the protein residues in the colchicine-binding site (Table S8). Compounds **1a**, **1c**, **1d**, **1i**, **2i**, **2j**, **3h** were stabilized in their binding poses by hydrophobic interactions with β-tubulin residues (Leu242, Leu248, Ala250, Lys254, Leu255, Lys352, Ile378, Val318). Of the active compounds, four ligands (**1d**, **1i**, **2i**, **3h**) were stabilized in their binding poses by hydrogen bond interactions with the tubulin heterodimer (Figure 5 and Figure S3). However, no hydrogen bonds with Cys241 were predicted, though these are considered to be the most crucial for high potency. This may partially justify the high micromolar cytotoxicity of the tested compounds [57,58].

## 3. Conclusions

In this study, we report the synthesis and cytotoxic effects of nine stilbenoids **1a**–**i** and nineteen dibenzo[*b,f*]oxepine derivatives **2a**–**i**, **3a**–**h**, **3j**. The compounds were obtained by substituting a methoxy group in various positions of the 2-hydroxy-2′,4′-dinitrostilbene skeleton or nitro, hydroxy, acetoxy, or benzo groups of stilbene or dibenzo[*b,f*]oxepine. For the most promising compounds, we performed molecular docking, anti-cancer activity, measurements, and MT visualization through immunofluorescence. Our results showed that the stilbene derivatives were more active than the dibenzo[*b,f*]oxepine derivatives. The most active compounds were **1a**, **1c**, **1d**, **1i**, **2i**, **2j**, and **3h**.

It is worth noting that for both stilbenes and the nitro or amine dibenzo[*b,f*]oxepines, the molecular skeletons were the same, and the compounds differed in their substituents and the positions that they took. Some regularities could be observed: The introduction of electron-donating (EDG; enriched electron density in the aromatic ring) hydroxy, methoxy, or acetyloxy groups into the ring in the *para* or *ortho* position in **1c**, **1i**, **1a** and in the *meta* position in **1d** had a positive effect on the anticancer activity of both stilbene and nitro dibenzo[*b,f*]oxepine **2i**, **2j**. The last set of molecules consisted of amine dibezo[*b,f*]oxepines **3a**–**3h**, **3j**. In this case, only compound **3h** showed an effect, and there were little data for investigating the effect of the electron density of the substituent on anticancer activity.

The selectivity toward cancer cells (HCT116 and MCF-7) was investigated in comparison with the selectivity toward normal cells (HEK293 and HDF-A) for stilbenes **1a**, **1c**, **1d**, **1i** and dibenzo[*b,f*]oxepines **2i**, **2j**, **3h**; this indicated that the three most promising anti-cancer agents were compounds **1d**, **2i**, and **2j**. The molecular docking and visualization of the microtubular cytoskeleton in the stilbene and/or dibenzo[*b,f*]oxepine derivatives confirmed that the cytotoxicity was related to their tubulin-targeting abilities and, as a consequence, depolymerization of cytoplasmic MTs.

## 4. Materials and Methods

### 4.1. Chemistry

#### 4.1.1. PROCEDURE A—Obtained Compounds **1a**–**1i** (Figure 6)

To a stirred mixture of 2,4-dinitrotoluene (12.00 mmol), appropriate aldehyde (12.00 mmol), and toluene (20 mL) under argon, dry pyrrolidine (0.9 mL, 0.782 g, 11.00 mmol) was added. After 24 h of heating at 100 °C, the solvent was distilled off on a rotary evaporator. Then, ethyl acetate (150 mL) was added to the residue, and the resulting mixture was washed with 0.5 M hydrochloric acid (2 × 40 mL) and water to neutral pH. Next, the mixture was dried by the addition of anhydrous MgSO_4_ and the solvent was removed in vacuo. The crude product was purified by crystallization from methanol or ethanol with a small amount of the activated charcoal (in order to absorb colored impurities).

**Figure 6:**
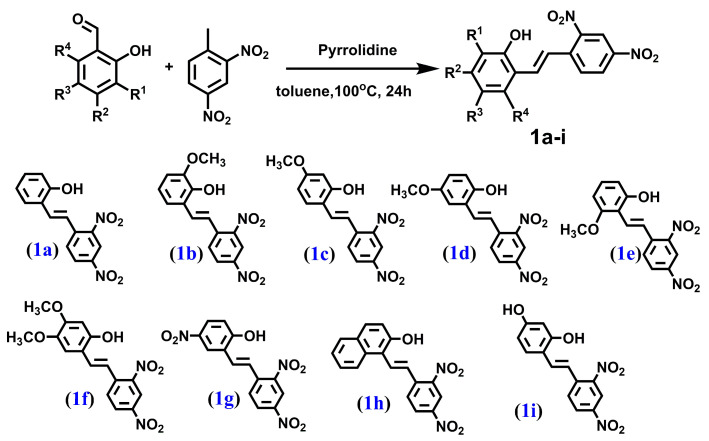
The structure of: (*E*)-2-hydroxy-2′,4′-dinitrostilbene (**1a**), (*E*)-2-hydroxy-3-methoxy-2′,4′-dinitrostilbene (**1b**), (*E*)-2-hydroxy-4-methoxy-2′,4′-dinitrostilbene (**1c**), (*E*)-2-hydroxy-5-methoxy-2′,4′-dinitrostilbene (**1d**), (*E*)-2-hydroxy-6-methoxy-2′,4′-dinitrostilbene (**1e**), (*E*)-2-hydroxy-4,5-dimethoxy-2′,4′-dinitrostilbene (**1f**), (*E*)-2-hydroxy-5,2′,4′-trinitrostilbene (**1g**), (*E*)-1-(2,4-dinitrostyryl)naphthalen-2-ol (**1h**) and (*E*)-2,4-dihydroxy-2′,4′-dinitrostilbene (**1i**).

#### 4.1.2. PROCEDURE B—Obtained Compounds **2a**–**2i** (Figure 7)

To the solution of appropriate stilbene (5.00 mol) in DMF (15 mL/mmol) NaN_3_ (9.50 mmol) was sequentially added and the reaction flask was fitted with a condenser. The mixture was stirred at 120 °C overnight and concentrated in vacuo. The residue was purified by flash column chromatography on silica gel with DCM as the mobile phase.

**Figure 7:**
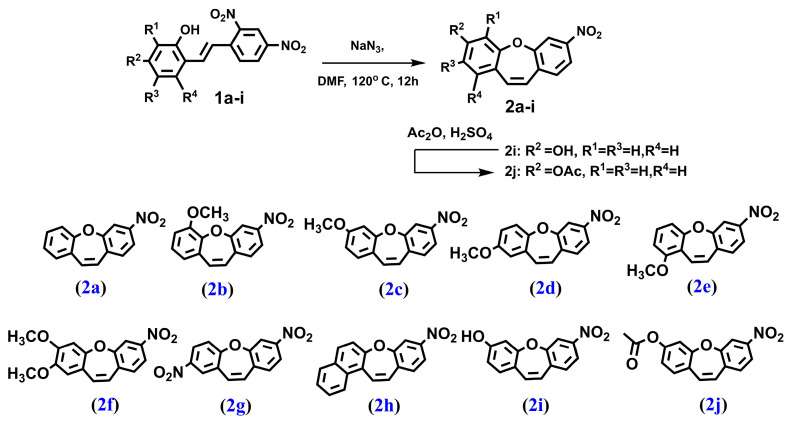
The structure of: 3-nitrodibenzo[*b,f*]oxepine (**2a**), 6-methoxy-3-nitrodibenzo[*b,f*]oxepine (**2b**), 3-methoxy-7-nitrodibenzo[*b,f*]oxepine (**2c**), 2-methoxy-7-nitrodibenzo[*b,f*]oxepine (**2d**), 1-methoxy-7-nitrodibenzo[*b,f*]oxepine (**2e**), 2,3-dimethoxy-7-nitrodibenzo[*b,f*]oxepine (**2f**), 2,7-dinitrodibenzo[*b,f*]oxepine (**2g**), 9-nitrobenzo[*b*]naphtho[*1,2-f*]oxepine (**2h**), 7-nitrodibenzo[*b,f*]oxepin-3-ol (**2i**) and 7-nitrodibenzo[*b,f*]oxepin-3-yl acetate (**2j**).

#### 4.1.3. PROCEDURE C—Obtained Compound **2j** (Figure 7)

The compound **2i** (1.00 mmol) was dissolved in excess of acetic anhydride. A few drops of concentrated sulfuric acid were added to the obtained solution and the mixture was stirred at room temperature overnight. Next, the reaction was quenched by dropwise addition of a concentrated aqueous solution of sodium bicarbonate (1 mL) and evaporated under reduced pressure. The residue was re-dissolved in ethyl acetate and transferred to a separatory funnel, washed two times with water and with brine. After solvent evaporation, the residue was purified by flash column chromatography on silica gel with a 10% mixture of ethyl acetate in hexane as a mobile phase.

#### 4.1.4. PROCEDURE D—Obtained Compound **3a**–**3f** and **3j** (Figure 8)

To the solution of corresponding nitrodibenzo[*b,f*]oxepine (1.00 mmol) in acetic acid (20.0 mL/mmol) activated zinc dust was added (10 mmol) and the resulting slurry was allowed to stir overnight at room temperature. Next, the mixture was filtrated through a pad of celite and a solvent was evaporated under reduced pressure. The residue was subjected to column chromatography on silica gel with hexane: ethyl acetate 1:1 mixture (*v*:*v*) as the eluent.

**Figure 8:**
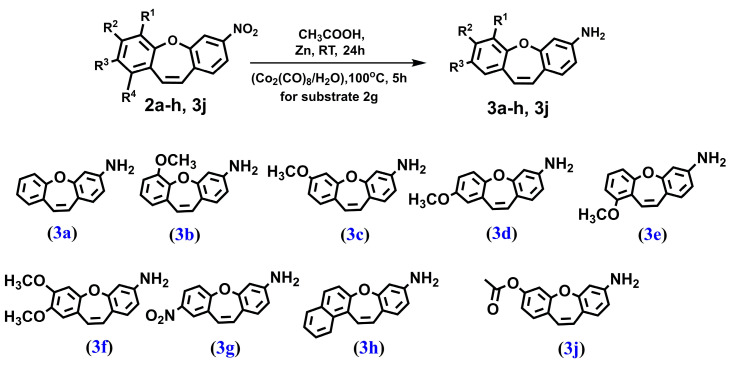
The structure of: dibenzo[*b,f*]oxepin-3-amine (**3a**), 6-methoxydibenzo[*b,f*]oxepin-3-amine (**3b**), 7-methoxydibenzo[*b,f*]oxepin-3-amine (**3c**), 8-methoxydibenzo[*b,f*]oxepin-3-amine (**3d**), 9-methoxydibenzo[*b,f*]oxepin-3-amine (**3e**), 7,8-dimethoxydibenzo[*b,f*]oxepin-3-amine (**3f**), 8-nitrodibenzo[*b,f*]oxepin-3-amine (**3g**), benzo[*b*]naphtho[*1,2-f*]oxepin-9-amine (**3h**) and 7-aminodibenzo[*b,f*]oxepin-3-yl acetate (**3j**).

#### 4.1.5. PROCEDURE E—Obtained Compound **3g** (Figure 8)

Dicobalt octacarbonyl (1.00 g, 3.00 mmol) was placed in a 50 mL three-necked flask equipped with a reflux condenser. The vessel was purged with argon, and (1.76 mmol) of 2.7-dinitrodibenzo[*b,f*]oxepine **2g** and 20 mL of dimethoxyethane were added and the solution was stirred under an argon atmosphere. After the precipitate had dissolved, 2 mL of water was added and it was heated at 100 ° C for 5 h. Next, the mixture was cooled down to room temperature and concentrated on a rotary evaporator. The resulting precipitate was dissolved in chloroform and then purified by column chromatography using chloroform as the eluent.

***(E)*-2-hydroxy-2′,4′-dinitrostilbene** (**1a**), Yield 12% (0.412 g). mp. 192 °C. **^1^H NMR** (500 MHz, DMSO-d_6_, 298 K): δ (ppm): 10.18 (br. s, 1H), 8.70 (d, *J* = 2.4 Hz, 1H), 8.44 (dd, *J* = 8.8 Hz, 1H), 8.20 (d, 1H), 7.66 (d, *J* = 16 Hz, 1H), 7.59 (d, *J* = 16 Hz, 1H), 7.54 (dd, *J* = 7.5 Hz, *J* = 1.5 Hz, 1H), 7.20 (t, *J* = 8.5 Hz, 1H), 6.90 (dd, *J* = 1 Hz. 1H), 6.85 (td, 1H). **^13^C NMR** (125 MHz, DMSO-d_6_, 298 K): δ (ppm): 156.3, 147.2, 145.5, 138.3, 133.5, 130.7, 128.8, 128.5, 127.2, 122.5, 120.7, 120.3, 119.5, 116.2.

***(E)*-2-hydroxy-3-methoxy-2′,4′-dinitrostilbene (1b)**, Yield 50% (1.896 g). mp. 182 °C. **^1^H NMR** (500 MHz, CDCl_3_, 298 K): δ (ppm): 8.79 (d, *J* = 2 Hz, 1H), 8.40 (dd, *J* = 8.8 Hz, 1H), 8.03 (d, *J* = 8.8 Hz, 1H), 7.72 (d, *J* = 16 Hz, 1H), 7.62 (d, *J* = 16 Hz, 1H), 7.19 (dd, *J* = 7.5 Hz, *J* = 2 Hz, 1H), 6.90 (dd, *J* = 8 Hz, 1H), 6.87 (dd, 1H), 3.94 (s, 3H). **^13^C NMR** (125 MHz, CDCl_3_, 298 K): δ (ppm): 146.8, 144.7, 139.4, 132.7, 128.9, 126.9, 121.9, 121.7,120.6, 120.0, 119.6, 111.3, 56.2. **HRMS** (ESI-) calc. for [M-H]^−^ (C_15_H_11_N_2_O_6_): 315.0623, found 315.0624

***(E)*-2-hydroxy-4-methoxy-2′,4′-dinitrostilbene (1c)**, Yield 53% (2.01 g). mp. 175 °C. **^1^H NMR** (500 MHz, CDCl_3_, 298 K): δ (ppm): 8.75 (d, *J* = 2.4 Hz, 1H), 8.35 (dd, *J* = 8.8 Hz, *J* = 2.4 Hz, 1H), 7.88 (d, *J* = 8.8 Hz, 1H), 7.57 (s, 2H), 7.50 (d, *J* = 8.8 Hz, 1H), 6.54 (dd, *J* = 7.7 Hz, *J* = 2.4 Hz, 1H), 3.81 (s, 3H). **^13^C NMR** (125 MHz, CDCl_3_, 298 K): δ (ppm): 162.0, 155.6, 147.0, 145.4, 139.7, 133.0, 129.4, 128.4, 126.8, 120.7, 118.9, 116.25, 107.3, 102.0, 55.5. **HRMS** (ESI-) calc. for [M-H]^−^ (C_15_H_11_N_2_O_6_): 315.0623, found 315.0622.

***(E)*-2-hydroxy-5-methoxy-2′,4′-dinitrostilbene (1d)**, Yield 56% (2.123 g). mp. 168 °C. **^1^H NMR** (500 MHz, DMSO-d_6_, 298 K): δ (ppm): 9.75 (br. s, 1H), 8.73 (d, *J* = 2.5 Hz, 1H), 8.47 (dd, *J* = 8.5 Hz, 1H), 8.21 (d, 1H), 7.65 (d, *J* = 11.5 Hz, 1H), 7.61 (d, 1H), 7.12 (t, *J* = 2 Hz, 1H), 6.84 (d, 2H), 3.72 (s, 3H). **^13^C NMR** (125 MHz, DMSO-d_6_, 298 K): δ (ppm): 152.3, 150.4, 147.2, 145.6, 138.3, 133.4, 128.9, 127.2, 122.8, 121.1, 120.3, 117.19, 117.02, 112.27, 30.70. **HRMS** (ESI-) calc. for [M-H]^−^ (C_15_H_11_N_2_O_6_) 315.0623, found: 315.0624.

***(E)*-2-hydroxy-6-methoxy-2′,4′-dinitrostilbene (1e)**, Yield 69% (2.616 g). mp. 212 °C. **^1^H NMR** (500 MHz, DMSO-d_6_, 298 K): δ (ppm): 10.33 (s, 1H), 8.72 (d, *J* = 2.5 Hz, 1H), 8.44 (dd, *J* = 9 Hz, 1H), 8.14 (d, 1H), 7.96 (d, *J* = 16.5 Hz, 1H), 7.71 (d, 1H), 7.16 (t, *J* = 8.5 Hz, 1H), 6.56 (dd, *J* = 0.5 Hz, 1H), 6.54 (dd, 1H), 3.84 (s, 3H). **^13^C NMR** (125 MHz, DMSO-d_6_, 298 K): δ (ppm): 159.1, 157.7, 147.1, 145.2, 139.2, 130.8, 129.6, 128.1, 127.1, 122.2, 120.1, 111.3, 108.7, 102.2, 55.8. **HRMS** (ESI-) calc. for [M-H]^−^ (C_15_H_11_N_2_O_6_) 315.0623, found: 315.0625.

***(E)*-2-hydroxy-4,5-dimethoxy-2′,4′-dinitrostilbene (1f)**, Yield 75% (3.114 g). mp. 221 °C. **^1^H NMR** (500 MHz, DMSO-d_6_, 298 K): δ (ppm): 9.91 (s, 1H), 8.69 (d, *J* = 2.4 Hz, 1H), 8.42 (dd, *J* = 8.8 Hz, *J* = 2.4 Hz, 1H), 8.17 (d, *J* = 8.9 Hz, 1H), 7.67 (d, *J* = 16.1 Hz, 1H), 7.46 (d, *J* = 16.1 Hz, 1H), 7.11 (s, 1H), 6.53 (s, 1H), 3.76 (s, 3H), 3.73 (s, 3H). **^13^C NMR** (125 MHz, DMSO-d_6_, 298 K): δ (ppm): 151.8, 151.6, 146.8, 144.8, 142.3, 138.8, 133.7, 128.1, 127.0, 120.4, 117.5, 113.8, 111.3, 100.5, 56.2, 55.4, 40.0, 39.9, 39.7, 39.5, 39.4, 39.2, 39.0. **HRMS** (ESI+) calc. for [M+H]^+^ (C_16_H_15_N_2_O_7_): 347.0874, found 347.0867.

***(E)*-2-hydroxy-5,2′,4′-tridinitrostilbene (1g),** Yield 8% (0.317 g). mp. 241 °C. **^1^H NMR** (500 MHz, DMSO-d_6_, 298 K): δ (ppm): 8.73 (d, *J* = 2.3 Hz, 1H), 8.54 − 8.44 (m, 2H), 8.24 (d, *J* = 8.8 Hz, 1H), 8.11 (dd, *J* = 9.0 Hz, *J* = 2.8 Hz, 1H), 7.84 (d, *J* = 16.3 Hz, 1H), 7.64 (d, *J* = 16.3 Hz, 1H), 7.07 (d, *J* = 9.0 Hz, 1H). **^13^C NMR** (125 MHz, DMSO-d_6_, 298 K): δ (ppm): 162.3, 147.5, 146.0, 139.8, 137.8, 131.3, 129.2, 127.3, 126.0, 124.7, 124.0, 123.2, 120.2, 116.6, 40.0, 39.9, 39.7, 39.5, 39.4, 39.2, 39.0. **HRMS** (ESI+) calc. for [M+H]^+^ (C_14_H_9_N_3_O_7_): 331.0440, found 331.0438.

***(E)*-1-(2,4-dinitrostyryl)naphthalen-2-ol (1h)**, Not isolated. Crude product mixture was used as a substrate in a subsequent reaction.

***(E)*-2,4-dihydroxy-2′,4′-dinitrostilbene (1i)**, Yield 12% (0.434 g). mp. 201 °C. **^1^H NMR** (500 MHz, DMSO-d_6_, 298 K): δ (ppm): 10.13 (s, 1H), 9.83 (s, 1H), 8.69 (d, *J* = 2.4 Hz, 1H), 8.40 (dd, *J* = 8.9 Hz, *J* = 2.4 Hz, 1H), 8.17 (d, *J* = 8.9 Hz, 1H), 7.63 (d, *J* = 16.1 Hz, 1H), 7.42 (d, *J* = 16.1 Hz, 1H), 7.38 (d, *J* = 8.6 Hz, 1H), 6.37 (d, *J* = 2.3 Hz, 1H), 6.31 (dd, *J* = 8.5 Hz, *J* = 2.3 Hz, 1H). **^13^C NMR** (125 MHz, DMSO-d_6_, 298 K): δ (ppm): 160.3, 158.0, 146.7, 144.6, 138.9, 134.5, 130.1, 127.9, 126.9, 120.3, 116.4, 114.4, 107.9, 102.6. **HRMS** (ESI+) calc. for [M+H]^+^ (C_16_H_15_N_2_O_7_): 302.0539, found 302.0543.

**3-nitrodibenzo*[b,f]*oxepine (2a)**, Yield 95% (1.135 g). mp. 156 °C. **^1^H NMR** (500 MHz, DMSO-d_6_, 298 K): δ (ppm): 8.10 (d, *J* = 2.5 Hz, 1H), 8.04 (dd, *J* = 8.5 Hz, *J* = 2.4 Hz, 1H), 7.57 (d, *J* = 8.5 Hz, 1H), 7.44 (ddd, *J* = 7.5 Hz, *J* = 7 Hz, *J* = 1.5 Hz, 1H), 7.40 (dd, *J* = 1.5 Hz, 1H), 7.36 (dd, *J* = 8 Hz, 1H), 7.24 (ddd, 1H), 7.03 (d, *J* = 11.5 Hz, 1H), 6.92 (d, *J* = 11.5 Hz, 1H). **^13^C NMR** (125 MHz, DMSO-d_6_, 298 K): δ (ppm): 156.0, 155.9, 148.2, 137.1, 133.5, 131.2, 130.2, 129.9, 129.5, 128.2, 125.8, 121.4, 120.4, 116.5.

**6-methoxy-3-nitrodibenzo*[b,f]*oxepine (2b)**, Yield 99% (1.331 g). mp. 184 °C. **^1^H NMR** (500 MHz, CDCl_3_, 298 K): δ (ppm): 8.16 (d, *J* = 2.3 Hz, 1H), 7.98 (dd, *J* = 8.5 Hz, *J* = 2.3 Hz, 1H), 7.30 (d, *J* = 8.5 Hz, 1H), 7.10 (dd, *J* = 8.1 Hz, *J* = 7.7 Hz, 1H), 6.99 (dd, *J* = 8.2 Hz, *J* = 1.5 Hz, 1H), 6.90 (d, *J* = 11.4 Hz, 1H), 6.80 (1H, dd, H_9_), 6.77 (d, *J* = 11.4 Hz, 1H), 3.96 (s, 3H). **^13^C NMR** (125 MHz, CDCl_3_, 298 K): δ (ppm): 156.9, 151.8, 148.4, 144.7, 137.5, 133.5, 131.0, 129.1, 128.2, 125.5, 120.8, 112.0, 117.6, 113.3, 56.2.

**3-methoxy-7-nitrodibenzo*[b,f]*oxepine (2c)**, Yield 98% (1.318 g). mp. 189 °C. **^1^H NMR** (500 MHz, CDCl_3_, 298 K): δ (ppm): 8.00 (d, *J* = 2.3 Hz, 1H), 7.97 (dd, *J* = 8.4 Hz, *J* = 2.3 Hz, 1H), 7.26 (d, *J* = 8.5 Hz, 1H), 7.09 (d, *J* = 8.5 Hz, 1H), 6.79 (d, *J* = 11.4 Hz, 1H), 6.76 (d, *J* = 2.5 Hz, 2H), 6.72 (dd, *J* = 8.4 Hz, *J* = 2.5 Hz, 1H), 6.58 (d, *J* = 11.3 Hz, 2H), 3.84 (s, 3H). **^13^C NMR** (125 MHz, CDCl_3_, 298 K): δ (ppm): 162.4, 157.8, 156.3, 148.2, 137.8, 133.5, 130.6, 129.3, 125.7, 122.4, 120.1, 117.1, 111.7, 106.9, 55.6.

**2-methoxy-7-nitrodibenzo*[b,f]*oxepine (2d)**, Yield 98% (1.318 g). mp. 167 °C. **^1^H NMR** (500 MHz, CDCl_3_, 298 K): δ (ppm): 7.03 (d, *J* = 8.5 Hz, 1H), 6.93 (d, *J* = 8 Hz, 1H), 6.77 (dd, *J* = 3 Hz, 1H), 6.63 (d, 1H), 6.59 (d, *J* = 11 Hz, 1H), 6.49 (d, 1H), 6.45 (d, 1H), 6.41 (dd, 1H), 3.76 (s, 3H). **^13^C NMR** (125 MHz, CDCl_3_, 298 K): δ (ppm): 158.9, 156.4, 150.5, 148.7, 131.7, 130.6, 130.5, 126.4, 121.9, 120.9, 114.5, 113.4, 111.3, 107.5, 55.6. **HRMS** (ESI+) calc. for [M]^+^ (C_15_H_11_NO_4_) 269.0688, found: 269.0690.

**1-methoxy-7-nitrodibenzo*[b,f]*oxepine (2e)**, Yield 96% (1.291 g). mp. 178 °C. **^1^H NMR** (500 MHz, CDCl_3_, 298 K): δ (ppm): 8.00 (d, *J* = 2.5 Hz, 1H), 7.97 (dd, *J* = 8.5 Hz, 1H), 7.31 (t, *J* = 8.5 Hz, 1H), 7.27 (d, 1H), 7.21 (d, *J* = 11.5 Hz, 1H), 6.85 (dd, *J* = 0.5 Hz, 1H), 6.74 (d, 1H), 6.49 (dd, 1H), 3.86 (s, 3H). **^13^C NMR** (125 MHz, CDCl_3_, 298 K): δ (ppm): 158.5, 157.4, 156.9, 148.3, 138.0, 131.2, 129.2, 128.6, 127.0, 120.1, 119.0, 117.0, 113.6, 107.6, 56.0. **HRMS** (ESI+) calc. for [M]^+^ (C_15_H_11_NO_4_) 269.0688, found: 269.0683.

**2,3-dimethoxy-7-nitrodibenzo*[b,f]*oxepine (2f)**, Yield 96% (1.435 g). mp. 165 °C. **^1^H NMR** (500 MHz, CDCl_3_, 298 K): δ (ppm): 8.00 (d, *J* = 2.3 Hz, 1H), 7.96 (dd, *J* = 8.4 Hz, *J* = 2.3 Hz, 1H), 7.25 (d, *J* = 8.4 Hz, 1H), 6.78 (s, 1H), 6.77 (d, *J* = 11.2 Hz, 1H), 6.65 (d, *J* = 11.2 Hz, 1H), 6.63 (s, 1H), 3.93 (s, 3H), 3.85 (s, 3H). **^13^C NMR** (125 MHz, CDCl_3_, 298 K): δ (ppm): 156.7, 151.3, 150.4, 148.3, 146.5, 137.6, 133.5, 129.3, 126.5, 121.3, 120.0, 116.9, 111.4, 105.2, 56.3, 56.2. **HRMS** (ESI+) calc. for [M]^+^ (C_16_H_13_NO_5_): 299.0794, found 299.0786.

**2,7-dinitrodibenzo*[b,f]*oxepine (2g),** Yield 80% (1.136 g). mp. 216 °C. **^1^H NMR** (500 MHz, DMSO-d_6_, 298 K): δ (ppm): 8.32 (d, *J* = 2.8 Hz, 1H), 8.29 (dd, *J* = 8.8 Hz, *J* = 2.9 Hz, 1H), 8.23 (d, *J* = 2.4 Hz, 1H), 8.10 (dd, *J* = 8.5 Hz, *J* = 2.4 Hz, 1H), 7.68 (d, *J* = 8.8 Hz, 1H), 7.63 (d, *J* = 8.5 Hz, 1H), 7.17 (d, *J* = 11.5 Hz, 1H), 7.08 (d, *J* = 11.5 Hz, 1H). **^13^C NMR** (125 MHz, DMSO-d_6_, 298 K): δ (ppm): 160.4, 155.2, 148.6, 145.1, 136.3, 131.7, 130.7, 130.6, 130.2, 126.2, 125.3, 123.0, 121.0, 117.0. **HRMS** (ESI+) calc. for [M]^+^ (C_14_H_8_N_2_O_5_): 284.0433, found 284.0439.

**9-nitrobenzo[*b*]naphtho [*1,2-f*]oxepine (2h),** Yield 98% (1.416 g). mp. 197 °C. **^1^H NMR** (500 MHz, CDCl_3_, 298 K): δ (ppm): 8.09 (d, *J* = 2.3 Hz, 1H), 8.07 − 8.03 (m, 1H), 8.00 (dd, *J* = 8.4, 2.3 Hz, 1H), 7.89 (d, *J* = 8.8 Hz, 1H), 7.86 − 7.81 (m, 1H), 7.65 (d, *J* = 11.6 Hz, 1H), 7.57 (ddd, *J* = 8.4 Hz, *J* = 6.9 Hz, *J* = 1.4 Hz, 1H), 7.48 (ddd, *J* = 8.0 Hz, *J* = 6.9 Hz, *J* = 1.1 Hz, 1H), 7.42 (d, *J* = 8.8 Hz, 1H), 7.35 (d, *J* = 8.5 Hz, 1H), 7.04 (d, *J* = 11.6 Hz, 1H). **^13^C NMR** (125 MHz, CDCl_3_, 298 K): δ (ppm): 157.6, 155.9, 148.6, 137.8, 131.8, 131.5, 131.4, 130.0, 129.1, 129.0, 128.6, 127.4, 125.6, 123.3, 123.2, 120.9, 120.2, 116.9. **HRMS** (ESI+) calc. for [M+H]^+^ (C_16_H_15_N_2_O_7_): 289.0739, found 289.0728.

**7-nitrodibenzo*[b,f]*oxepin-3-ol (2i),** Yield 30% (0.382 g). mp. 164 °C. **^1^H NMR** (500 MHz, DMSO-d_6_, 298 K): δ (ppm): 10.10 (s, 1H), 8.02 (d, *J* = 2.4 Hz, 1H), 7.99 (dd, *J* = 8.4 Hz, *J* = 2.4 Hz, 1H), 7.47 (d, *J* = 8.5 Hz, 1H), 7.13 (d, *J* = 8.4 Hz, 1H), 6.86 (d, *J* = 11.3 Hz, 1H), 6.75 (d, *J* = 2.4 Hz, 1H), 6.67 (d, *J* = 11.3 Hz, 1H), 6.63 (dd, *J* = 8.4 Hz, *J* = 2.4 Hz, 1H). **^13^C NMR** (125 MHz, DMSO-d_6_, 298 K): δ (ppm): 160.8, 157.2, 155.3, 147.7, 137.8, 133.7, 130.9, 129.8, 124.8, 120.7, 120.3, 116.6, 112.9, 108.4. **HRMS** (ESI-) calc. for [M-H]^−^ (C_14_H_8_NO_4_): 254.0459, found 254.0456.

**7-nitrodibenzo*[b,f]*oxepin-3-yl acetate (2j)**, Yield 63% (0.187 g). mp. 133 °C. **^1^H NMR** (500 MHz, DMSO-d_6_, 298 K): δ (ppm): 7.96 − 7.93 (m, 2H), 7.25 (d, *J* = 9.1 Hz, 1H), 7.17 (d, *J* = 8.4 Hz, 1H), 7.00 (d, *J* = 2.2 Hz, 1H), 6.93 (dd, *J* = 8.3 Hz, *J* = 2.3 Hz, 1H), 6.80 (d, *J* = 11.4 Hz, 1H), 6.66 (d, *J* = 11.4 Hz, 1H), 2.31 (s, 3H). **^13^C NMR** (125 MHz, DMSO-d_6_, 298 K): δ (ppm): 169.0, 156.9, 156.4, 152.6, 148.3, 137.0, 132.7, 130.1, 129.5, 127.8, 127.4, 120.2, 118.9, 117.0, 115.2, 21.02. **HRMS** (ESI+) calc. for [M+H]^+^ (C_16_H_12_NO_5_): 298.0710, found 298.0709.

**Dibenzo*[b,f]*oxepin-3-amine (3a)**, Yield 59% (0.123 g). mp. 155 °C. **^1^H NMR** (500 MHz, CDCl_3_, 298 K): δ (ppm): 7.25 − 7.22 (m, 1H), 7.14 − 7.04 (m, 3H), 6.93 (d, *J* = 8.2 Hz, 1H), 6.58 (d, *J* = 11.3 Hz, 1H), 6.51 (d, *J* = 2.5 Hz, 1H), 6.50 (d, *J* = 11.3 Hz, 1H), 6.42 (dd, *J* = 8.2 Hz, *J* = 2.3 Hz, 1H). **^13^C NMR** (125 MHz, CDCl_3_, 298 K): δ (ppm): 158.6, 156.7, 148.6, 131.1, 130.4, 130.1, 129.2, 129.1, 126.5, 124.8, 121.3, 121.1, 111.4, 107.7. **HRMS** (ESI+) calc. for [M+H]^+^ (C_14_H_12_NO): 210.0913, found 210.0910.

**6-methoxydibenzo*[b,f]*oxepin-3-amine (3b)**, Yield 85% (0.203 g). mp. 179 °C. **^1^H NMR** (400 MHz, CDCl_3_, 298 K): δ (ppm): 7.02 (t, *J* = 7.9 Hz, 1H), 6.95 (d, *J* = 8.1 Hz, 1H), 6.87 (dd, *J* = 8.1 Hz, *J* =1.2 Hz, 1H), 6.73 (dd, *J* = 7.7 Hz, *J* = 1.1 Hz, 1H), 6.66 (d, *J* = 2.0 Hz, 1H), 6.64 (d, *J* = 11.2 Hz, 1H), 6.53 (d, *J* = 11.2 Hz, 1H), 6.43 (dd, *J* = 8.2 Hz, *J* = 2.3 Hz, 1H), 3.91 (s, 3H), 3.80 (br. s, 2H). **^13^C NMR** (100 MHz, CDCl_3_, 298 K): δ (ppm): 158.4, 151.7, 148.6, 144.7, 132.4, 130.3, 130.0, 126.4, 124.6, 121.3, 120.5, 111.7, 111.4, 108.3, 56.1. **HRMS** (ESI+) calc. for [M+H]^+^ (C_15_H_14_NO_2_): 240.1019, found 240.1016.

**7-methoxydibenzo*[b,f]*oxepin-3-amine (3c)**, Yield 84% (0.200 g). mp. 156 °C. **^1^H NMR** (400 MHz, CDCl_3_, 298 K): δ (ppm):7.02 (d, *J* = 8.4 Hz, 1H), 6.92 (d, *J* = 8.2 Hz, 1H), 6.69 (d, *J* = 2.6 Hz, 1H), 6.65 (dd, *J* = 8.4 Hz, *J* = 2.6 Hz, 1H), 6.52 (d, *J* = 2.3 Hz, 1H), 6.49 − 6.41 (m, 3H), 3.80 (s, 3H). **^13^C NMR** (100 MHz, CDCl_3_, 298 K): δ (ppm):160.9, 157.9, 157.6, 147.8, 130.2, 129.7, 127.8, 126.4, 123.7, 121.5, 111.7, 110.8, 107.9, 106.8, 55.5. **HRMS** (ESI+) calc. for [M+H]^+^ (C_15_H_14_NO_2_): 240.1019, found 240.1015.

**8-methoxydibenzo*[b,f]*oxepin-3-amine (3d)**, Yield 84% (0.200 g). mp. 158 °C. **^1^H NMR** (500 MHz, CDCl_3_, 298 K): δ (ppm): 7.03 (d, *J* = 8.5 Hz, 1H), 6.93 (d, *J* = 8 Hz, 1H), 6.77 (dd, *J* = 3 Hz, 1H), 6.63 (d, 1H), 6.59 (d, *J* = 11 Hz, 1H), 6.49 (d, 1H), 6.45 (d, 1H), 6.41 (dd, 1H), 3.76 (s, 3H). **^13^C NMR** (125 MHz, CDCl_3_, 298 K): δ (ppm): 158.9, 156.4, 150.5, 148.7, 131.7, 130.6, 130.5, 126.4, 121.9, 120.9, 114.5, 113.4, 111.3, 107.5, 55.6. **HRMS** (ESI+) calc. for [M+H]^+^ (C_15_H_13_NO_2_+H) 240.1019, found: 240.1019.

**9-methoxydibenzo*[b,f]*oxepin-3-amine (3e)**, Yield 71% (0.170 g). mp. 170 °C. **^1^H NMR** (500 MHz, CDCl_3_, 298 K): δ (ppm): 7.19 (t, *J* = 8.5 Hz, 1H), 6.94 (d, *J* = 8 Hz, 1H), 6.84 (d, *J* = 11.5 Hz, 1H), 6.77 (dd, *J* = 0.5 Hz, 1H), 6.65 (dd, 1H), 6.64 (d, 1H), 6.49 (1H, d, *J* = 2.5 Hz, H_6_), 6.42 (dd, 1H), 3.83 (s, 3H). **^13^C NMR** (125 MHz, CDCl_3_, 298 K): δ (ppm): 158.6, 158.5, 157.0, 148.5, 130.1, 129.3, 129.3, 121.6, 121.3, 120.2, 113.8, 111.5, 107.6, 106.9, 55.9. **HRMS** (ESI+) calc. for [M+H]^+^ (C_15_H_13_NO_2_+H) 240.1019, found: 240.1019.

**7,8-dimethoxydibenzo*[b,f]*oxepin-3-amine (3f)**, Yield 29% (0.078 g). mp. 223 °C. **^1^H NMR** (400 MHz, CDCl_3_, 298 K): δ (ppm): 6.93 (d, *J* = 8.2 Hz, 1H), 6.69 (s, 1H), 6.59 (s, 1H), 6.56 − 6.48 (m, 2H), 6.48 − 6.38 (m, 2H), 3.88 (s, 3H), 3.83 (s, 3H). **^13^C NMR** (100 MHz, CDCl_3_, 298 K): δ (ppm): 158.2, 150.1, 149.7, 147.7, 145.8, 130.3, 128.6, 126.4, 122.5, 121.5, 111.8, 110.8, 107.8, 105.1, 56.1, 56.0. **HRMS** (ESI+) calc. for [M+H]^+^ (C_16_H_16_NO_3_): 270.1125, found 270.1121.

**8-nitrodibenzo*[b,f]*oxepin-3-amine (3g),** Yield 72% (0.322 g). m.p. 138 °C. **^1^H NMR** (500 MHz, DMSO-d_6_, 298 K): δ (ppm): 8.14 – 8.10 (m, 2H), 7.33 (d, *J* = 9.7 Hz, 1H), 6.94 (d, *J* = 8.3 Hz, 1H), 6.68 (d, *J* = 11.3 Hz, 1H), 6.53 (d, *J* = 11.3 Hz, 1H), 6.41 (d, *J* = 2.2 Hz, 1H), 6.38 (dd, *J* = 8.2 Hz, *J* = 2.2 Hz, 1H). **^13^C NMR** (100 MHz, DMSO-d_6_, 298 K): δ (ppm): 160.3, 157.0, 152.3, 144.4, 132.7, 132.4, 130.8, 124.3, 124.2, 122.6, 122.6, 116.9, 110.8, 105.7. **HRMS** (ESI+) calc. for [M+H]^+^ (C_14_H_11_N_2_O_3_): 255.0764, found 255.0763.

**benzo[b]naphtho [*1,2-f*]oxepin-9-amine (3h)**, Yield 25% (0.065 g). mp. 172 °C. **^1^H NMR** (500 MHz, CDCl_3_, 298 K): δ (ppm): 8.09 (d, *J* = 8.5 Hz, 1H), 7.83 − 7.75 (m, 2H), 7.52 (ddd, *J* = 8.4 Hz, *J* = 6.8 Hz, *J* = 1.4 Hz, 1H), 7.43 (ddd, *J* = 8.0 Hz, *J* = 6.8 Hz, *J* = 1.1 Hz, 1H), 7.34 (d, *J* = 8.8 Hz, 1H), 7.29 (d, *J* = 11.4 Hz, 1H), 7.01 (d, *J* = 8.2 Hz, 1H), 6.93 (d, *J* = 11.4 Hz, 1H), 6.57 (d, *J* = 2.3 Hz, 1H), 6.44 (dd, *J* = 8.2 Hz, *J* = 2.3 Hz, 1H), 3.79 (s, 2H). **^13^C NMR** (125 MHz, CDCl_3_, 298 K): δ (ppm): 159.2, 155.9, 148.8, 131.5, 131.3, 131.1, 130.0, 129.9, 128.3, 126.5, 124.9, 124.4, 123.4, 122.9, 121.5, 121.4, 111.6, 107.3 **HRMS** (ESI+) calc. for [M+H]^+^ (C_16_H_14_NO_3_): 259.0997, found 259.0993.

**7-aminodibenzo*[b,f]*oxepin-3-yl acetate (3j),** Yield 38% (0.101 g). mp. 145 °C. **^1^H NMR** (500 MHz, CDCl_3_, 298 K): δ (ppm): 7.09 (d, *J* = 8.4 Hz, 1H), 6.91 (d, *J* = 8.2 Hz, 1H), 6.90 (d, *J* = 2.3 Hz, 1H), 6.84 (dd, *J* = 8.3 Hz, *J* = 2.3 Hz, 1H), 6.55 (d, *J* = 11.3 Hz, 1H), 6.46 – 6.44 (m, 2H), 6.42 (dd, *J* = 8.1 Hz, *J* = 2.3 Hz, 1H). **^13^C NMR** (125 MHz, CDCl_3_, 298 K): δ (ppm): 169.1, 158.1, 157.0, 151.3, 148.7, 130.4, 130.0, 129.3, 129.0, 125.6, 120.8, 118.0, 115.0, 111.6, 107.7, 21.1. **HRMS** (ESI+) calc. for [M+H]^+^ (C_16_H_14_NO_3_): 268.0968, found 268.0964.

### 4.2. NMR Measurements

All the spectra were recorded using a Varian VNMRS spectrometer operating at an 11.7 T magnetic field. Measurements were performed for ca. 1.0M solutions of all the compounds in DMSO-d_6_ or CDCl_3_. The residual signals of DMSO-d_6_ (2.54 ppm) and CDCl_3_ (7.26 ppm) in ^1^H NMR and of the DMSO-d_6_ signal (40.45 ppm) and of CDCl_3_ (77.0 ppm) in ^13^C NMR spectra were used as the chemical shift references. Spin multiplicities are described as s (singlet), d (doublet), t (triplet), q (quartet), m (multiplet), and dd (double doublet). Coupling constants are reported in hertz. All the proton spectra were recorded using the standard spectrometer software and parameters set: acquisition time 3 s, pulse angle 30°. The standard measurement parameter set for ^13^C NMR spectra was: pulse width 7 μs (the 90° pulse width was 12.5 μs), acquisition time 1 s, spectral width 200 ppm, 1000 scans of 32 K data point were accumulated and after zero-filling to 64 K; and the FID signals were subjected to Fourier transformation after applying a 1Hz line broadening.

### 4.3. Biological Evaluation

#### 4.3.1. Cell Culturing

Cells were cultured in a humidified incubator at 37 °C under 5% CO_2_. Human colorectal carcinoma (HCT116) cells were grown in McCoy’s 5A Medium (ATCC, Catalog No. 30-2007), human breast adenocarcinoma (MCF-7) and human dermal fibroblasts (HDF-A) cells were maintained in Dulbecco’s Modified Eagle’s Medium (DMEM, Sigma D5546) supplemented with 2 mM L-glutamine. Human embryonic kidney (HEK293) cell line was cultured in Dulbecco’s Modified Eagle’s Medium (DMEM, Gibco 31966-021). All culture media were supplemented with 1% Penicillin-Streptomycin (Sigma-Aldrich, P4333) and 10% fetal bovine serum (Gibco, 10270-106). Cells were sub-cultured at approximately 80–90% confluency to maintain the culture in the logarithmic growth phase.

#### 4.3.2. MTT Cytotoxicity Assay

Cells were seeded at a 96-well plate at the density of 7 × 10^3^ cells per well and allowed to grow for 24 h. Afterward, the medium was aspirated and fresh medium was added (200 μL) with serial dilutions (100 − 10 μM) of tested compounds or DMSO at corresponding concentrations as a control. Following 48 h of incubation, the medium was replaced with phenol red-free medium (100 μL) containing MTT (3-(4,5-dimethylthiazol-2-yl)-2,5-diphenyl-2H-tetrazolium bromide, 0.5 mg/mL) and replaced in the incubator for 4 h. The formed formazan was dissolved in DMSO (100 μL) followed by incubation of the mixture at 37 °C for 10 min. The absorbance was measured at 540 nm. After blank subtraction, the half maximal effective concentration (EC_50_) was calculated by GraphPad Prism software (GraphPad Software Inc, San Diego, CA, USA).

#### 4.3.3. Immunofluorescence

Cells were seeded on coverslips at a 24-well plate at a density of 5 × 10^4^ cells per well and allowed to grow overnight. Next medium was replaced with a medium containing 60 μM solution of the selected compound and incubated for 24 h. Then, cells were fixed and permeabilized with 100% methanol at −20 °C for 15 min and subsequently washed three times with PBS at room temperature. After 1 h blocking with 3% BSA/PBS at 4 °C, slides were incubated with anti-alpha-tubulin 12G10 primary antibody (Developmental Studies Hybridoma Bank, University of Iowa, Iowa City, IA, USA) (diluted to 2 µg/mL in 3% BSA/PBS) overnight at 4 °C. After 3 × 10 min washing with PBS, slides were incubated with AlexaFluor 555-conjugated secondary antibody (diluted to 50 ng/mL in 3% BSA/PBS) (Thermo Fisher Scientific, Waltham, MA, USA, A31570) for 1 h at room temperature. After washing (3 × 10 min with PBS), slides were mounted in Fluoromount-G (Southern Biotech., Birmingham, AL, USA). Cells were recorded using Leica TCS SP8 (Leica Microsystems, Wetzlar, Germany) confocal microscope.

### 4.4. Computational Aspects

The optimum ground-state geometry for selected compounds **1a**, **1c**, **1d**, **1i**, **2i**, **2j**, and **3h** was calculated using density functional theory (DFT) [54]. The B3LYP functional and 6-311++g (2d,p) basis set and the continuum model (PCM; Gaussian 03W) [55] were used to simulate the effects of the solvent -DMSO. All calculations were performed on a server equipped with a 16 quad-core XEON (R) CPU E7310 processor operating at 1.60 GHz. The operating system was Open SUSE 10.3.

Selected compounds were docked into the 3D X-ray structure of αβ-tubulin heterodimer (PDB code: 1SA0) [59] using the Auto-Dock Vina software (the Broyden-Fletcher-Goldfarb-Shanno (BFGS) method) [60]. The PyMOL graphical user interface and python scripts provided by AutodockTools [61] were employed to set up the αβ-tubulin structure: chains C, D, and E were removed as well as small molecules (magnesium ion, GDT, GTP, and DAMA-colchicine), and all hydrogens were added. Next python script prepare_receptor4.py was used for .pdbqt file generation. The 3D structures of ligand molecules were built, optimized (B3LYP functional and 6-311G 6-311++g (2d,p) basis set level), and saved in .pdb format. The python script *prepare_ligand4.py* was employed to set up the ligand and the pdbqt file was saved. Auto-Dock Vina software was employed for all docking calculations. The AutoDockTools program was used to generate the docking input files. In docking, a grid box size of 56 × 56 × 56 points in x, y, and z directions was built, and the maps were center located (39.82, 53.24, −8.21) in the binding site of the protein. A grid spacing of 0.375 Å (approximately one-fourth of the length of a carbon-carbon covalent bond) was used for the calculation of the energetic map. All computations were performed on an Intel^®^ Core^TM^ and 7-4702MQ 3.2 GHz processor running Ubuntu 18.04 Workstation Linux distribution. PyMOL software (www.pymol.org/ (accessed on 1 January 2021)) was used to analyze the docking results [62]. The Protein-Ligand Interaction Profiler (PLIP) was used to predict protein-docked ligand interactions (the default threshold was used for detection steps: 4.0 Å max. distance of carbon atoms for a hydrophobic interaction; 4.1 Å max. distance between acceptor and donor in hydrogen bonds) [63].

## Figures and Tables

**Table 1:** Half-maximal inhibitory concentration (IC50) and selectivity index (SI) calculated for tested compounds. Data are presented as the mean ± standard error of the mean (SEM).

Compound	HEK293	HDF-A	HCT116	SI_1_	SI_2_	MCF-7	SI_3_	SI_4_
	IC_50_ (μM)	IC_50_ (μM)	IC_50_ (μM)			IC_50_ (μM)		
**1a**	18 ± 1	38 ± 1	23 ± 1	0.8	0.8	36 ± 2	0.5	1.1
**1c**	34 ± 4	40 ± 4	38 ± 5	0.9	1.1	43 ± 2	0.7	1.0
**1d**	33 ± 2	40 ± 2	18 ± 3	1.8	2.2	47 ± 1	0.7	0.9
**1i**	54 ± 2	41 ± 2	52 ± 2	1.0	0.8	69 ± 3	0.8	0.6
**2i**	88 ± 5	93 ± 7	55 ± 4	1.6	1.7	67 ± 6	1.2	1.4
**2j**	85 ± 4	ND	53 ± 3	1.6	ND	36 ± 3	2.4	ND
**3h**	48 ± 3	127 *	66 ± 2	0.7	1.9 *	ND	ND	ND

SI_1_ = IC_50_(HEK-293)/IC_50_(HCT116); SI_2_ = IC_50_(HDF-A)/IC_50_(HCT116); SI_3_ = IC_50_(HEK-293)/IC_50_(MCF-7); SI_4_ = IC_50_(HDF-A)/IC_50_(MCF-7); “*”—The predicted IC_50_ (above the maximum concentration tested—100 μM); “ND”—the IC_50_ could not be calculated.

## Data Availability

The data presented in this study are available in Supplementary Materials.

## Supplementary Materials

The following supporting information can be downloaded at: https://www.mdpi.com/article/10.3390/molecules28083558/s1. Figures S1–S54. NMR spectra of obtained compounds. Tables S1–S7. The calculated coordinates of compounds (**1a**, **1c**, **1d**, **1i**, **2i**, **2j** and **3h**). Figures S55–S61. Visualization of calculated geometry of compounds (**1a**, **1c**, **1d**, **1i**, **2i**, **2j**, **3h**). Table S8. Estimated binding free energy, visualization of binding pose and predicted interactions with αβ-tubulin heterodimer for compounds (**1a**, **1c**, **1d**, **1i**, **2i**, **2j**, **3h**).

## Sample Availability

Samples of the compounds are available from the authors.
