# Supplementary material for: Systematic Studies on Anti-Cancer Evaluation of Stilbene and Dibenzo[b,f]oxepine Derivatives
Source: Molecules. 2023 Apr 18;28(8):3558. doi: 10.3390/molecules28083558 (PMC10146957; doi:10.3390/molecules28083558)
Supplement: Supplementary file 1 [file molecules-28-03558-s001.zip › molecules-2295761-supplementary.pdf]

Systematic studies on anti-cancer evaluation of stilbene and dibenzo[*b*,*f*]oxepine derivatives

Supplementary Materials

Figure S1-S54. NMR spectra of obtained compounds

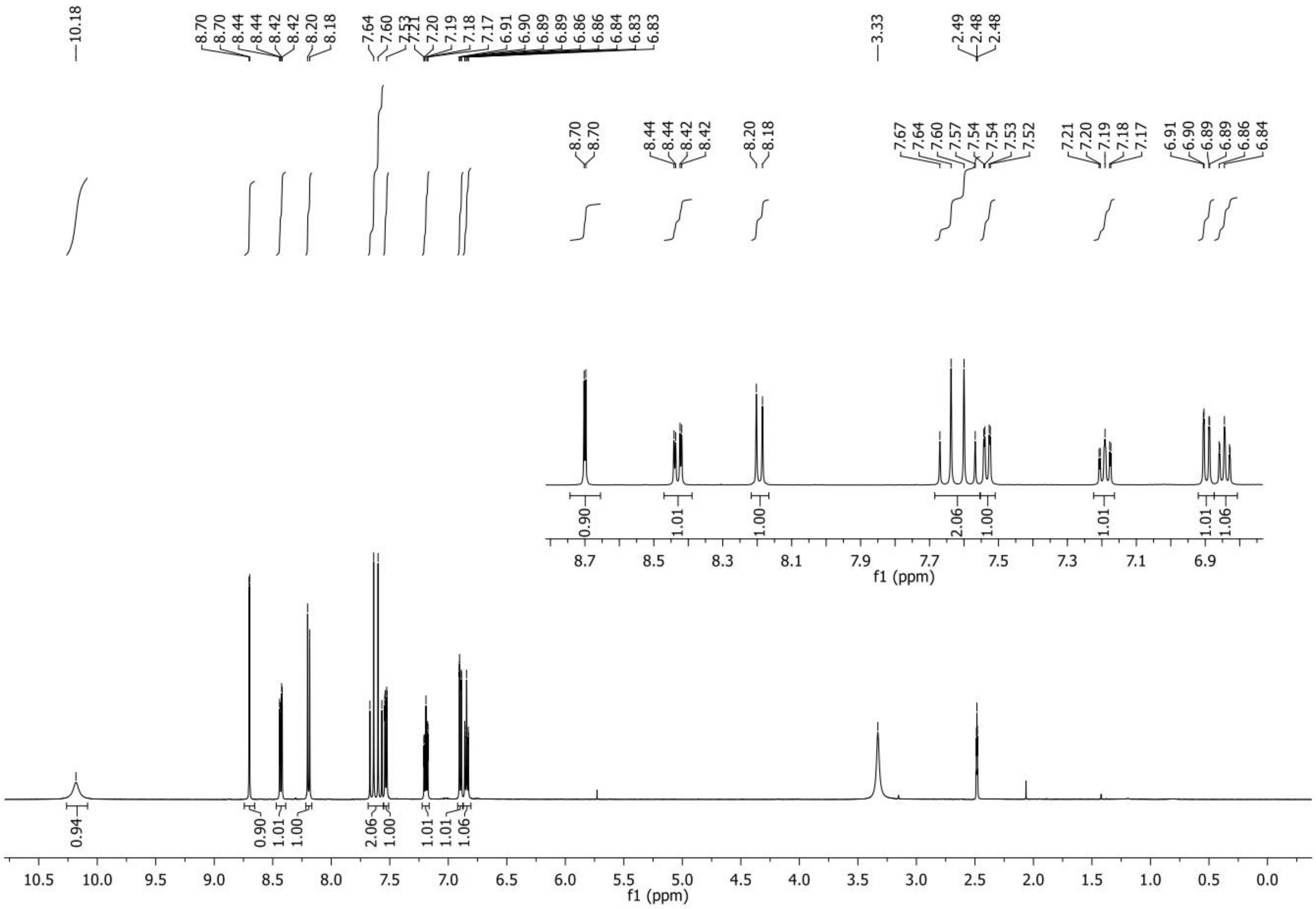

Figure S1. The ^{1}H NMR of (*E*)-2-hydroxy-2',4'-dinitrostilbene (**1a**).

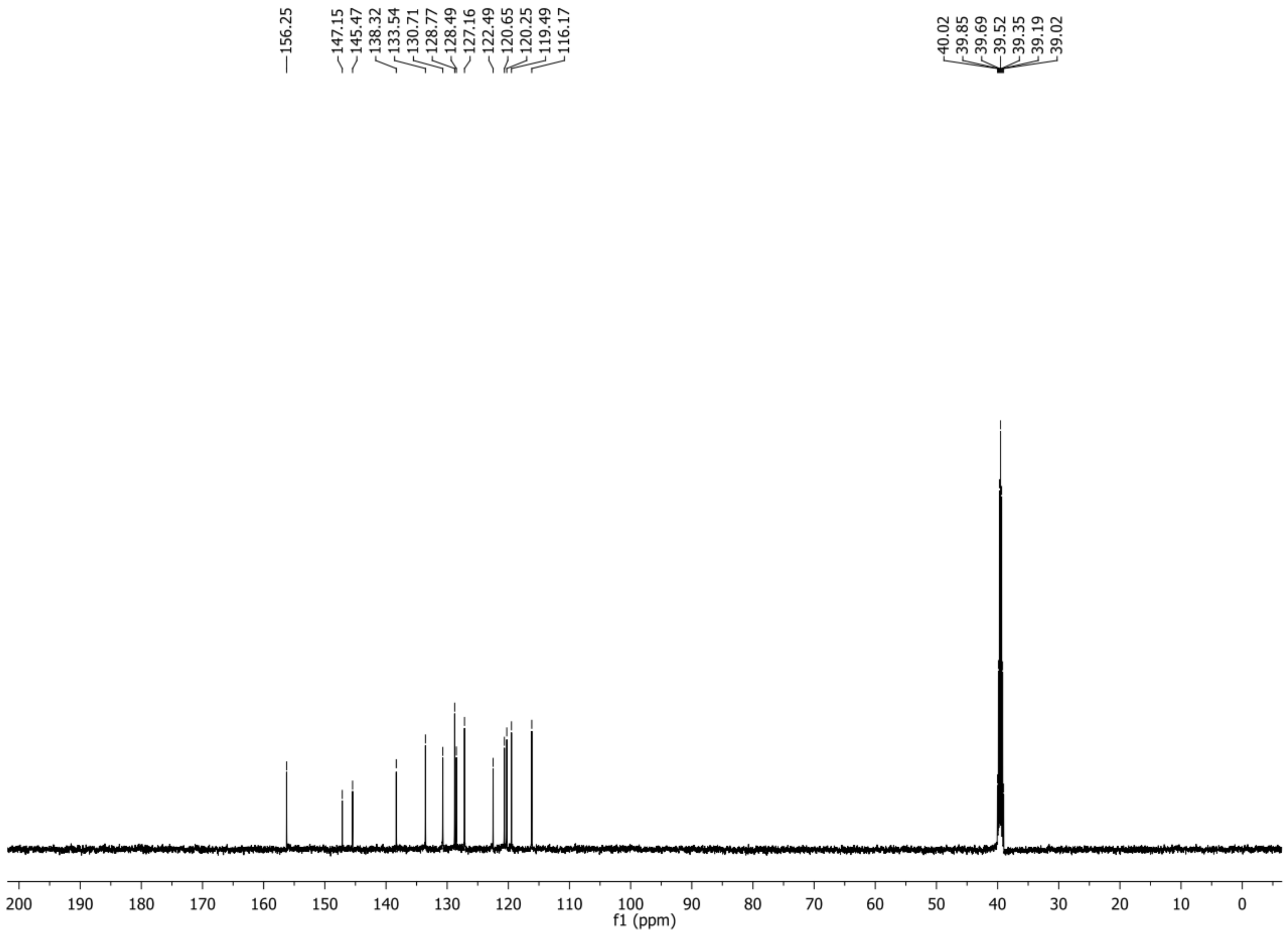

Figure S2. The ^{13}C NMR of (*E*)-2-hydroxy-2',4'-dinitrostilbene (**1a**).

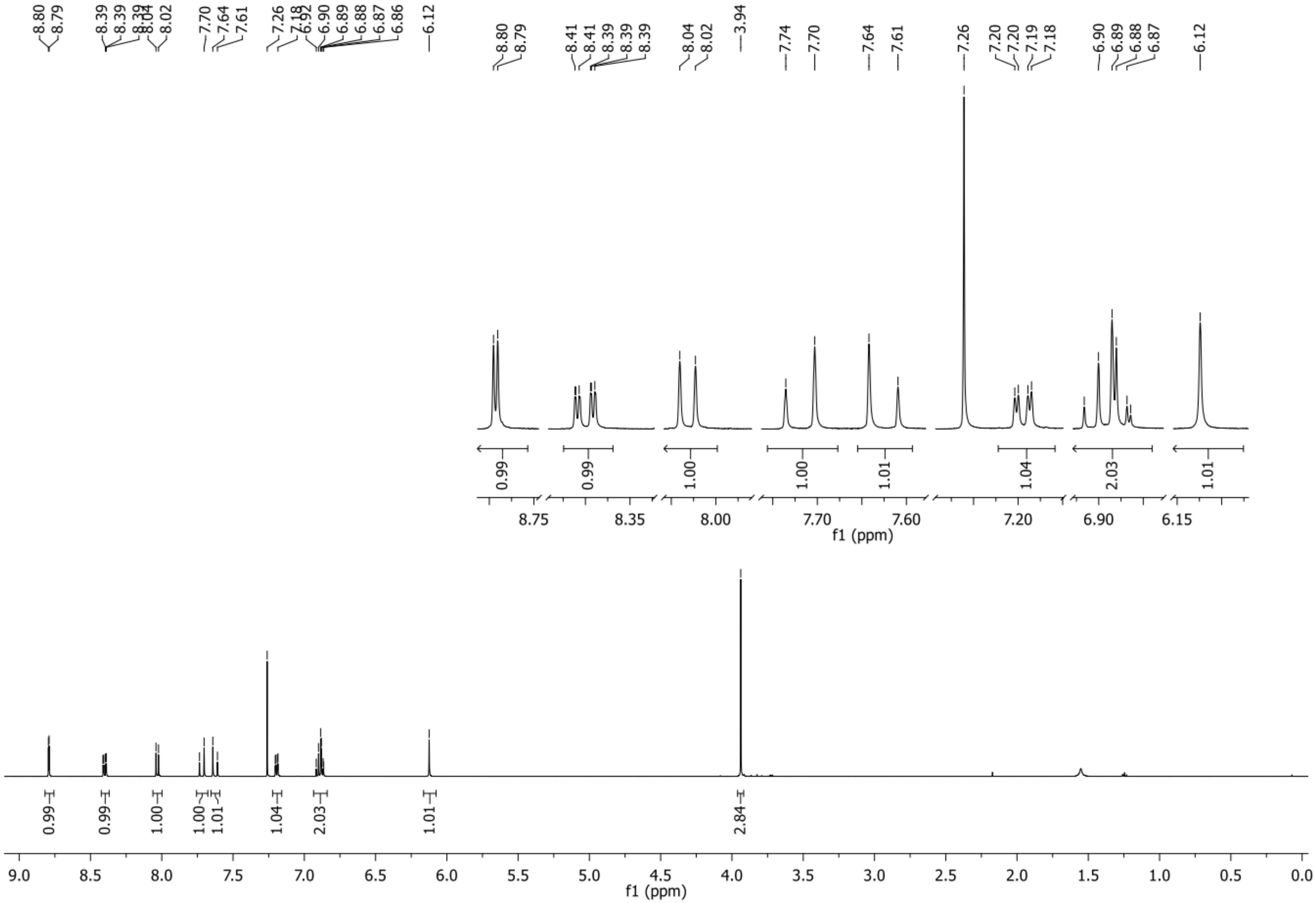

Figure S3. The ^{1}H NMR of (*E*)- 2-hydroxy-3-methoxy-2',4'-dinitrostilbene (**1b**).

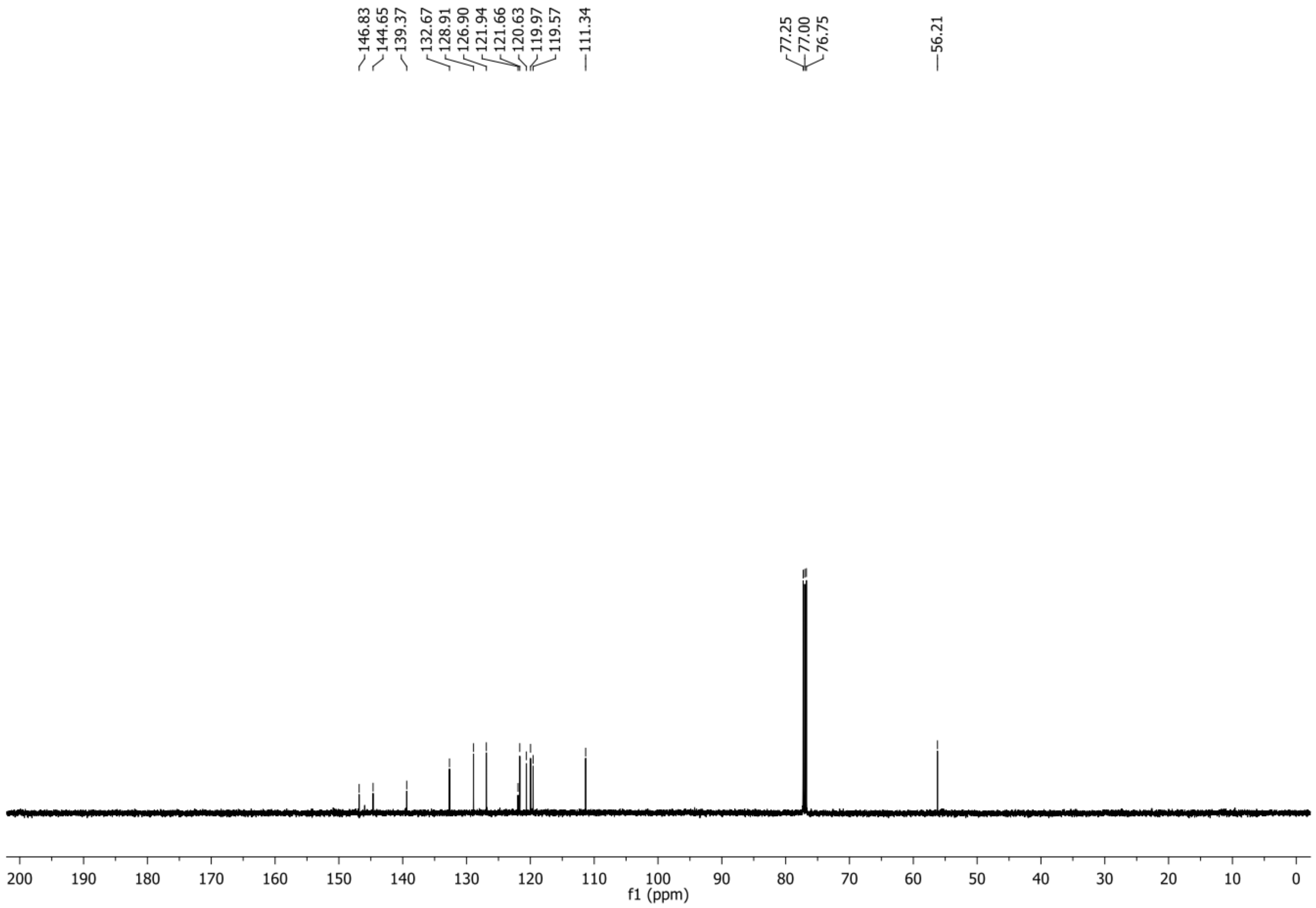

Figure S4. The ^{13}C NMR of (*E*)- 2-hydroxy-3-methoxy-2',4'-dinitrostilbene (**1b**).

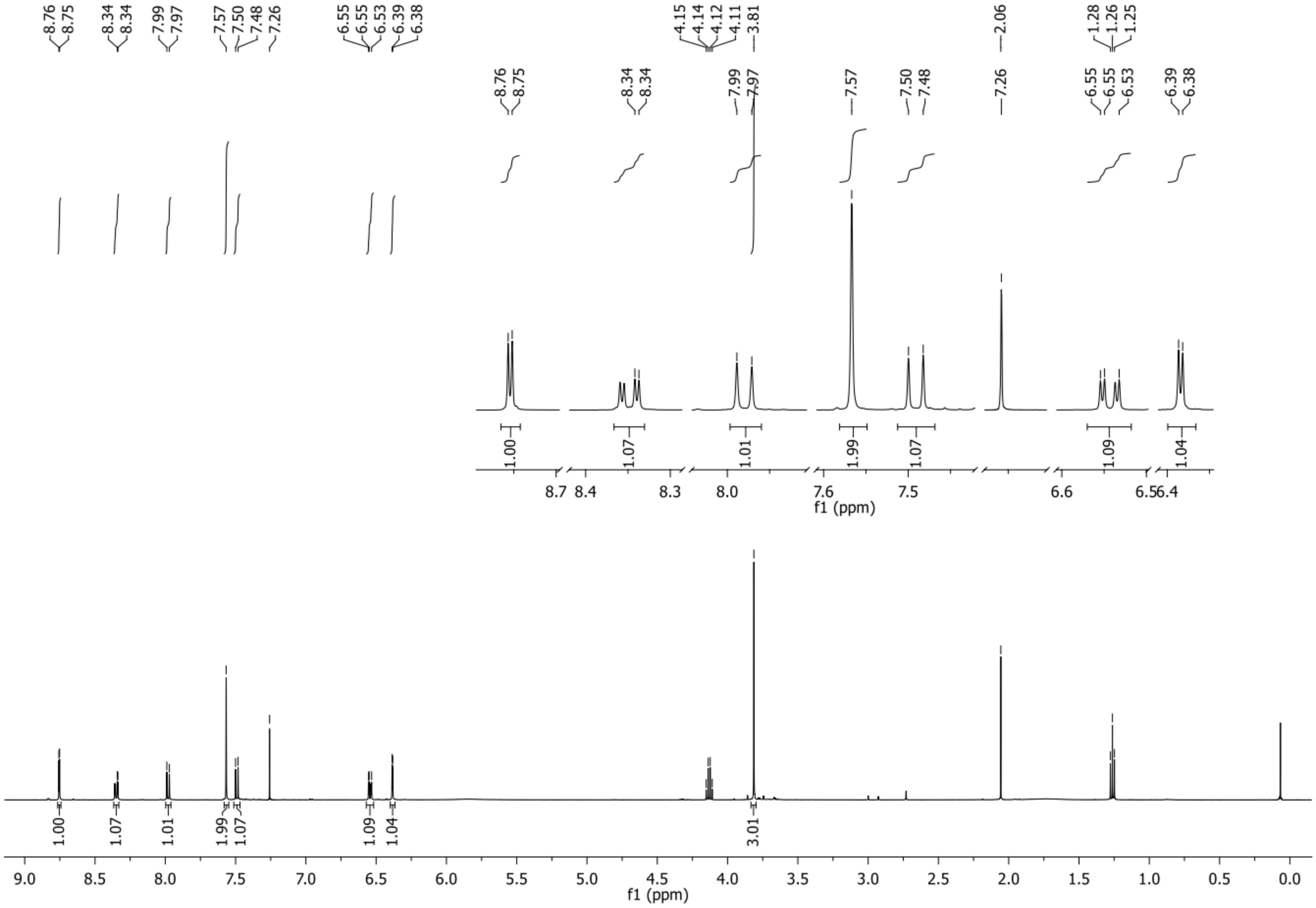

Figure S5. The ^{1}H NMR of (*E*)-2-hydroxy-4-methoxy-2',4'-dinitrostilbene (**1c**).

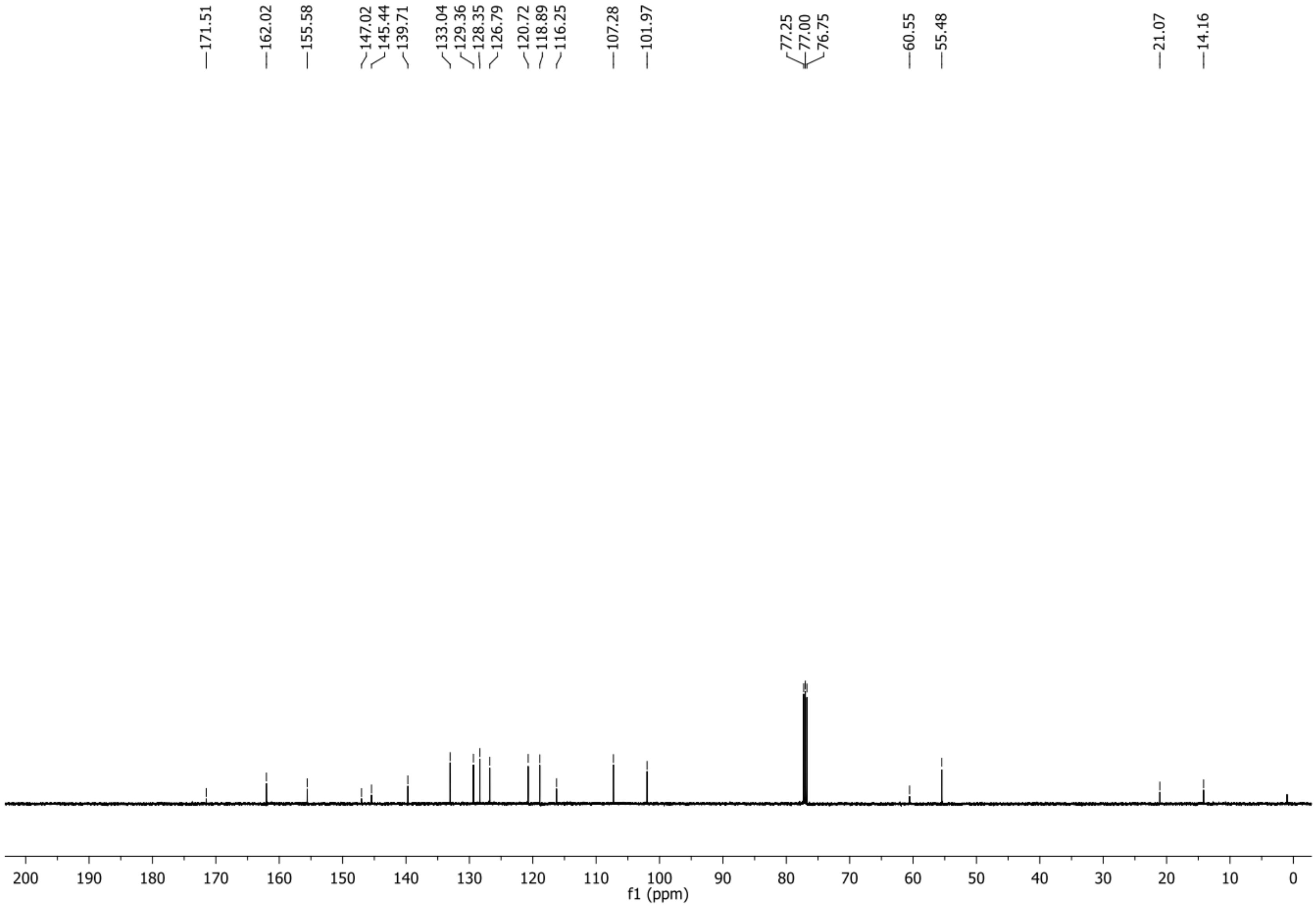

Figure S6. The ^{13}C NMR of (*E*)-2-hydroxy-4-methoxy-2',4'-dinitrostilbene (**1c**).

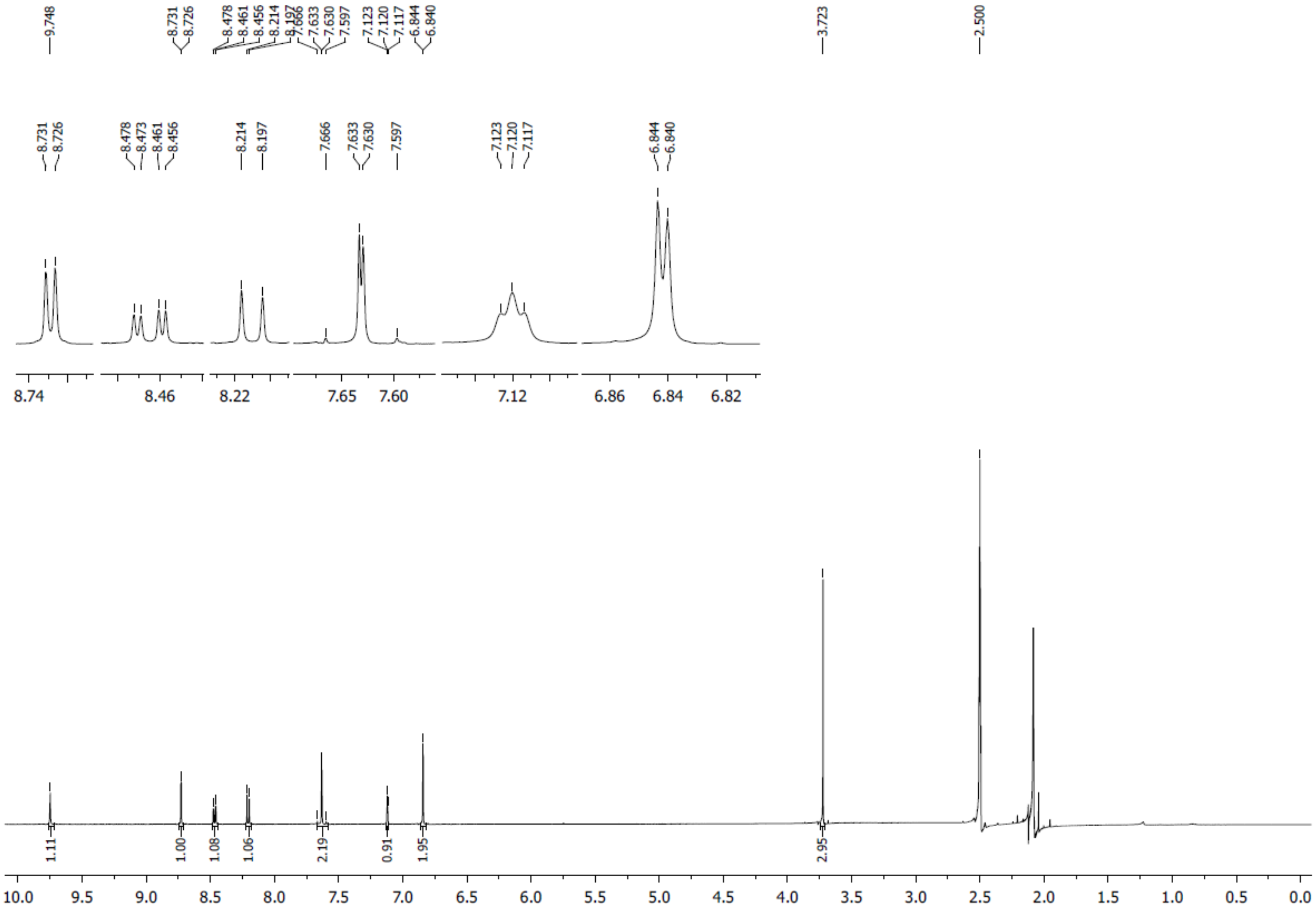

Figure S7. The ^{1}H NMR of (*E*)- 2-hydroxy-5-methoxy-2',4'-dinitrostilbene (**1d**).

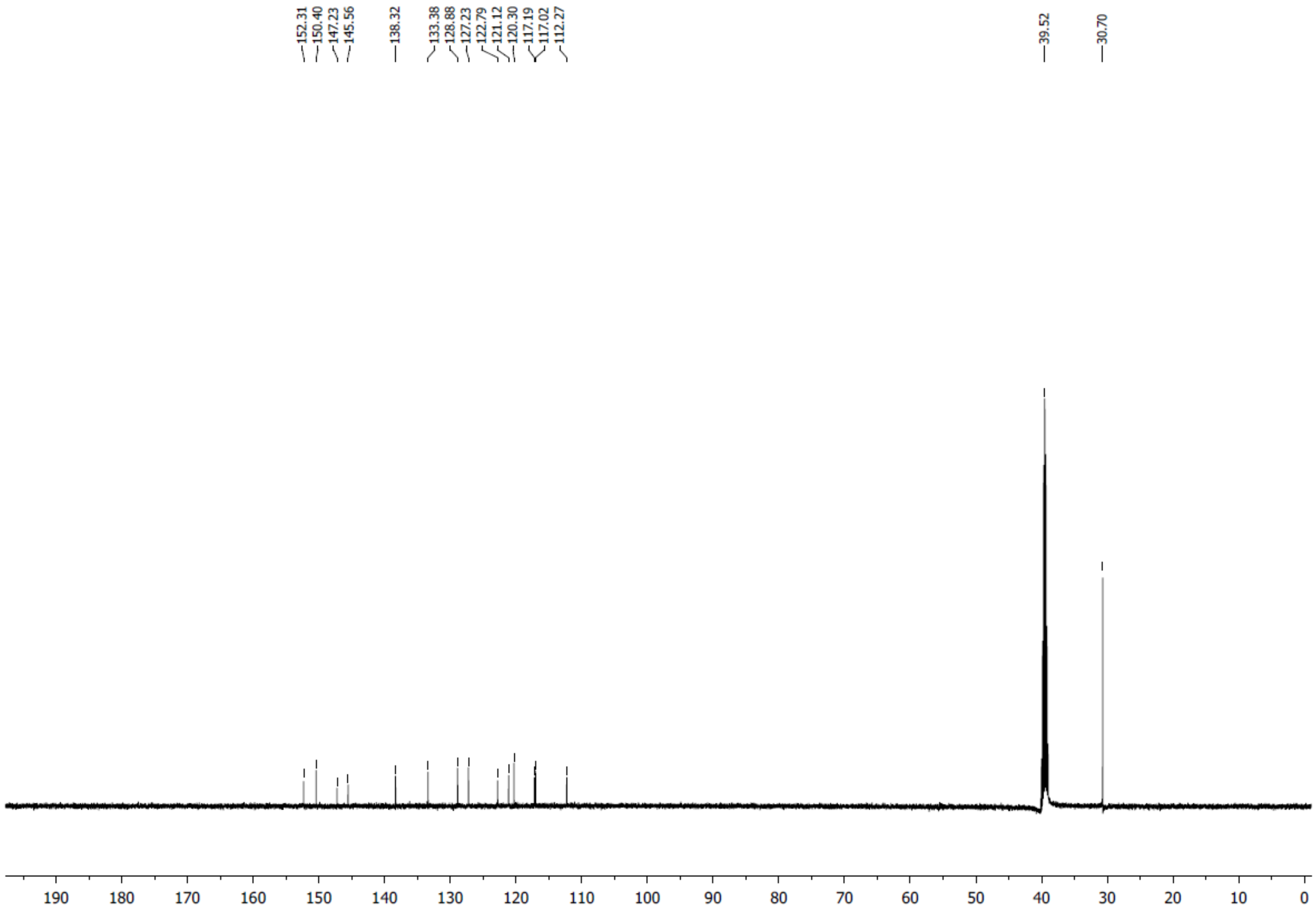

Figure S8. The ^{13}C NMR of (*E*)- 2-hydroxy-5-methoxy-2',4'-dinitrostilbene (**1d**).

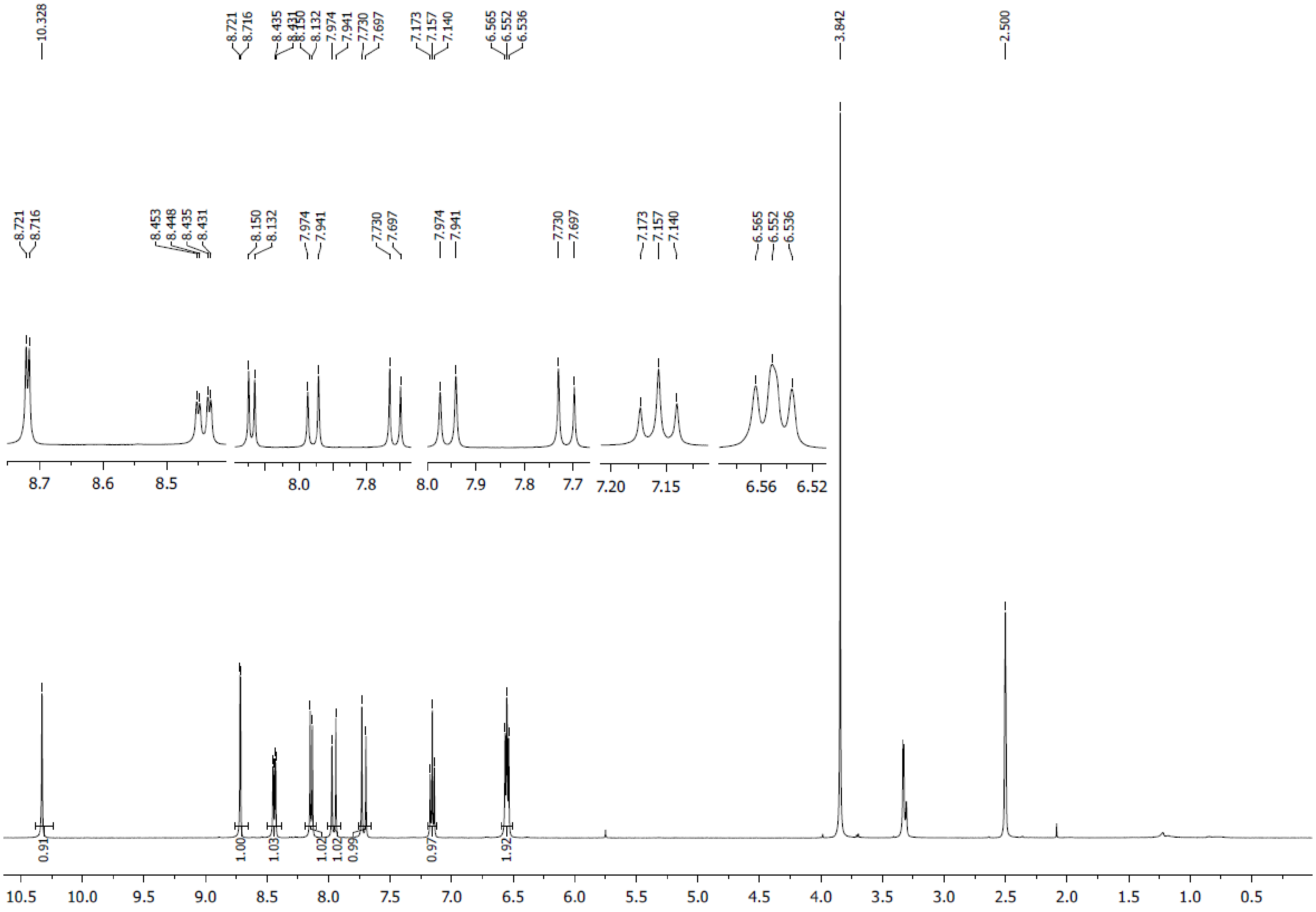

Figure S9. The ^{1}H NMR of (*E*)-2-hydroxy-6-methoxy-2',4'-dinitrostilbene (**1e**).

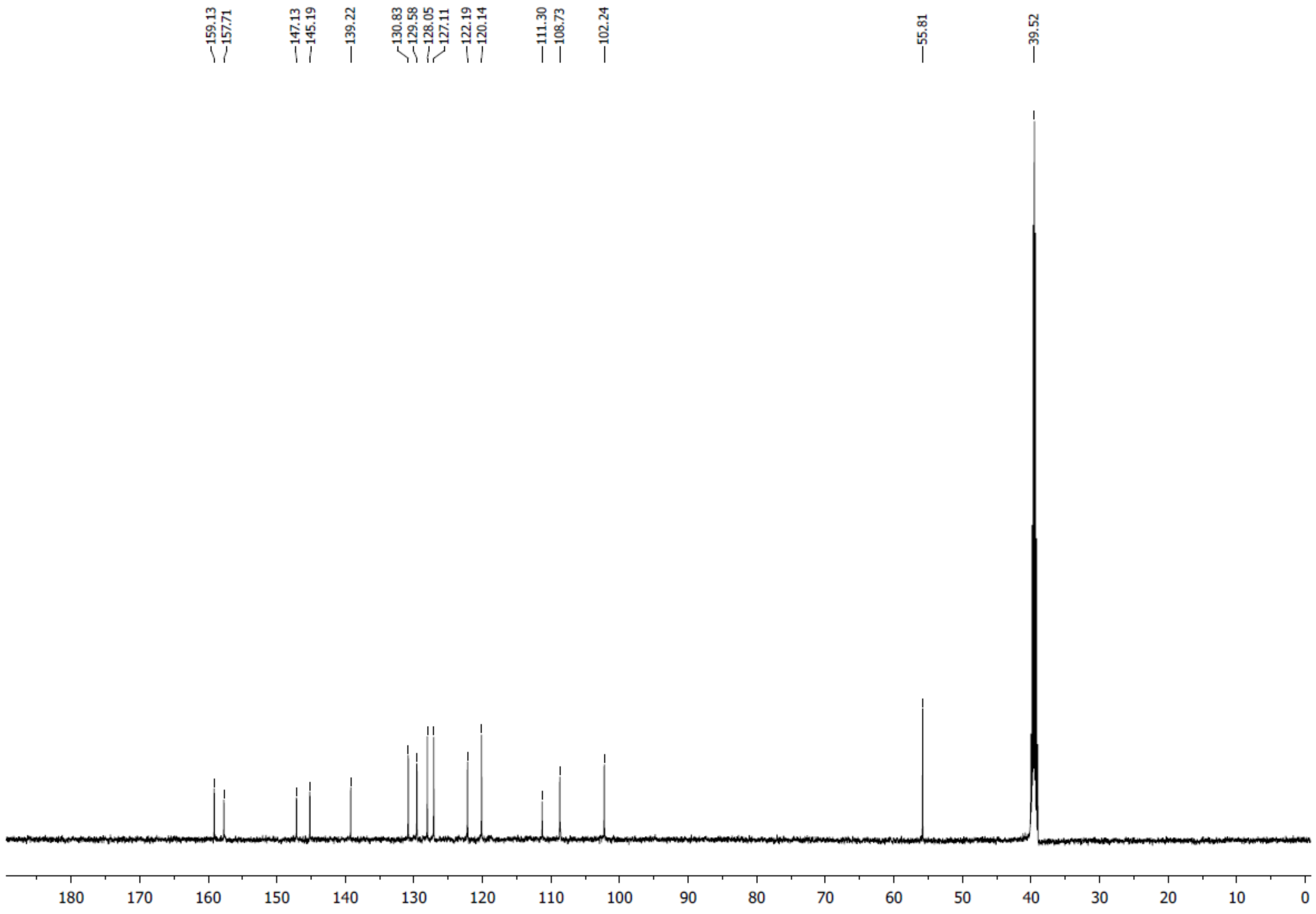

Figure S10. The ^{13}C NMR of (*E*)-2-hydroxy-6-methoxy-2',4'-dinitrostilbene (**1e**).

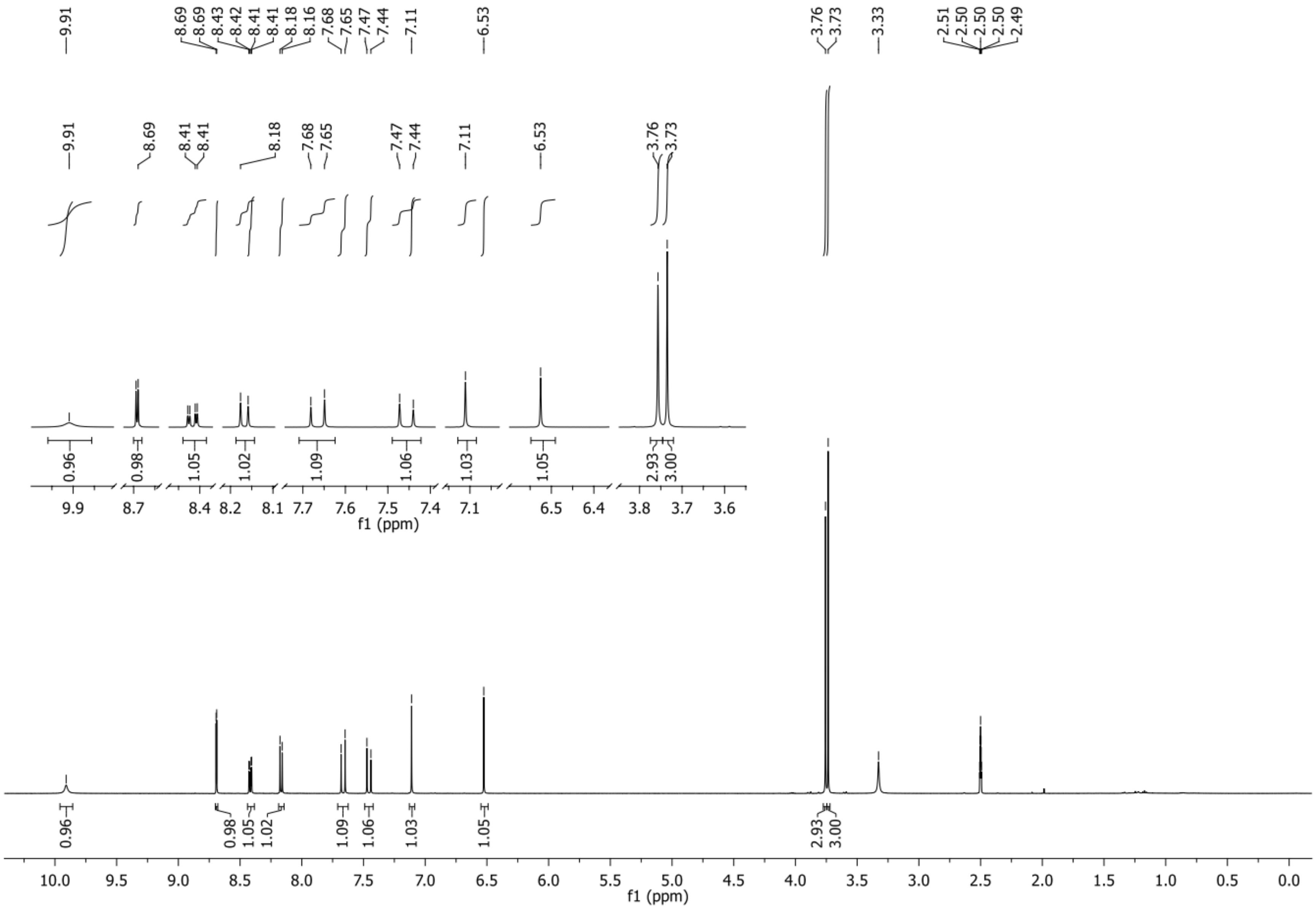

Figure S11. The ^{1}H NMR of (*E*)- 2-hydroxy-4,5-dimethoxy-2',4'-dinitrostilbene (**1f**).

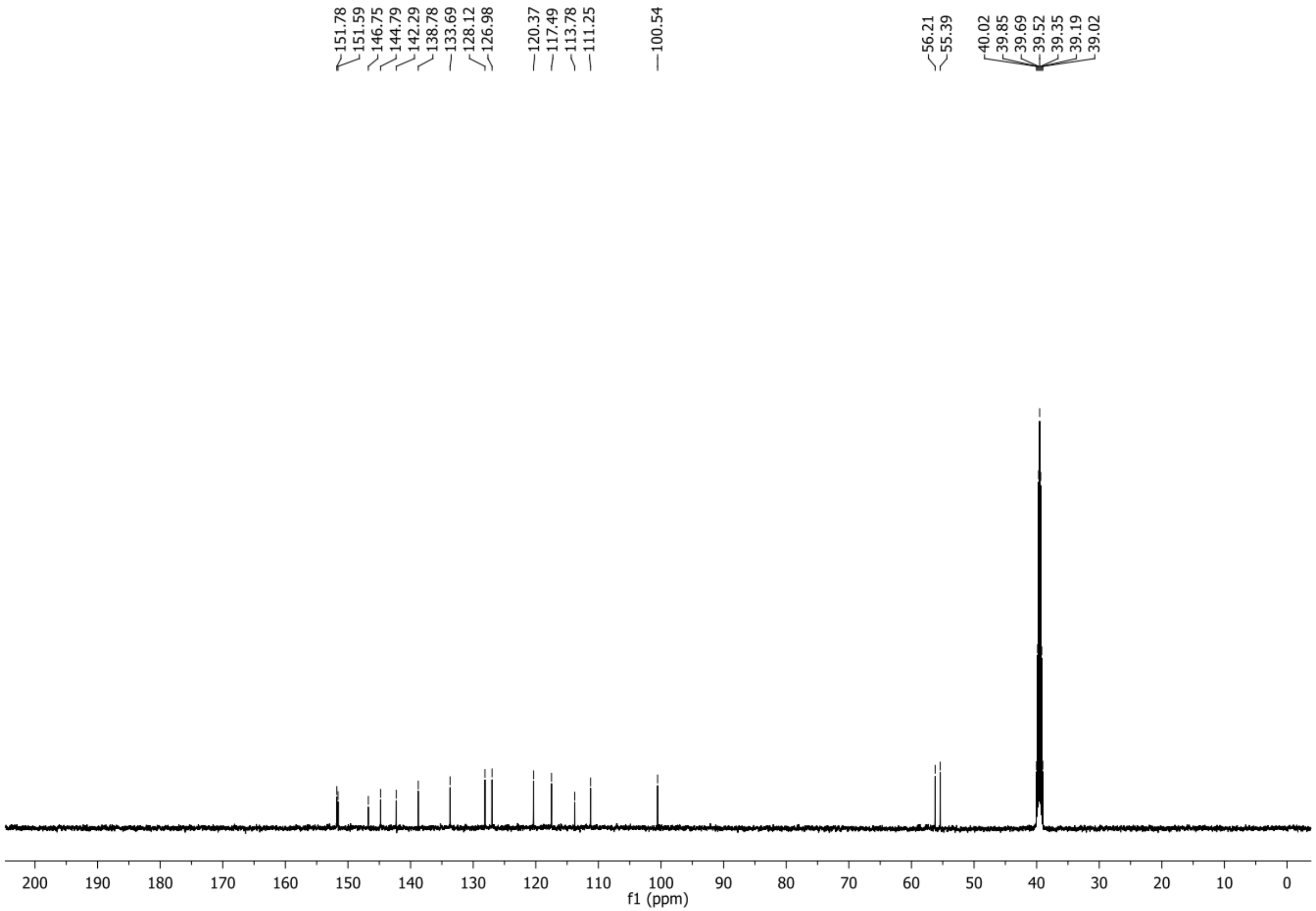

Figure S12. The ^{13}C NMR of (*E*)- 2-hydroxy-4,5-dimethoxy-2',4'-dinitrostilbene (**1f**).

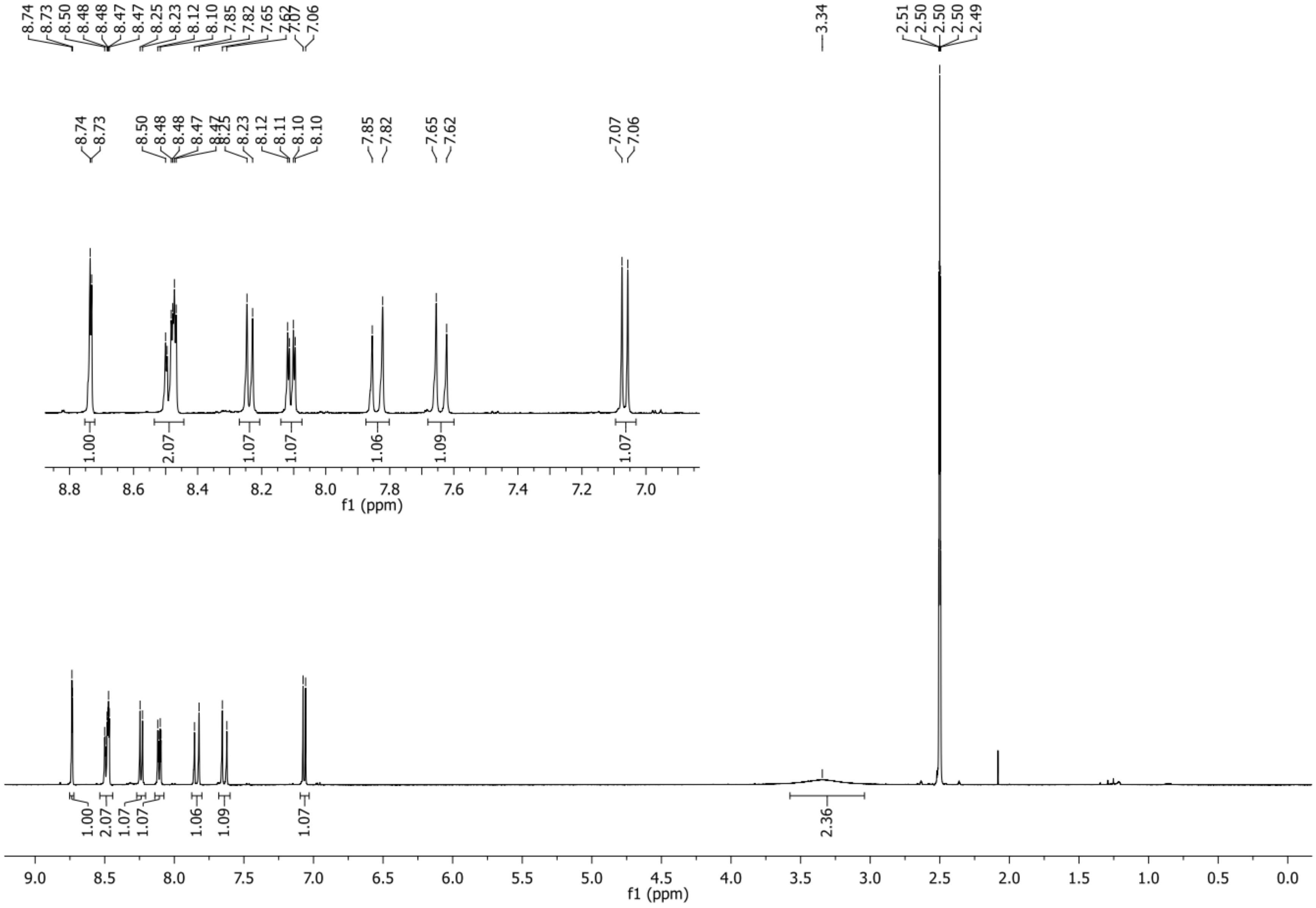

Figure S13. The ^{1}H NMR of (*E*)-2-hydroxy-5,2',4' trinitrostilbene (**1g**).

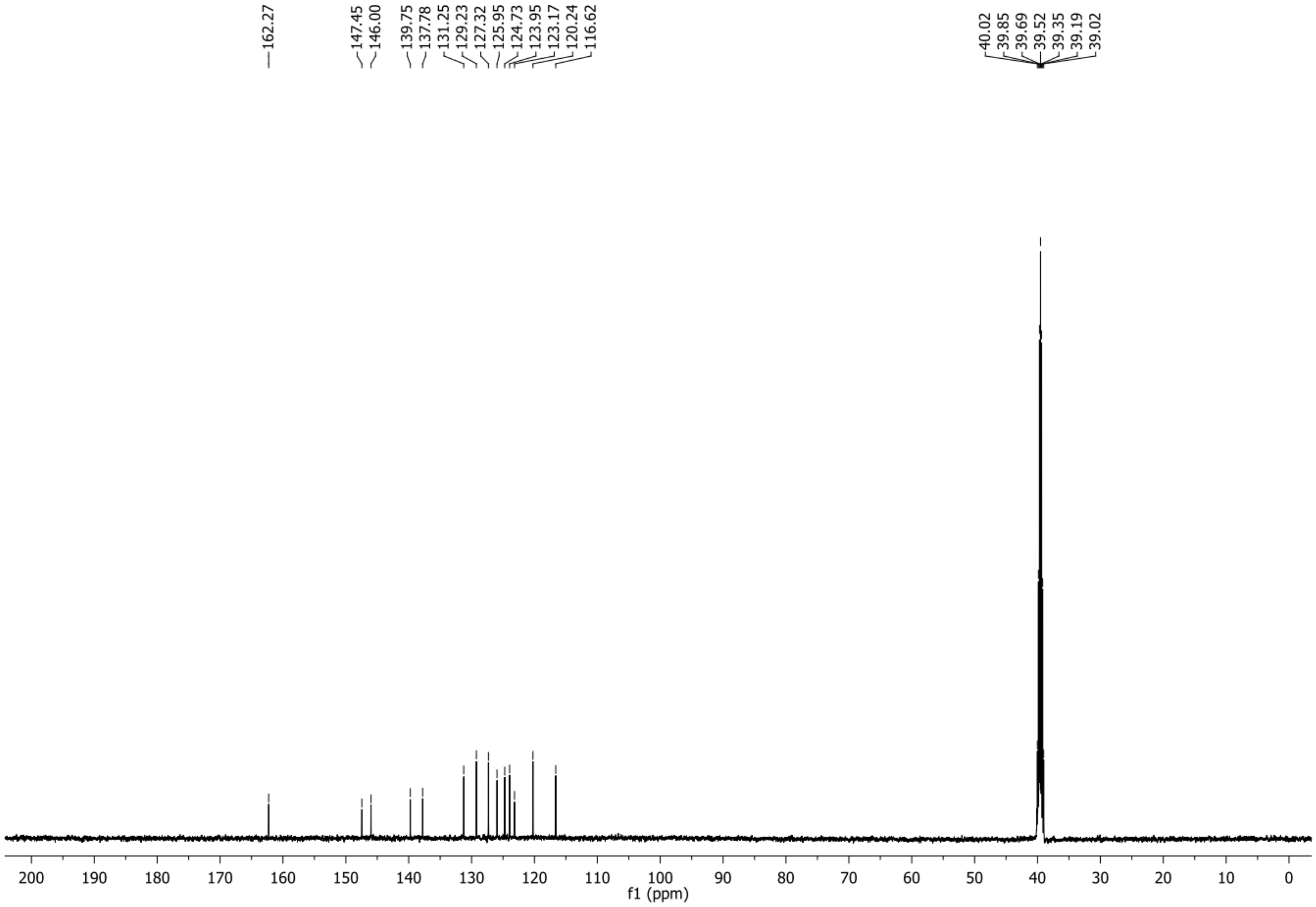

Figure S14. The ^{13}C NMR of (*E*)-2-hydroxy-5,2',4' trinitrostilbene (**1g**).

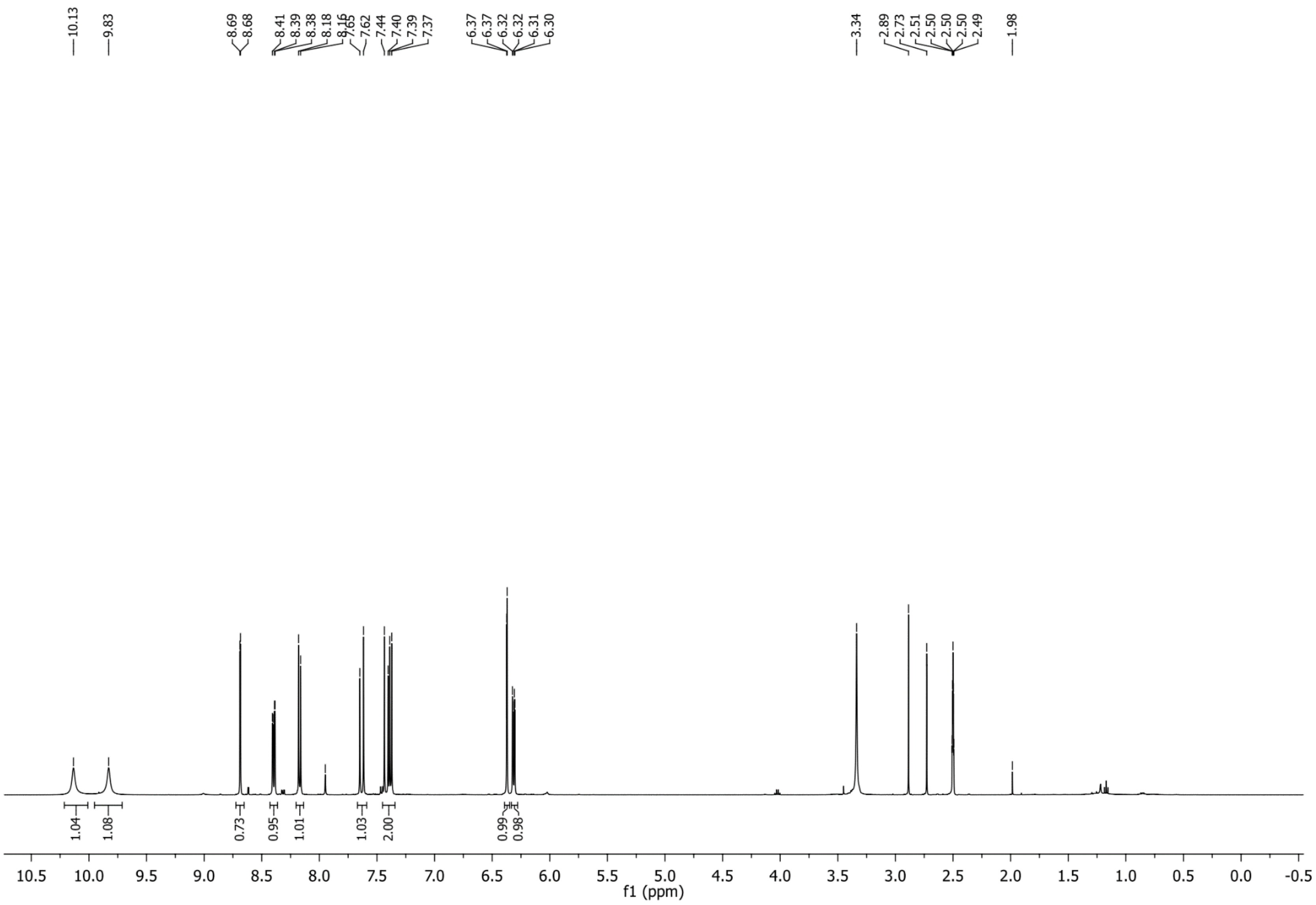

Figure S15. The ^{1}H NMR of (*E*)- 2,4-dihydroxy-2',4'-dinitrostilbene (**1i**).

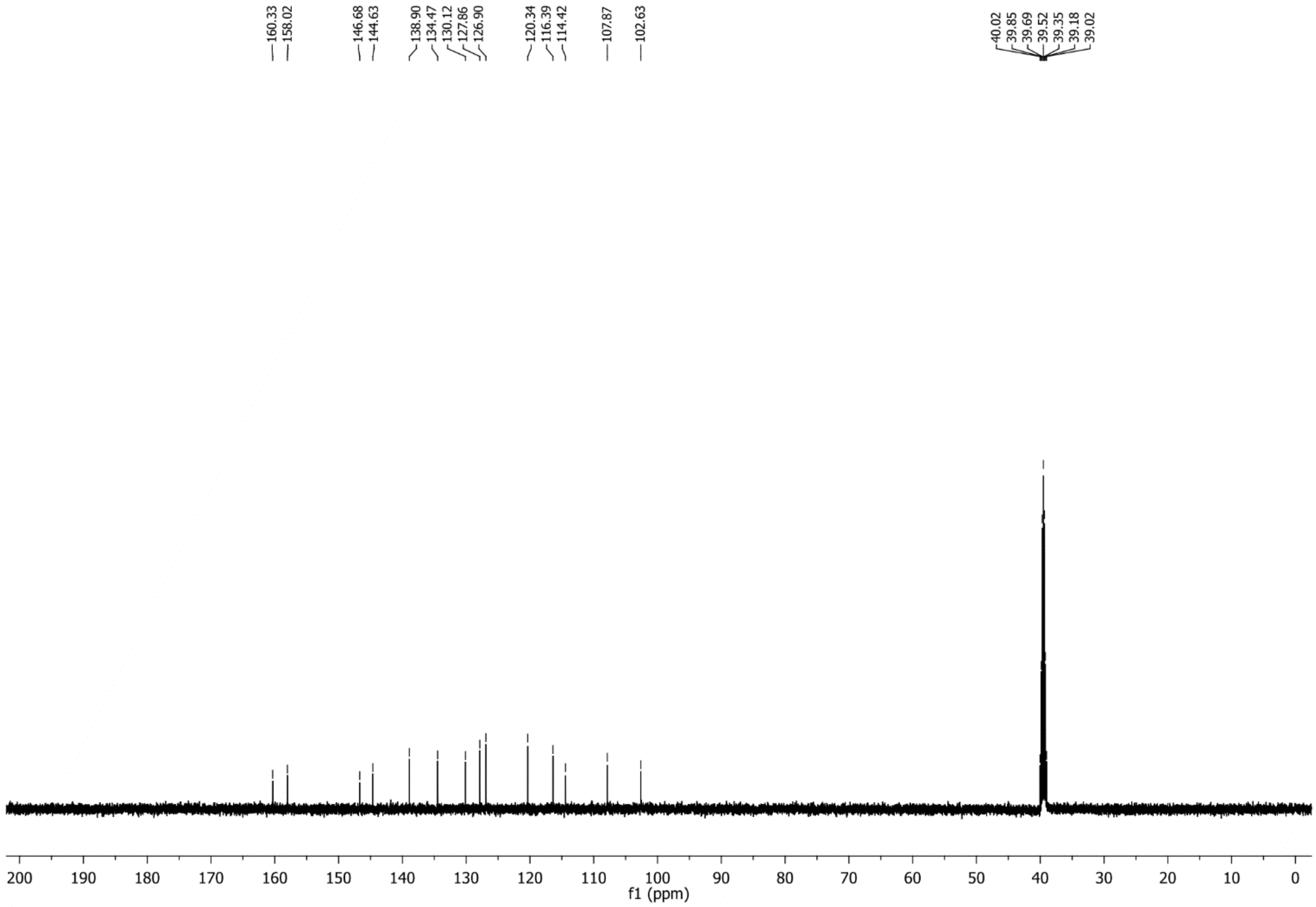

Figure S16. The ^{13}C NMR of (*E*)- 2,4-dihydroxy-2',4'-dinitrostilbene (**1i**).

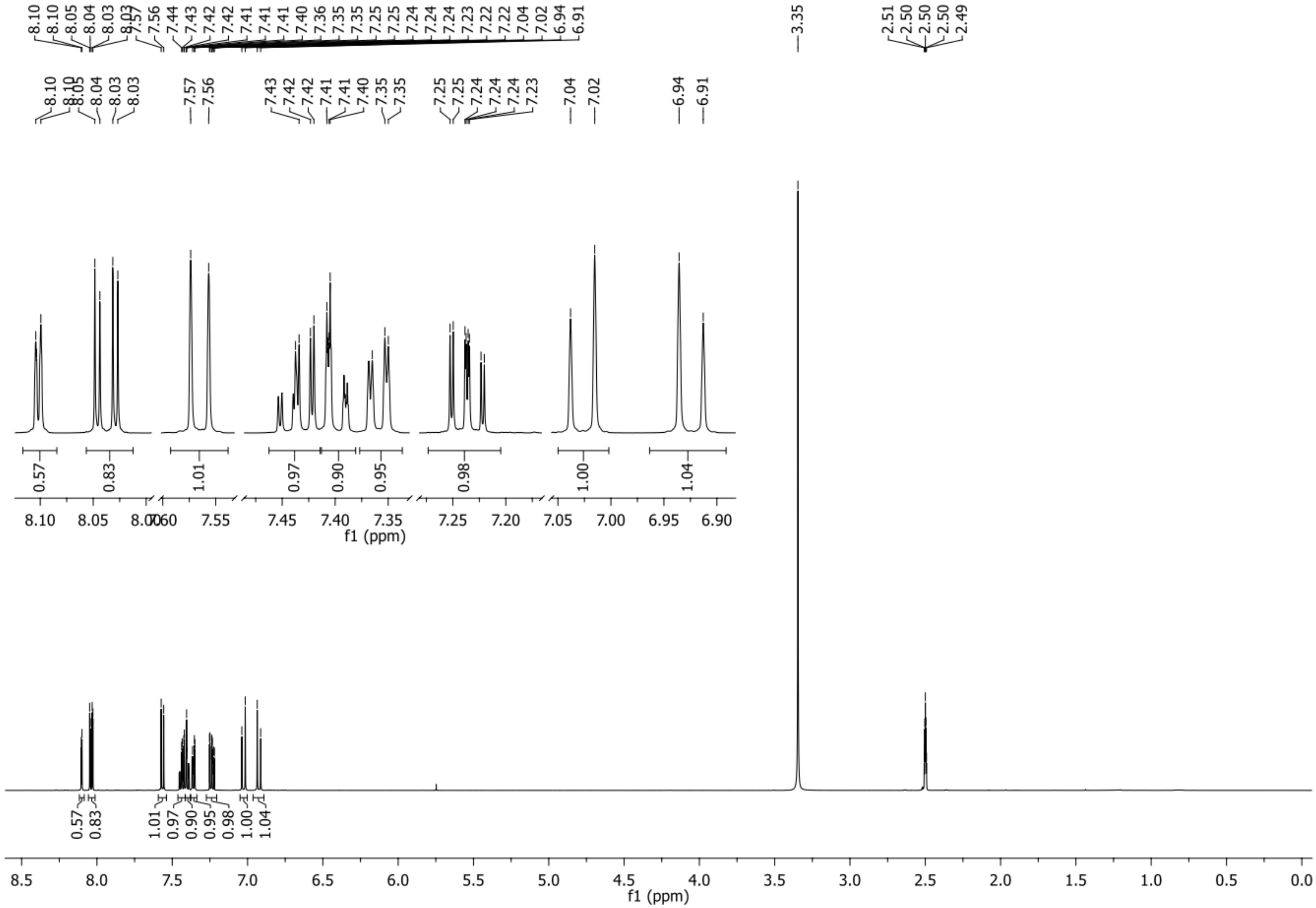

Figure S17. The ^{1}H NMR of 3-nitrodibenzo[*b,f*]oxepine (**2a**).

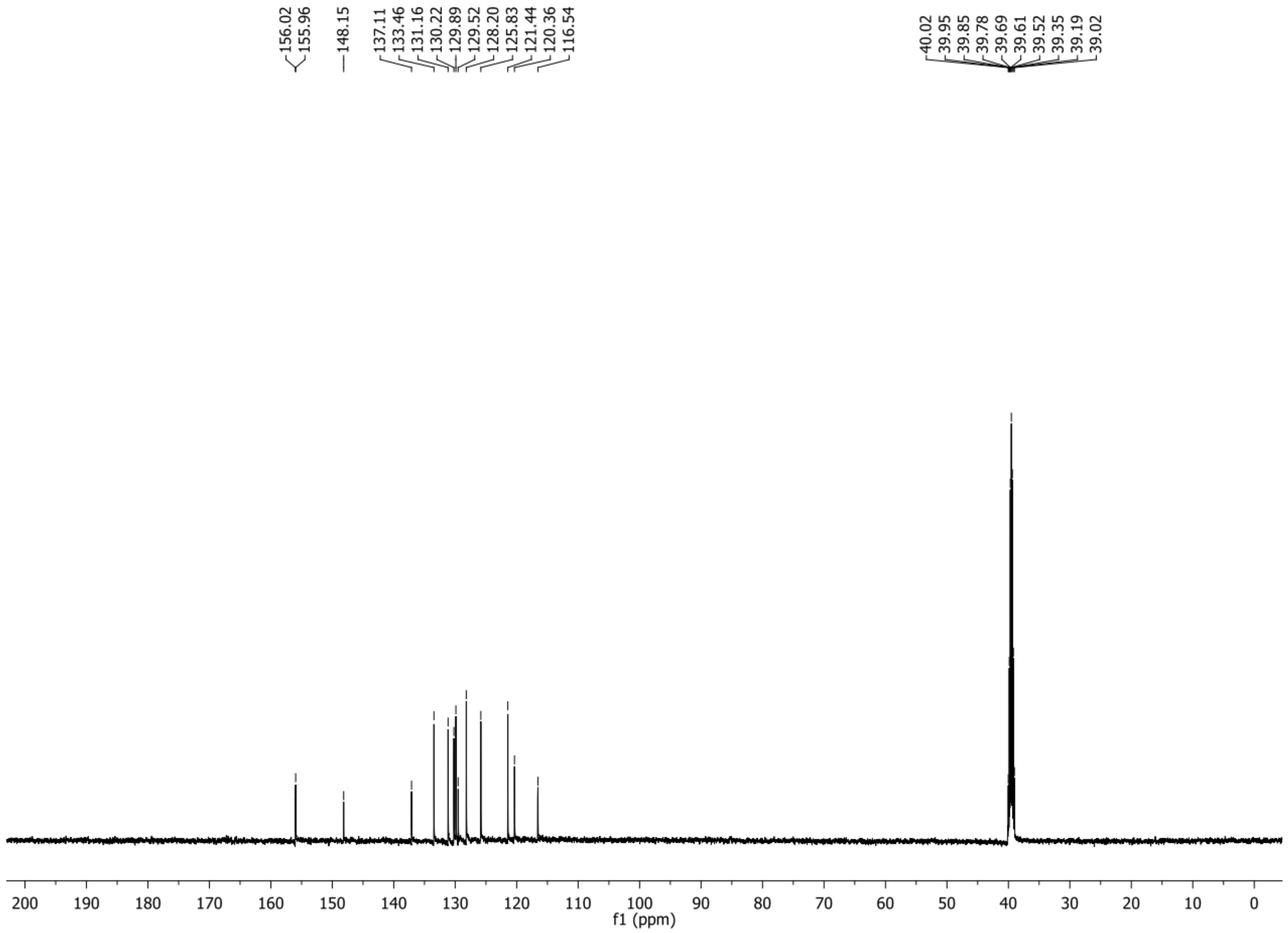

Figure S18. The ^{13}C NMR of 3-nitrodibenzo[*b,f*]oxepine (**2a**).

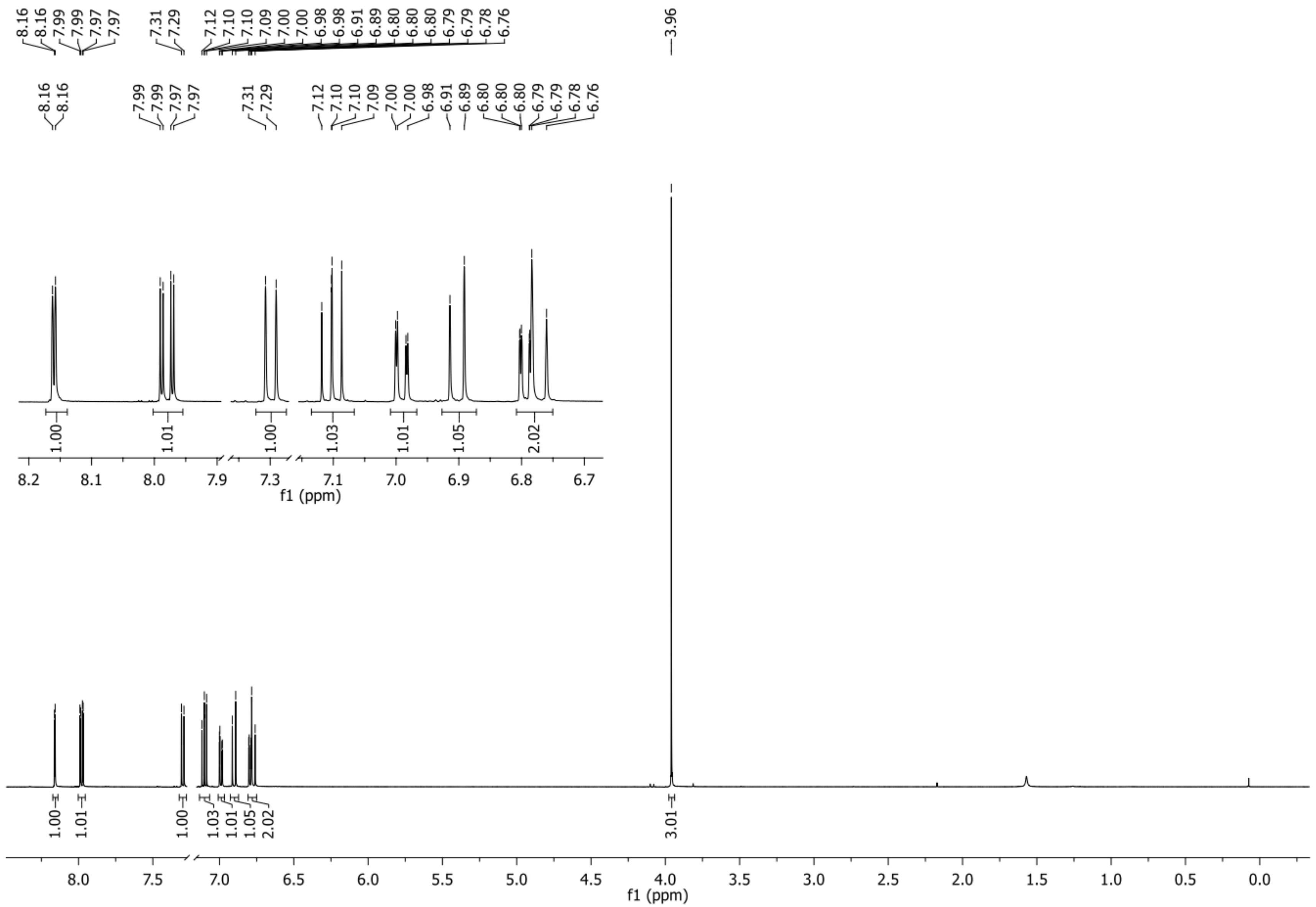

Figure S19. The ^{1}H NMR of 6-methoxy-3-nitrodibenzo[*b*,*f*]oxepine (**2b**).

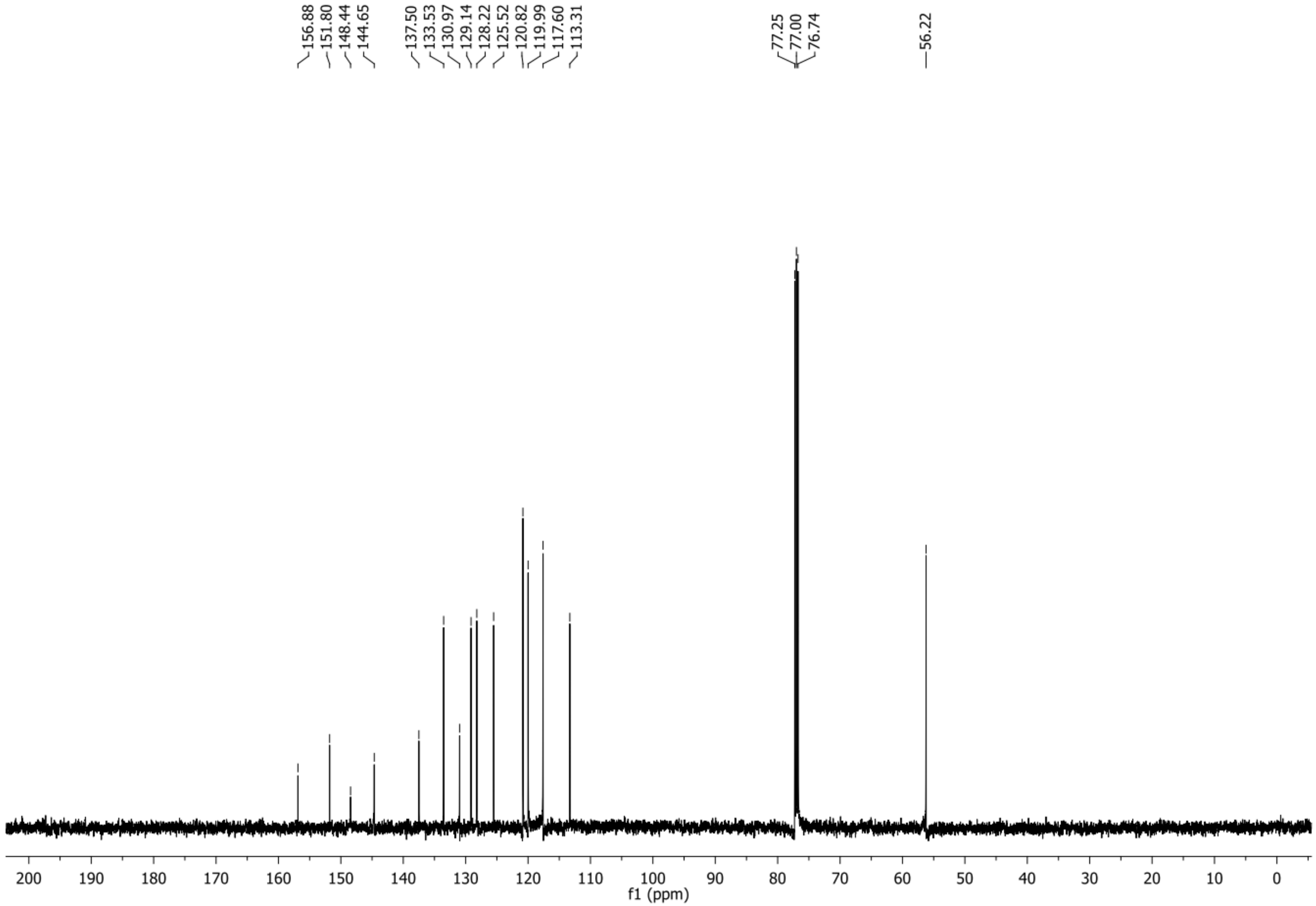

Figure S20. The ^{13}C NMR of 6-methoxy-3-nitrodibenzo[*b,f*]oxepine (**2b**).

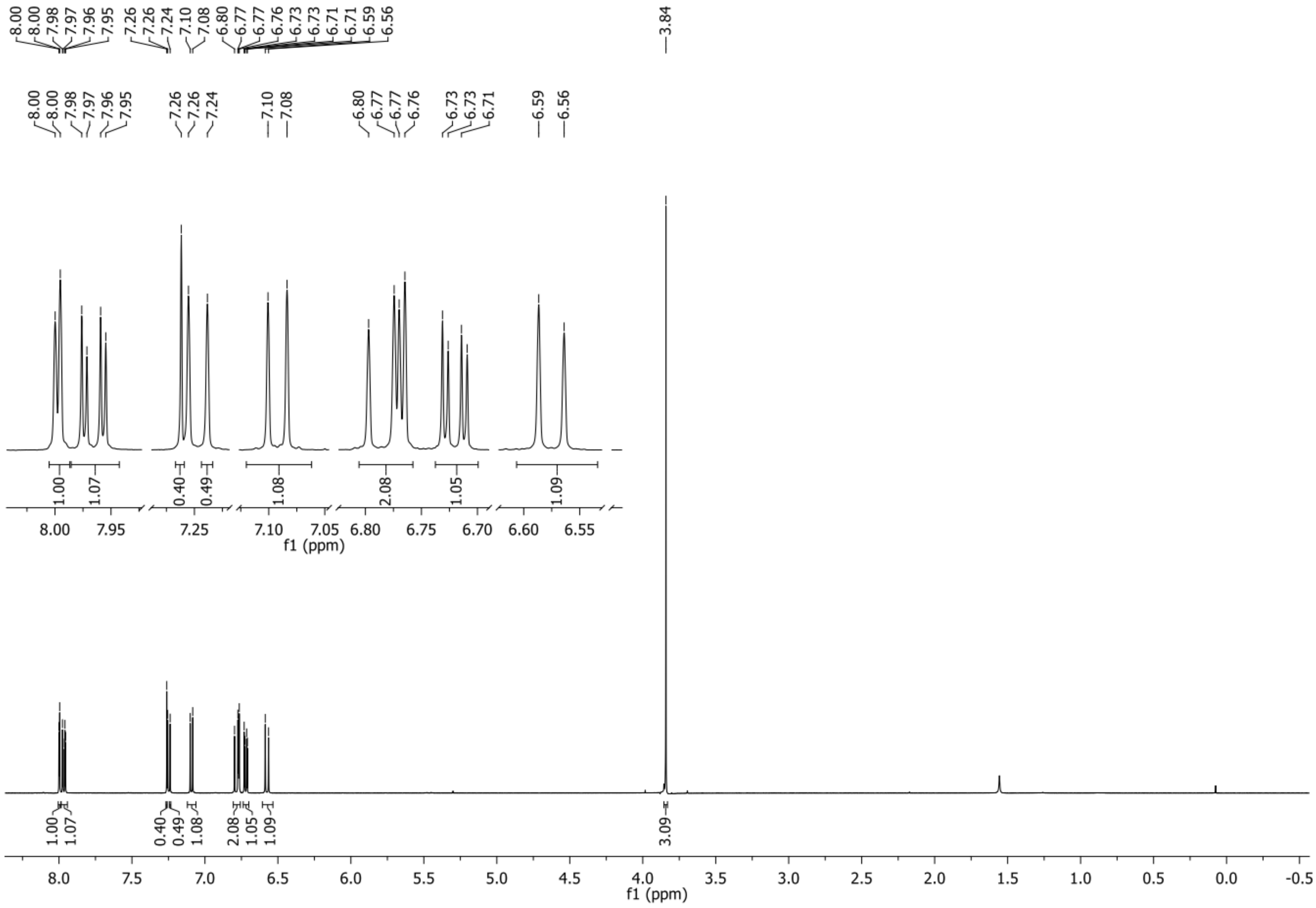

Figure S21. The ^{1}H NMR of 3-methoxy-7-nitrodibenzo[*b*,*f*]oxepine (**2c**).

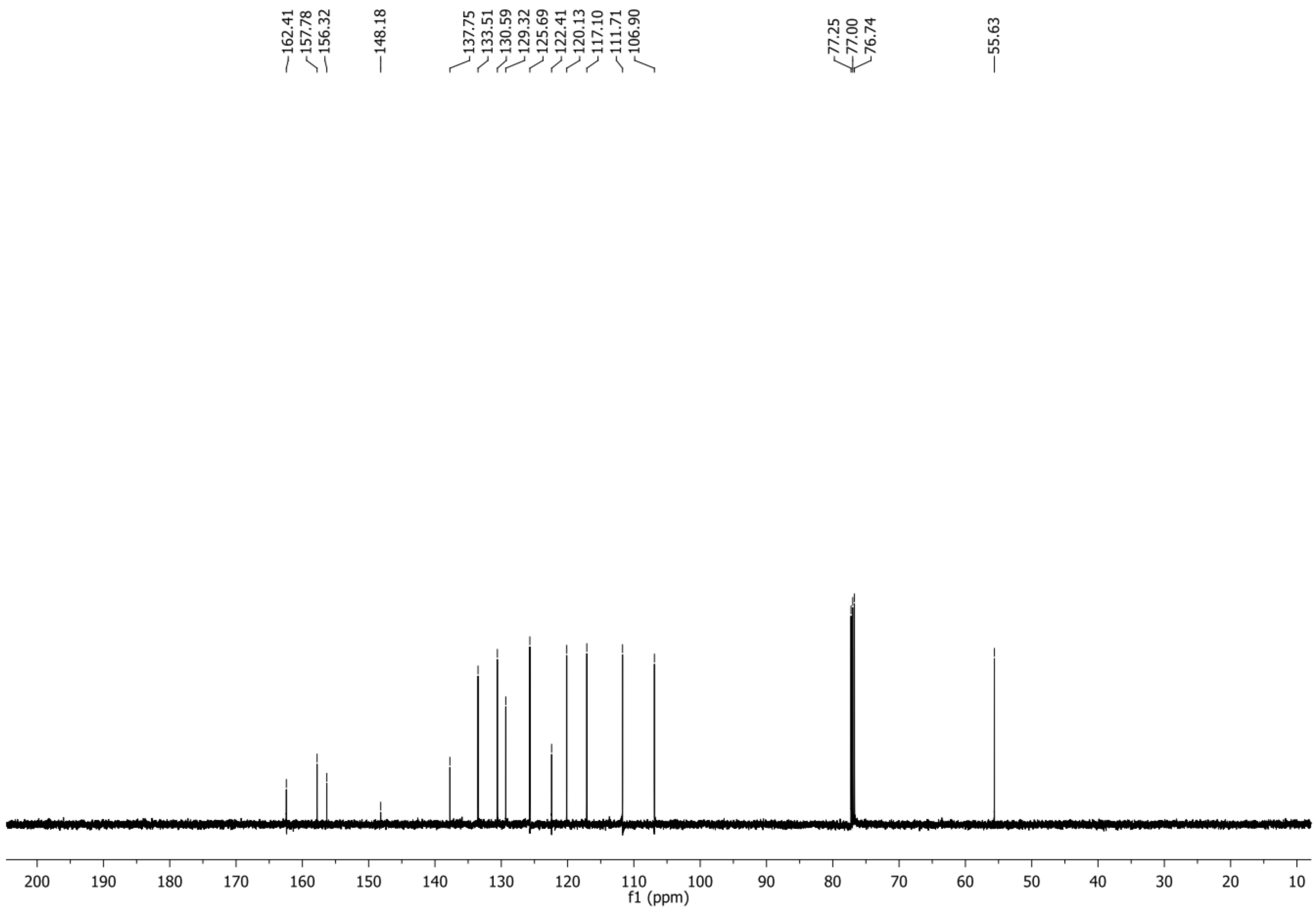

Figure S22. The ^{13}C NMR of 3-methoxy-7-nitrodibenzo[*b,f*]oxepine (**2c**).

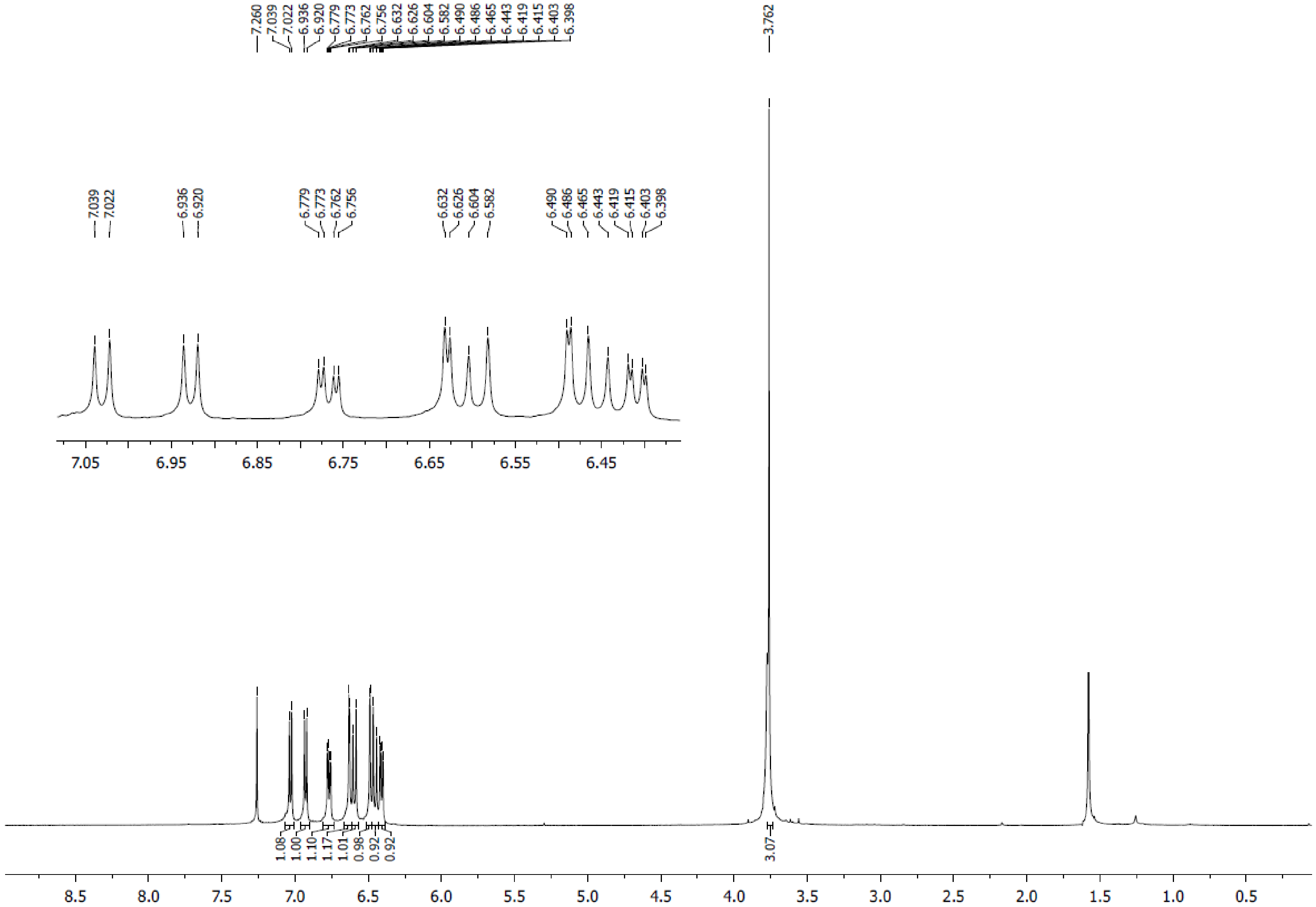

Figure S23. The ^{1}H NMR of 2-methoxy-7-nitrodibenzo[*b*,*f*]oxepine (**2d**).

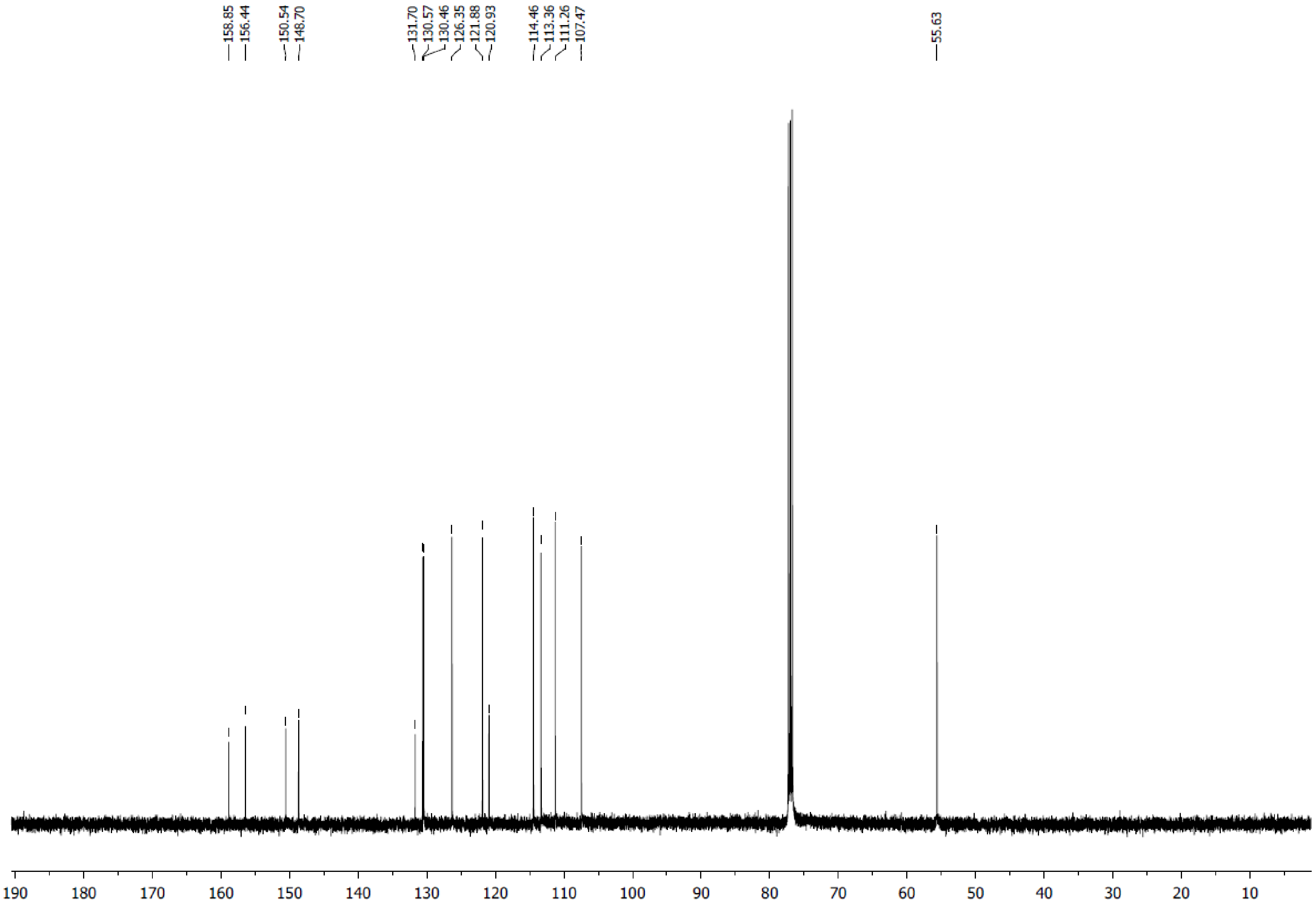

Figure S24. The ^{13}C NMR of 2-methoxy-7-nitrodibenzo[*b*,*f*]oxepine (**2d**).

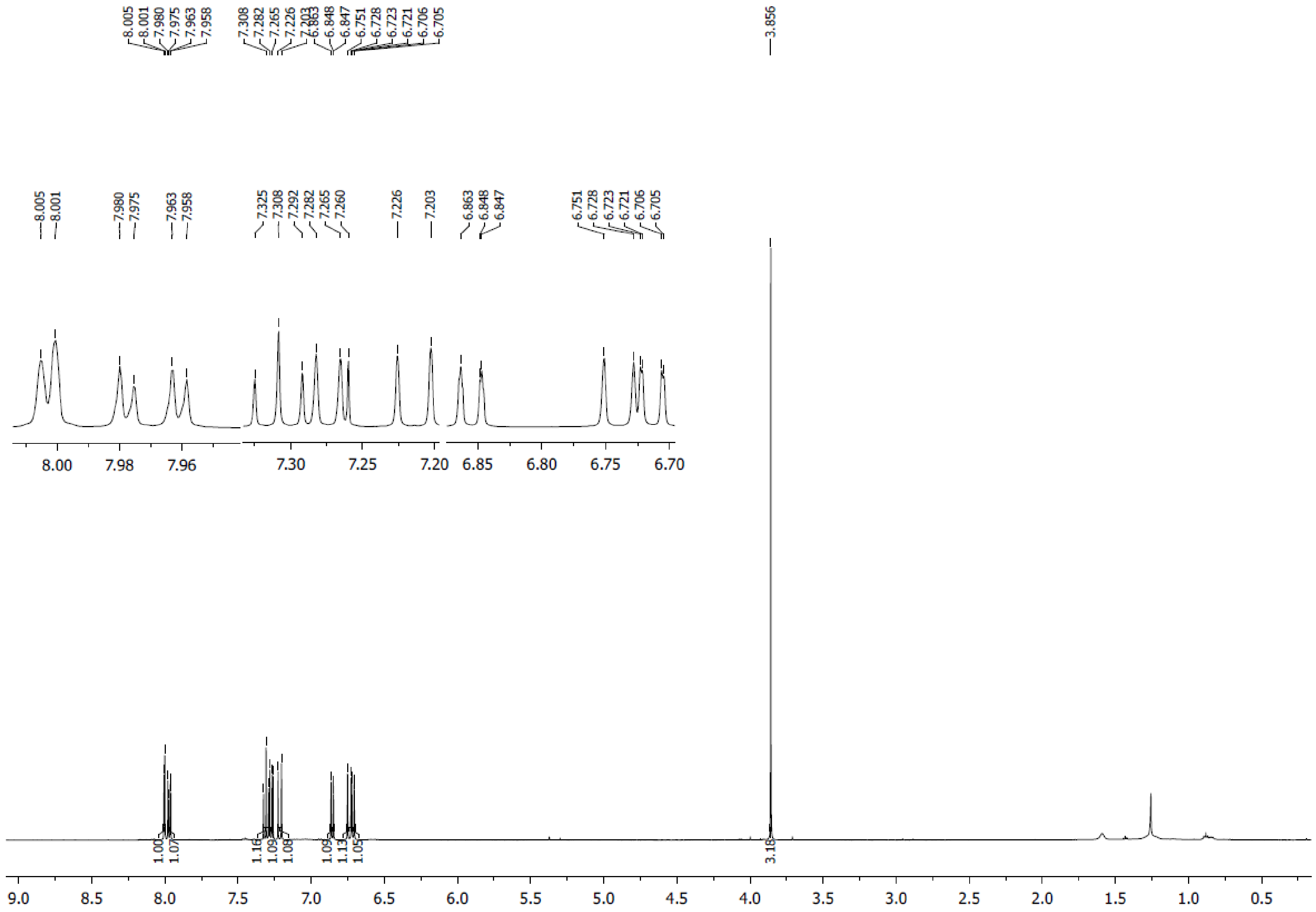

Figure S25. The ^{1}H NMR of 1-methoxy-7-nitrodibenzo[*b,f*]oxepine (**2e**).

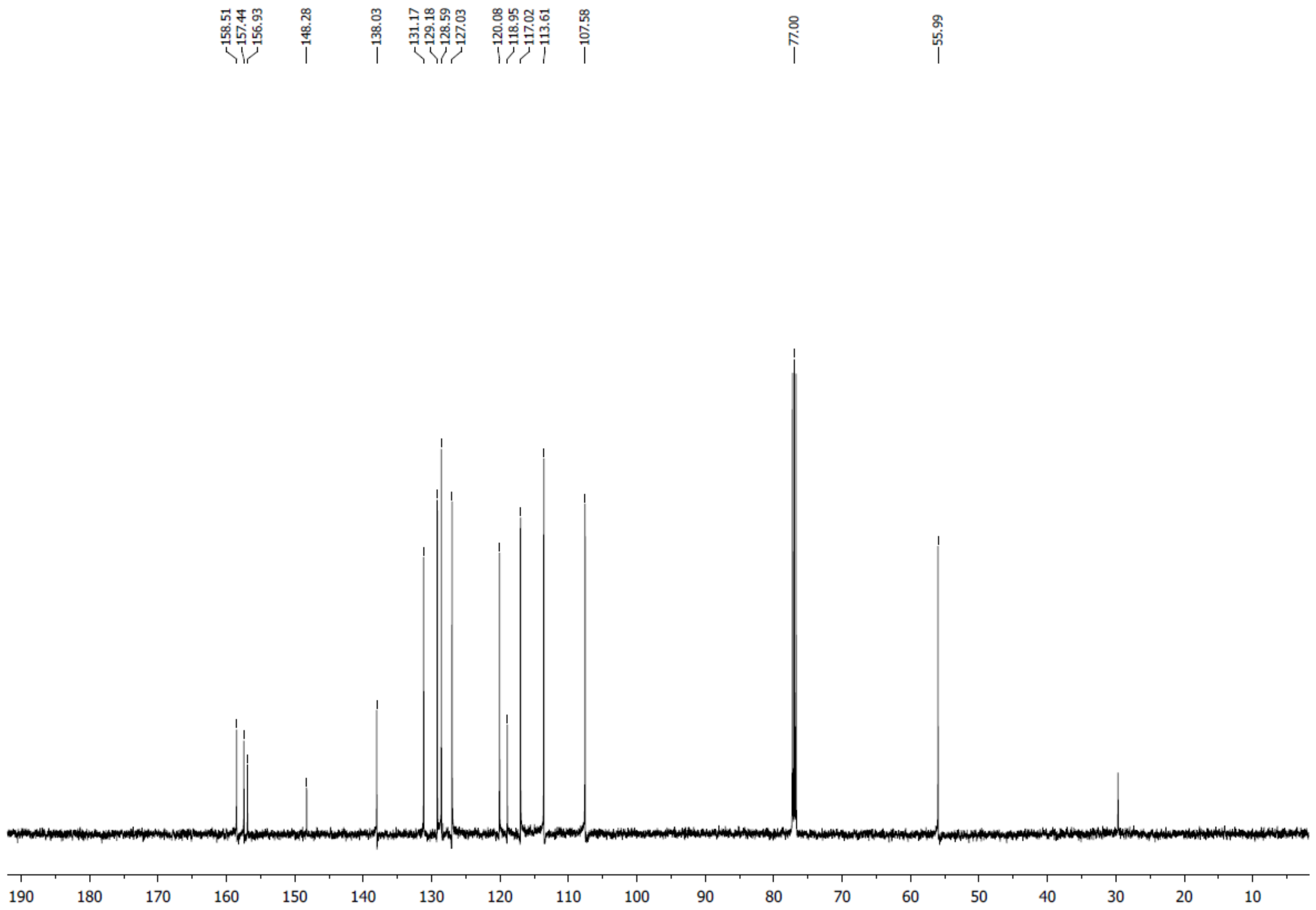

Figure S26. The ^{13}C NMR of 1-methoxy-7-nitrodibenzo[*b*,*f*]oxepine (**2e**).

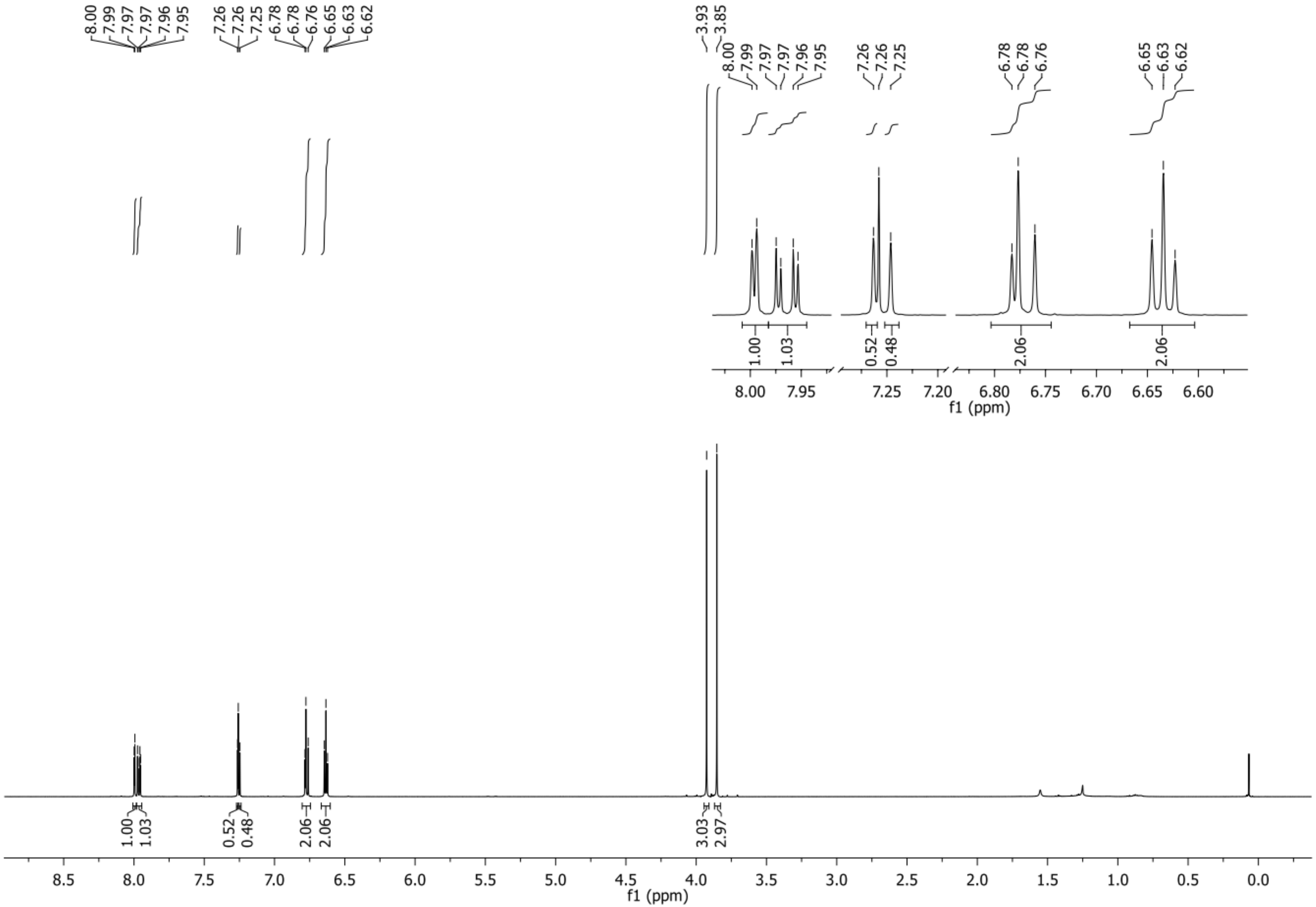

Figure S27. The ^{1}H NMR of 2,3-dimethoxy-7-nitrodibenzo[*b*,*f*]oxepine (**2f**).

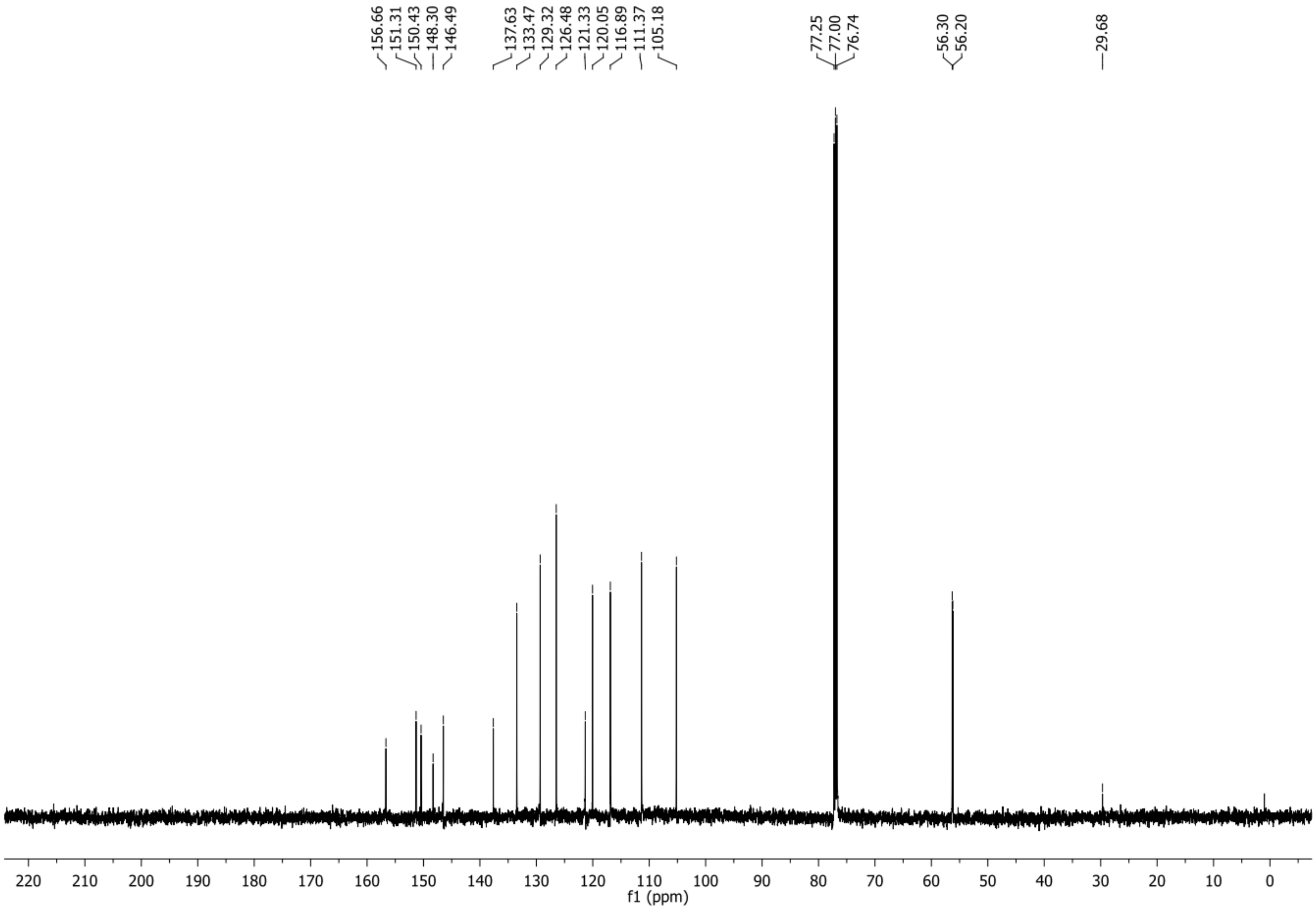

Figure S28. The ^{13}C NMR of 2,3-dimethoxy-7-nitrodibenzo[*b*,*f*]oxepine (**2f**).

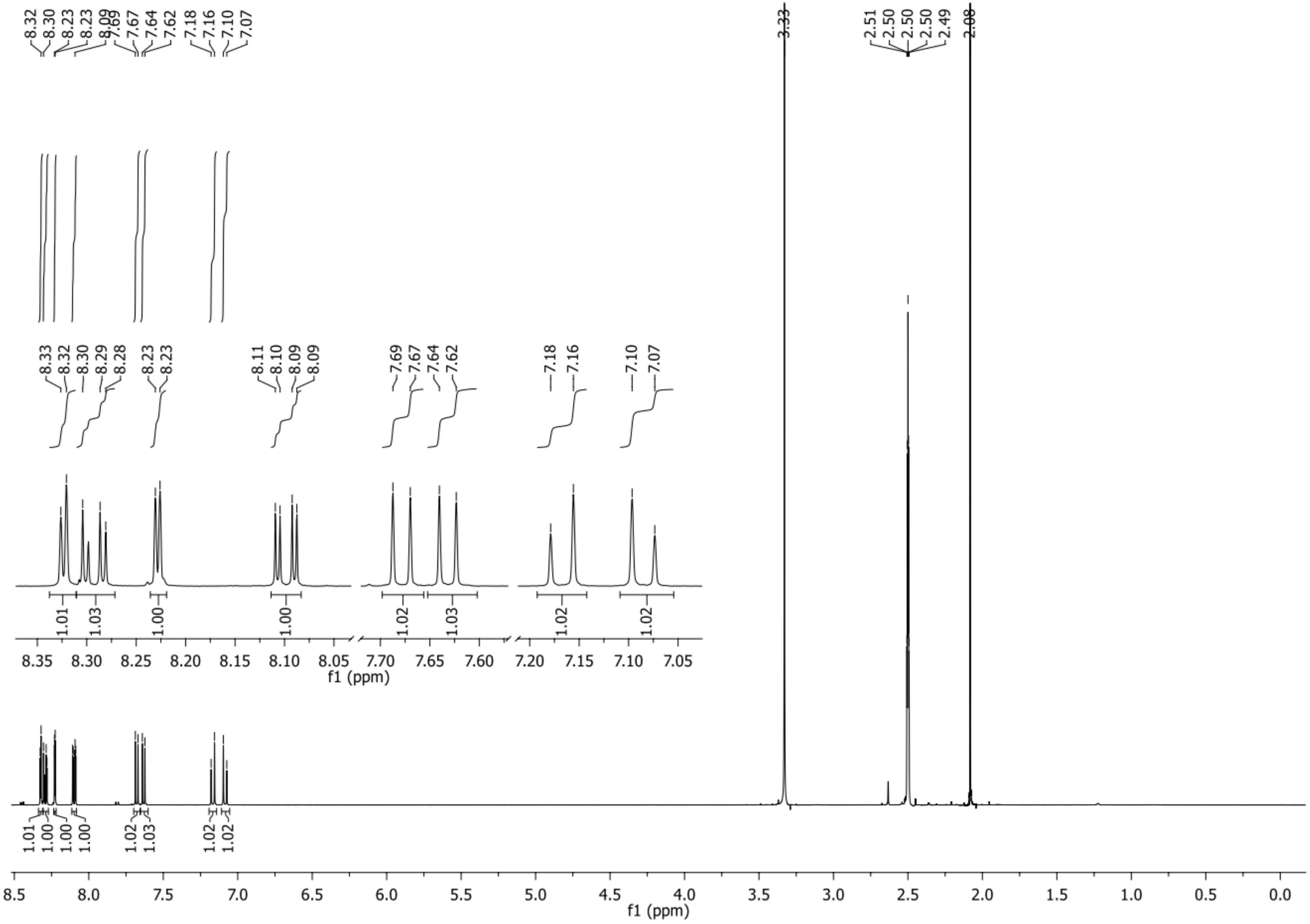

Figure S29. The ^{1}H NMR of 2,7-dinitrodibenzo[*b,f*]oxepine (**2g**).

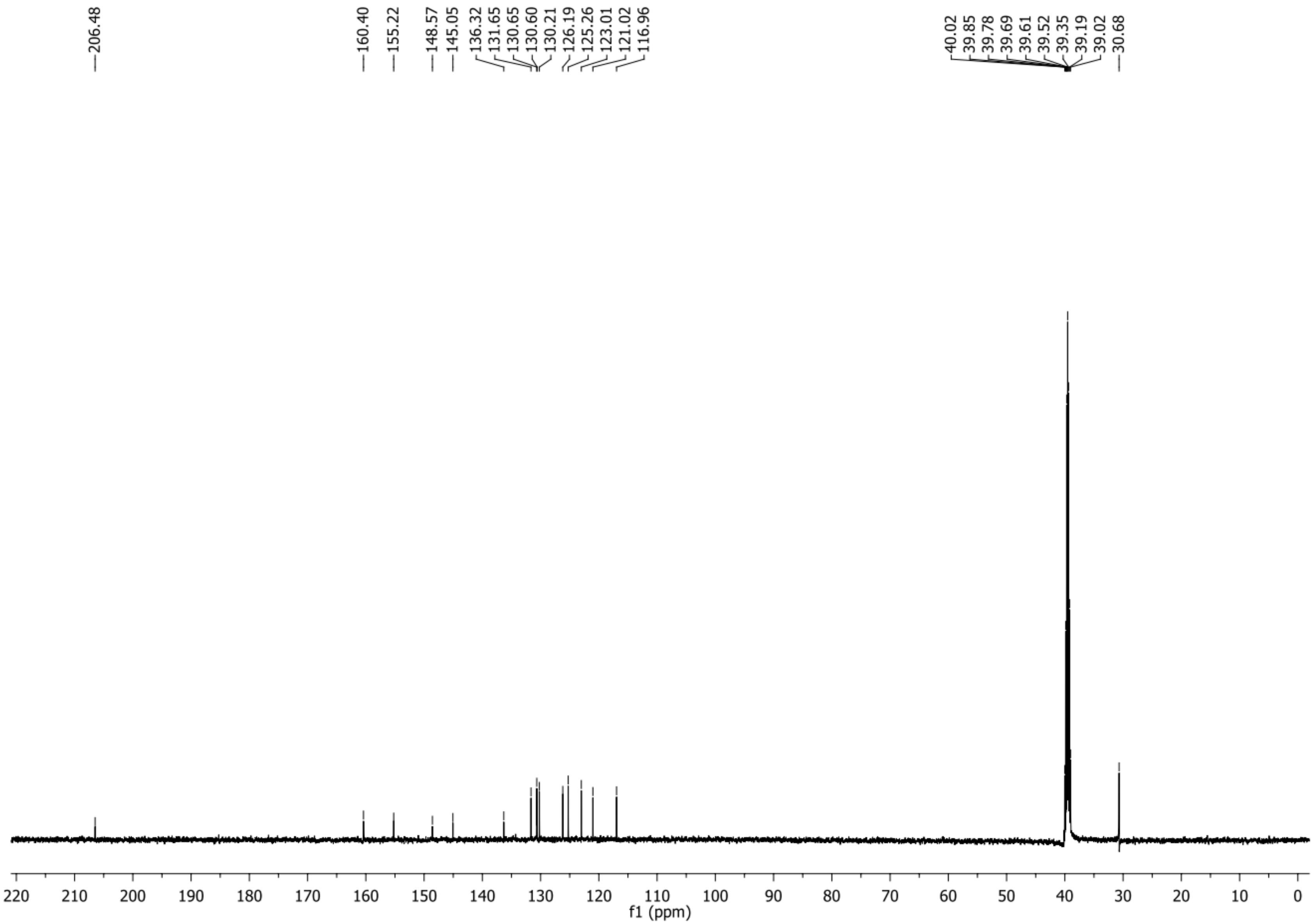

Figure S30. The ^{13}C NMR of 2,7-dinitrodibenzo[*b,f*]oxepine (**2g**).

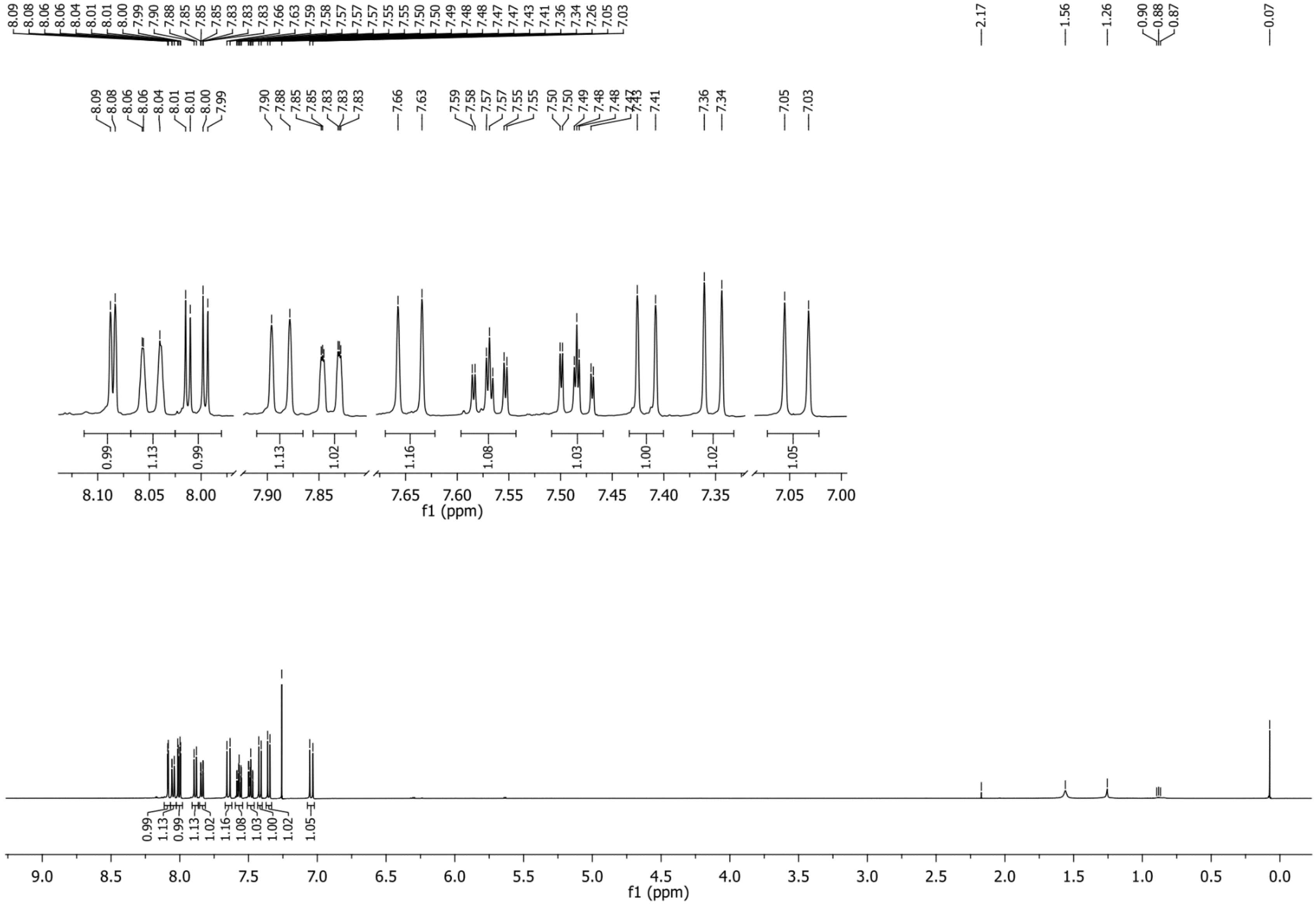

Figure S31. The ^{1}H NMR of 9-nitrobenzo[*b*]naphtho[*1,2-f*]oxepine (**2h**).

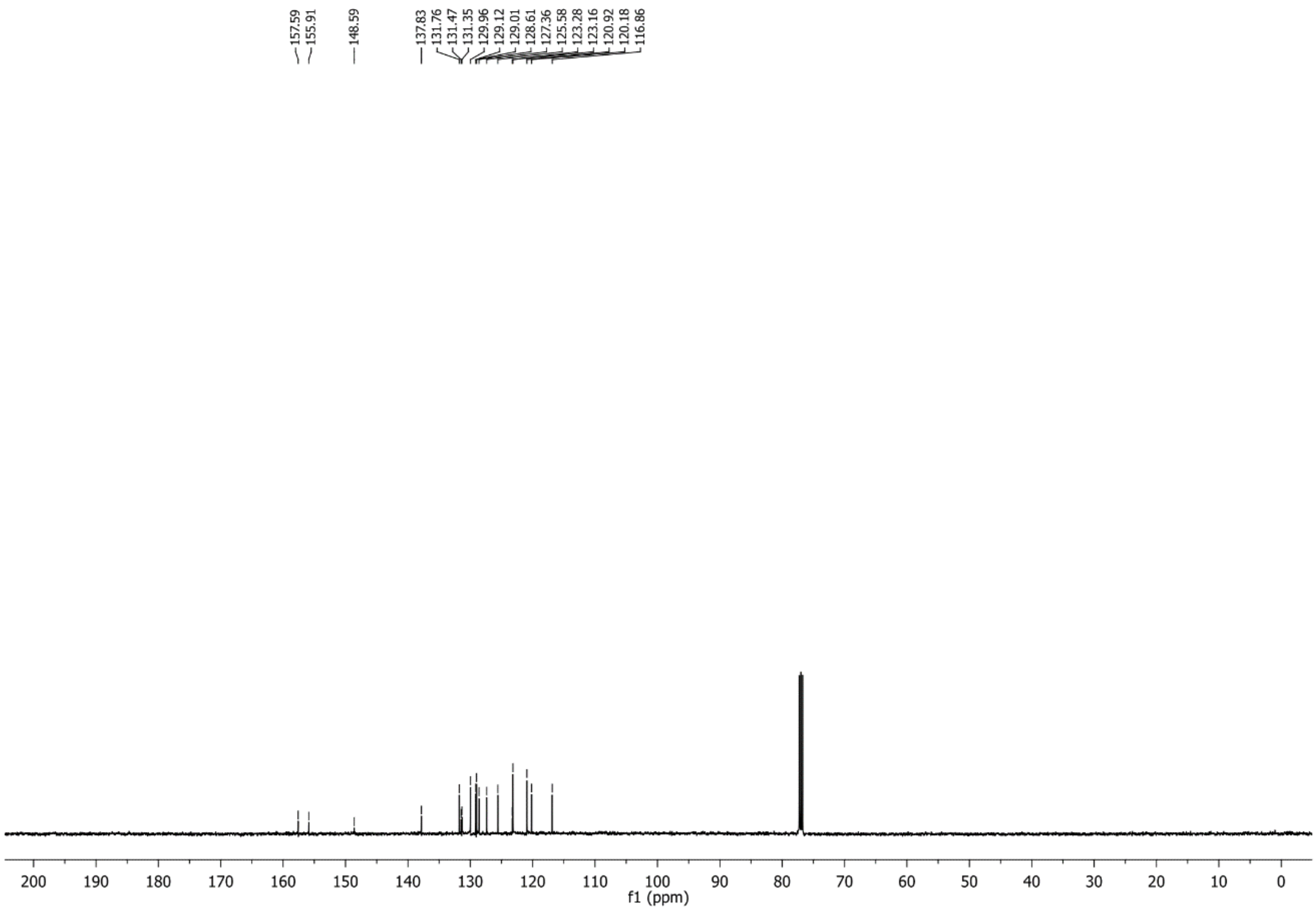

Figure S32. The ^{13}C NMR of 9-nitrobenzo[*b*]naphtho[*1,2-f*]oxepine (**2h**).

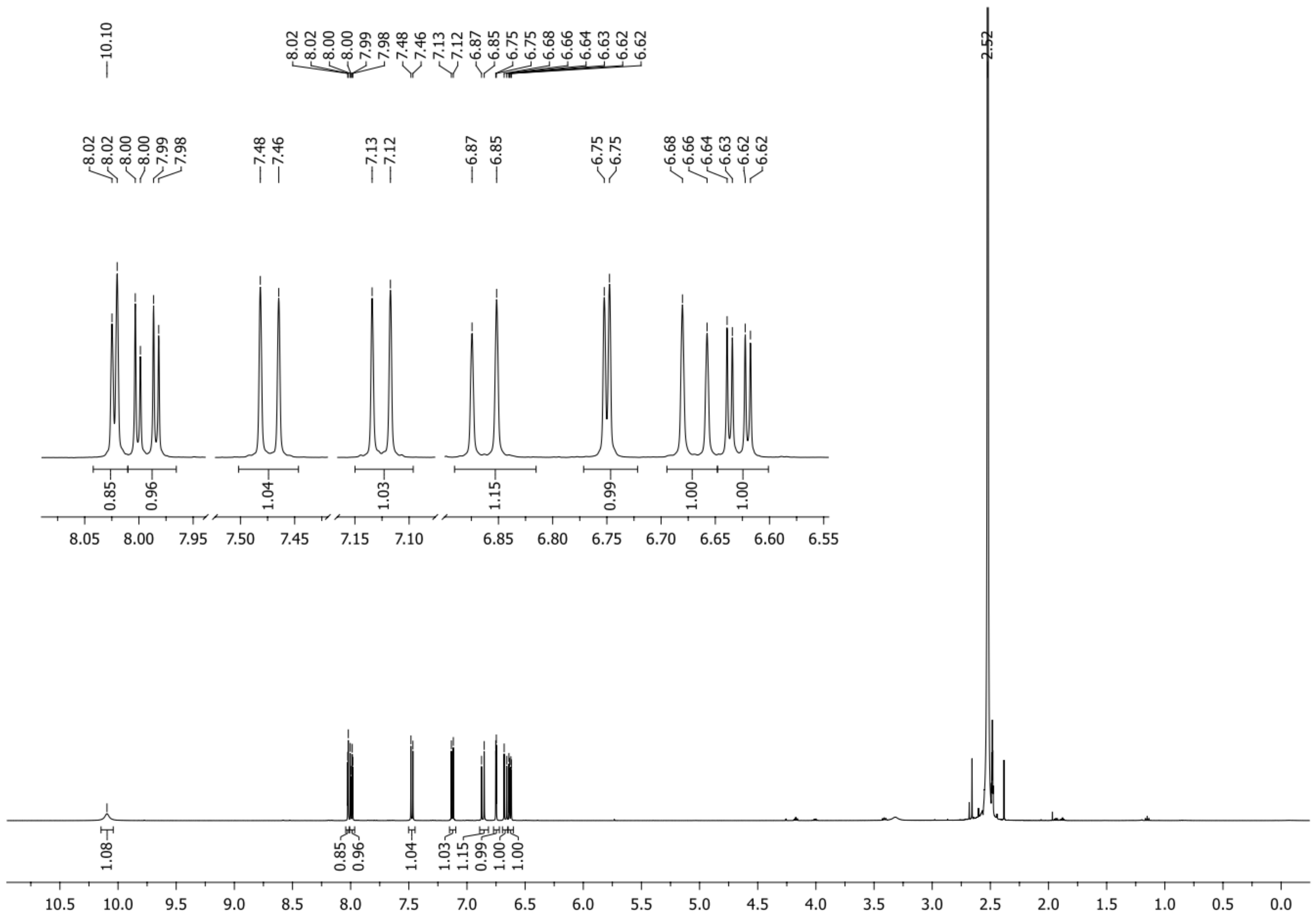

Figure S33. The ^{1}H NMR of 7-nitrodibenzo[*b,f*]oxepin-3-ol (**2i**).

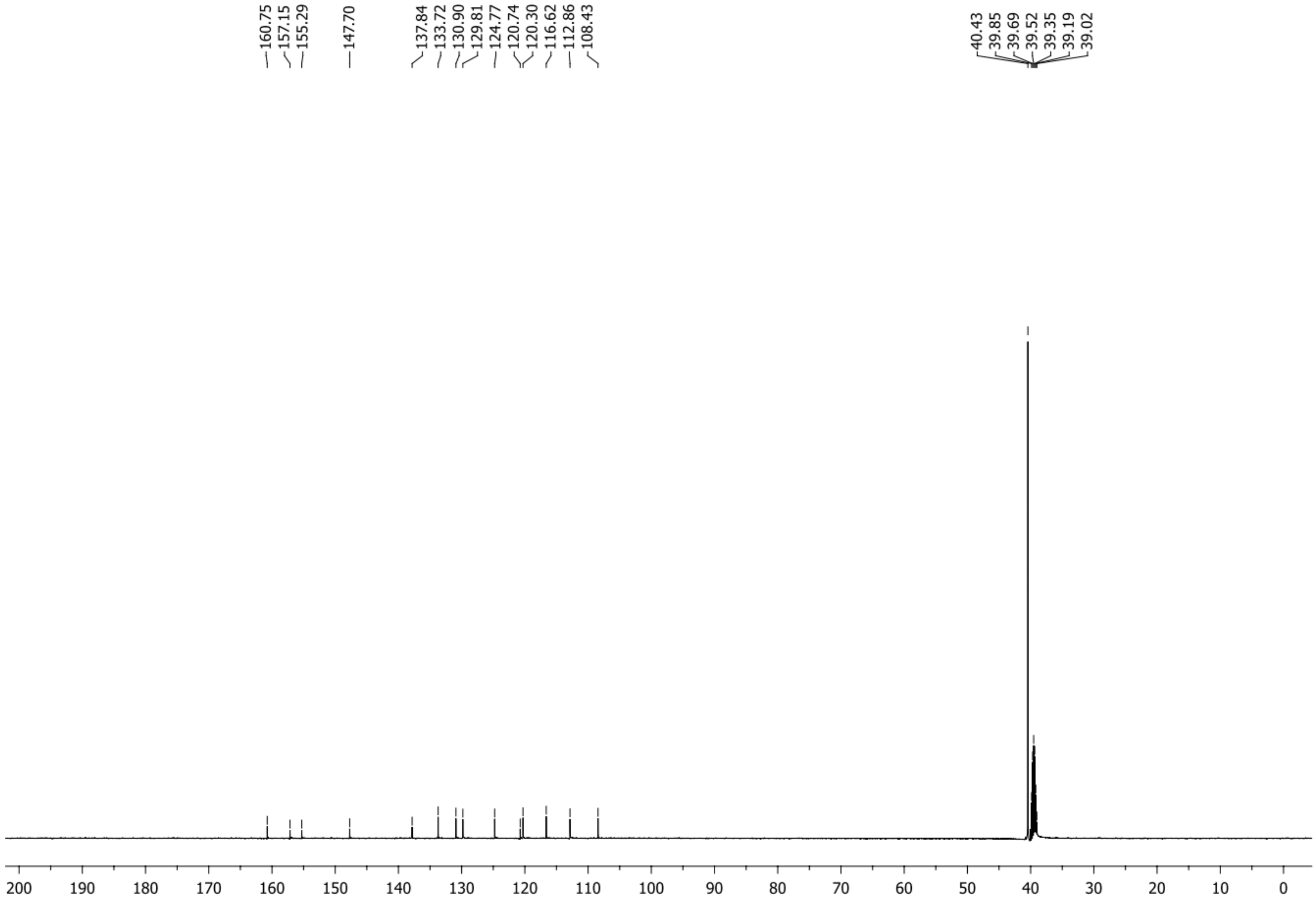

Figure S34. The ^{13}C NMR of 7-nitrodibenzo[*b,f*]oxepin-3-ol (**2i**).

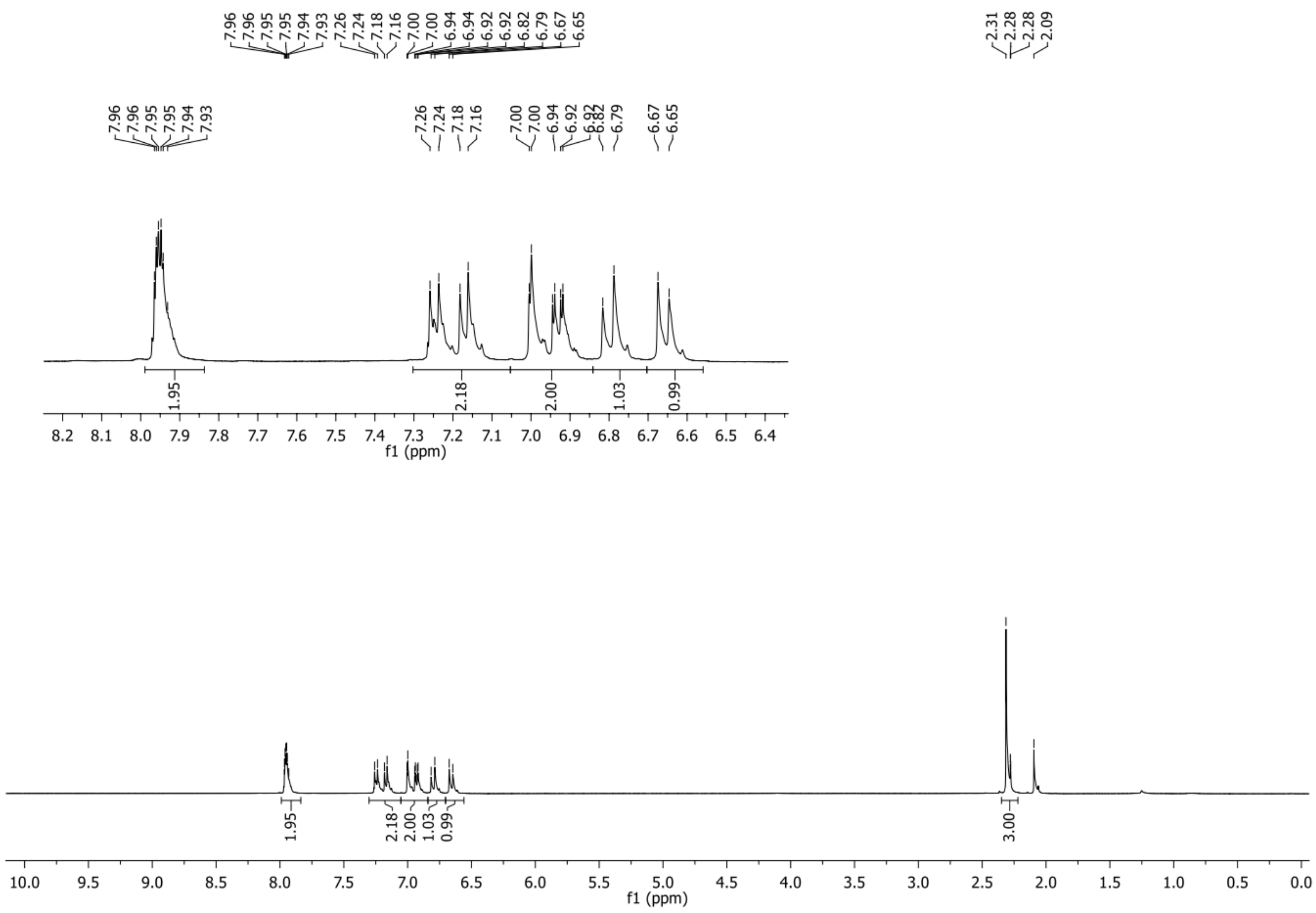

Figure S35. The ^{1}H NMR of 7-nitrodibenzo[*b,f*]oxepin-3-yl acetate (**2j**).

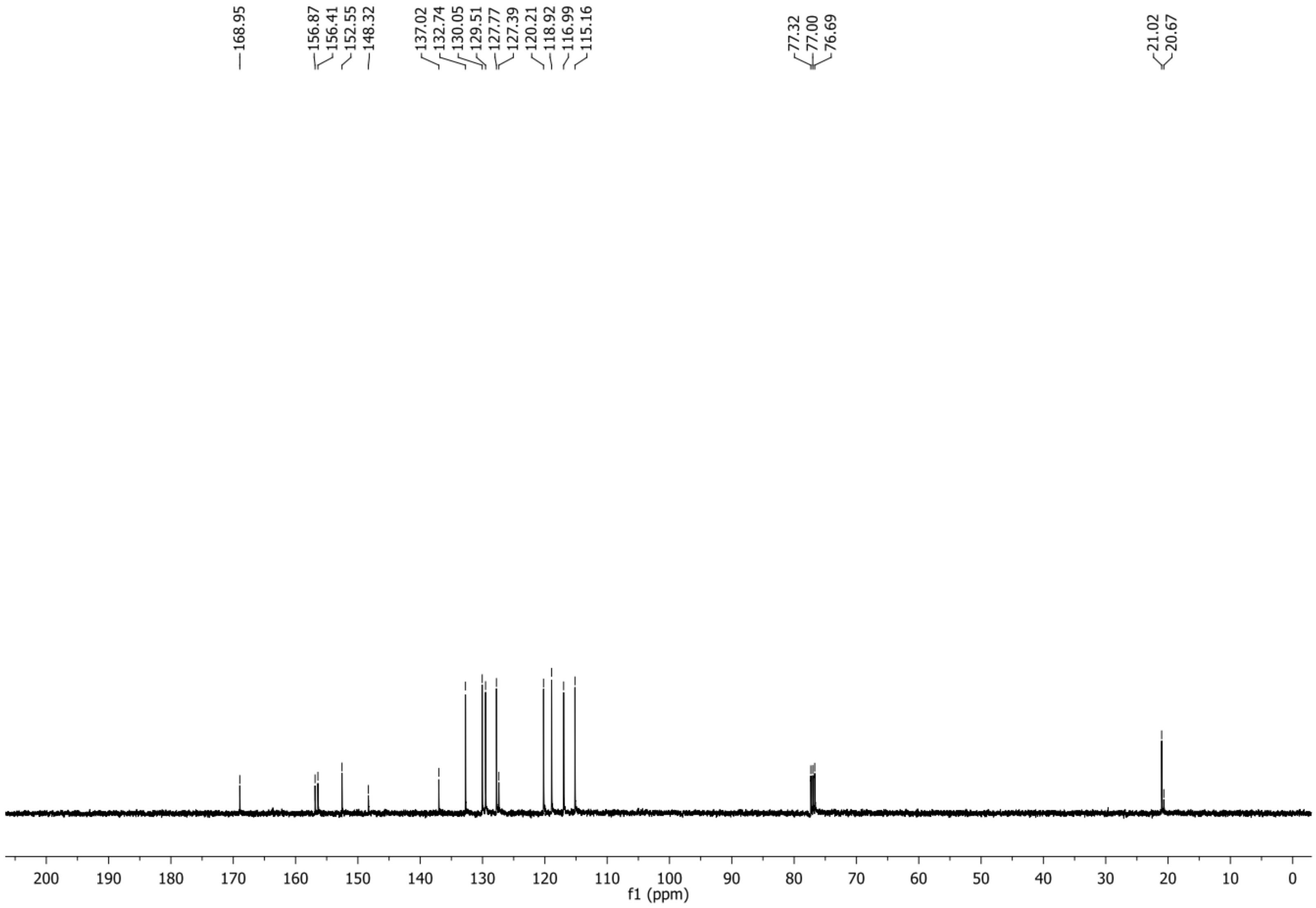

Figure S36. The ^{13}C NMR of 7-nitrodibenzo[*b*,*f*]oxepin-3-yl acetate (**2j**).

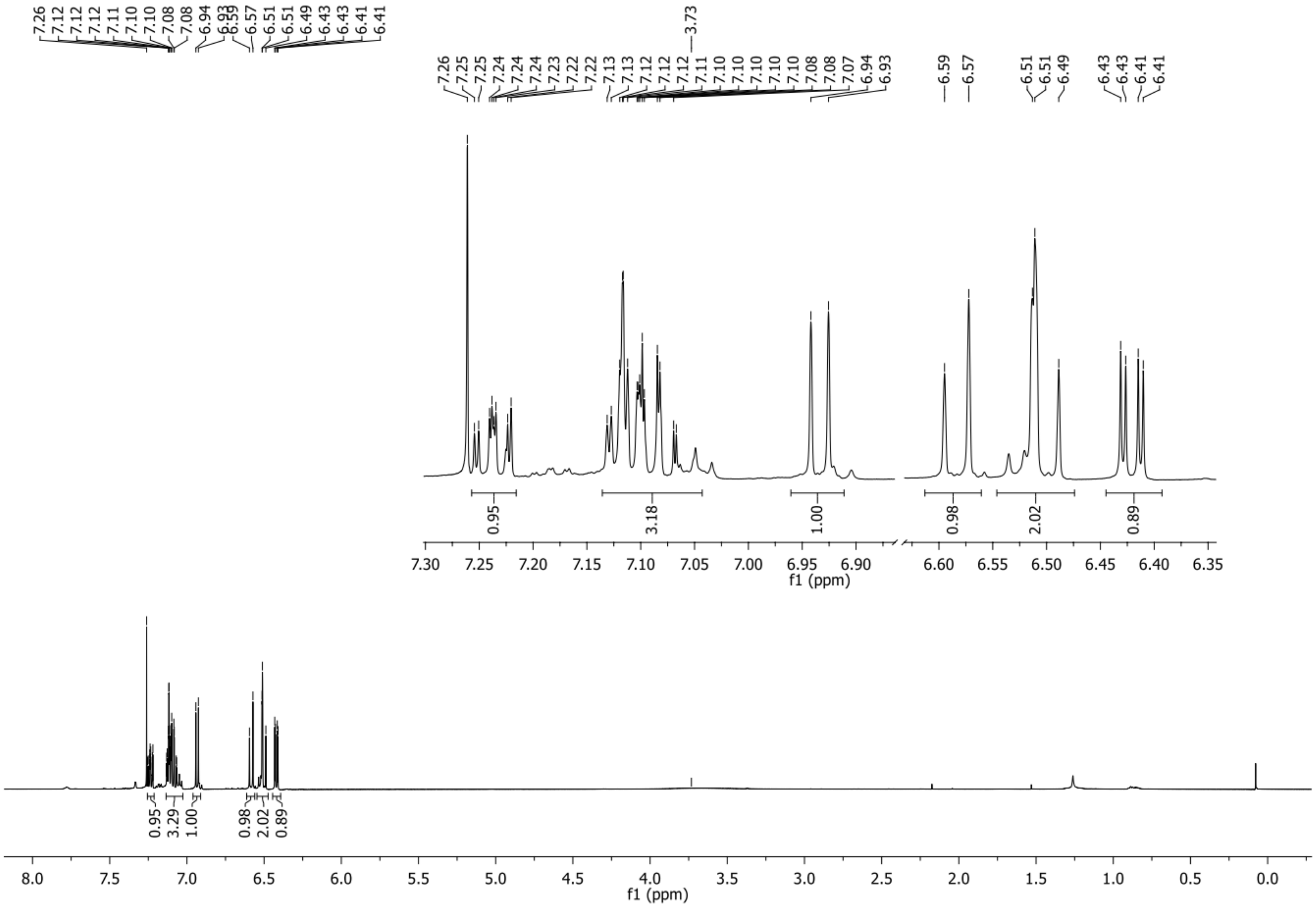

Figure S37. The ^{1}H NMR of dibenzo[*b,f*]oxepin-3-amine (**3a**).

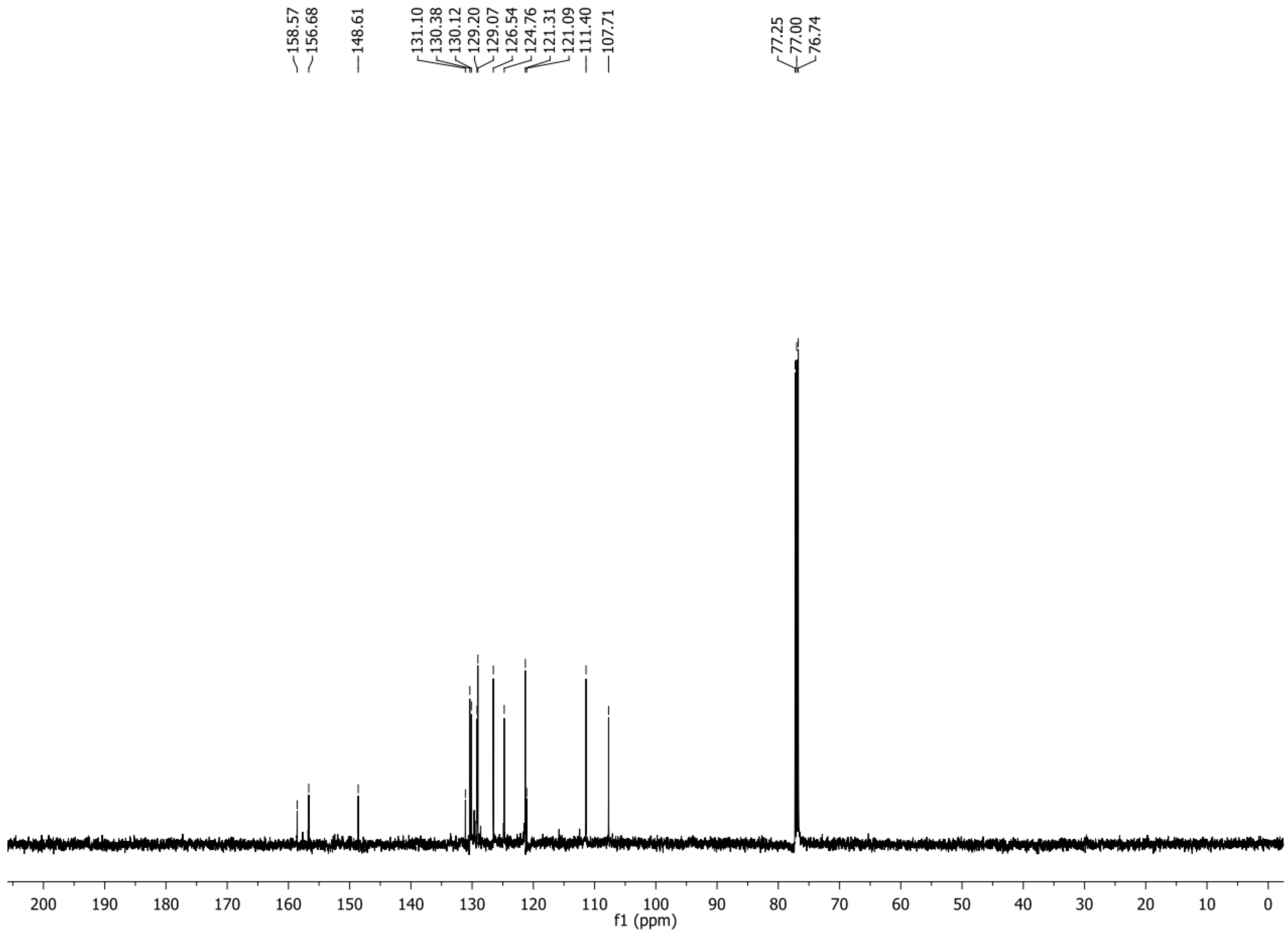

Figure S38. The ^{13}C NMR of dibenzo[*b,f*]oxepin-3-amine (**3a**).

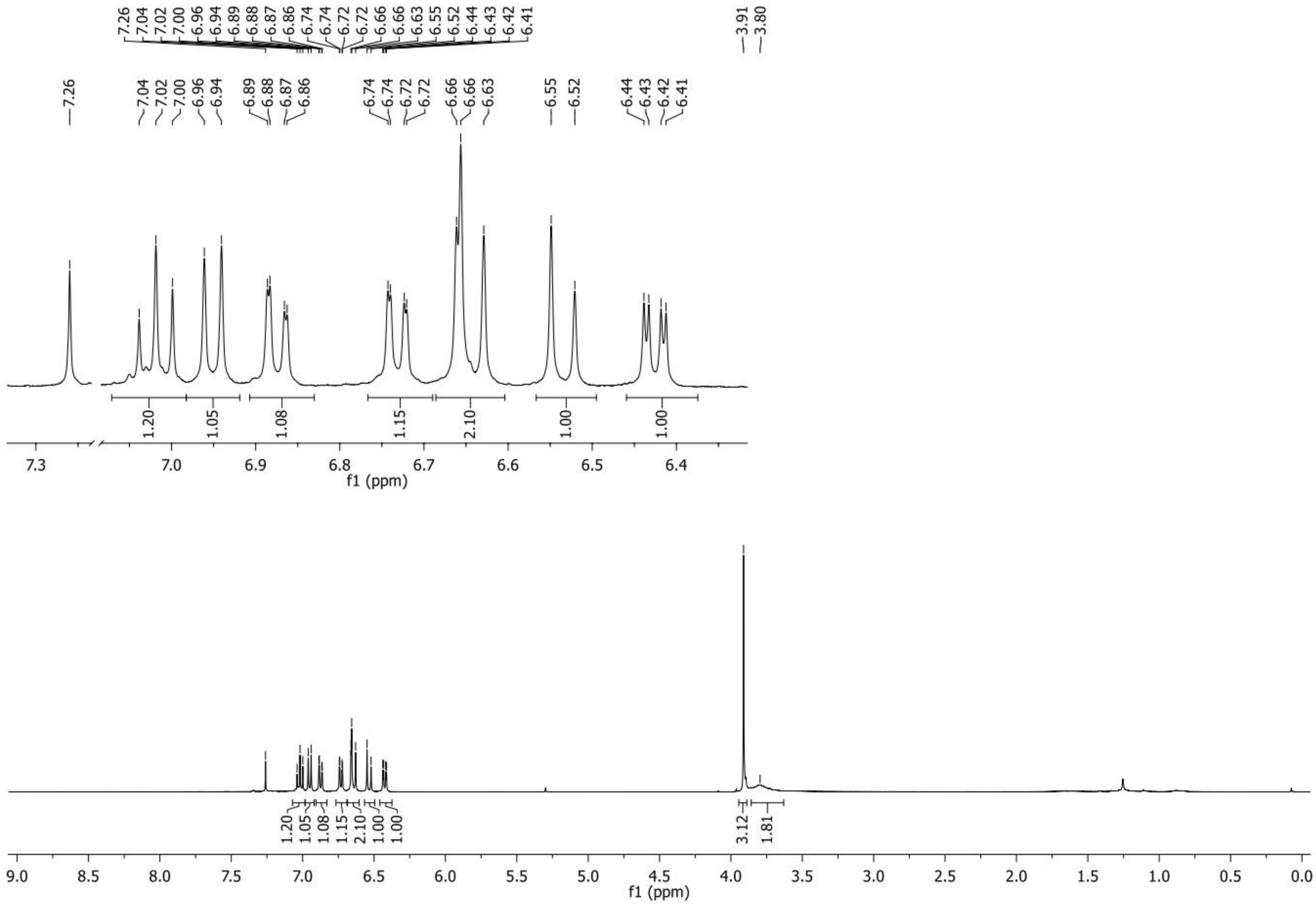

Figure S39. The ^{1}H NMR of 6-methoxydibenzo[*b*,*f*]oxepin-3-amine (**3b**).

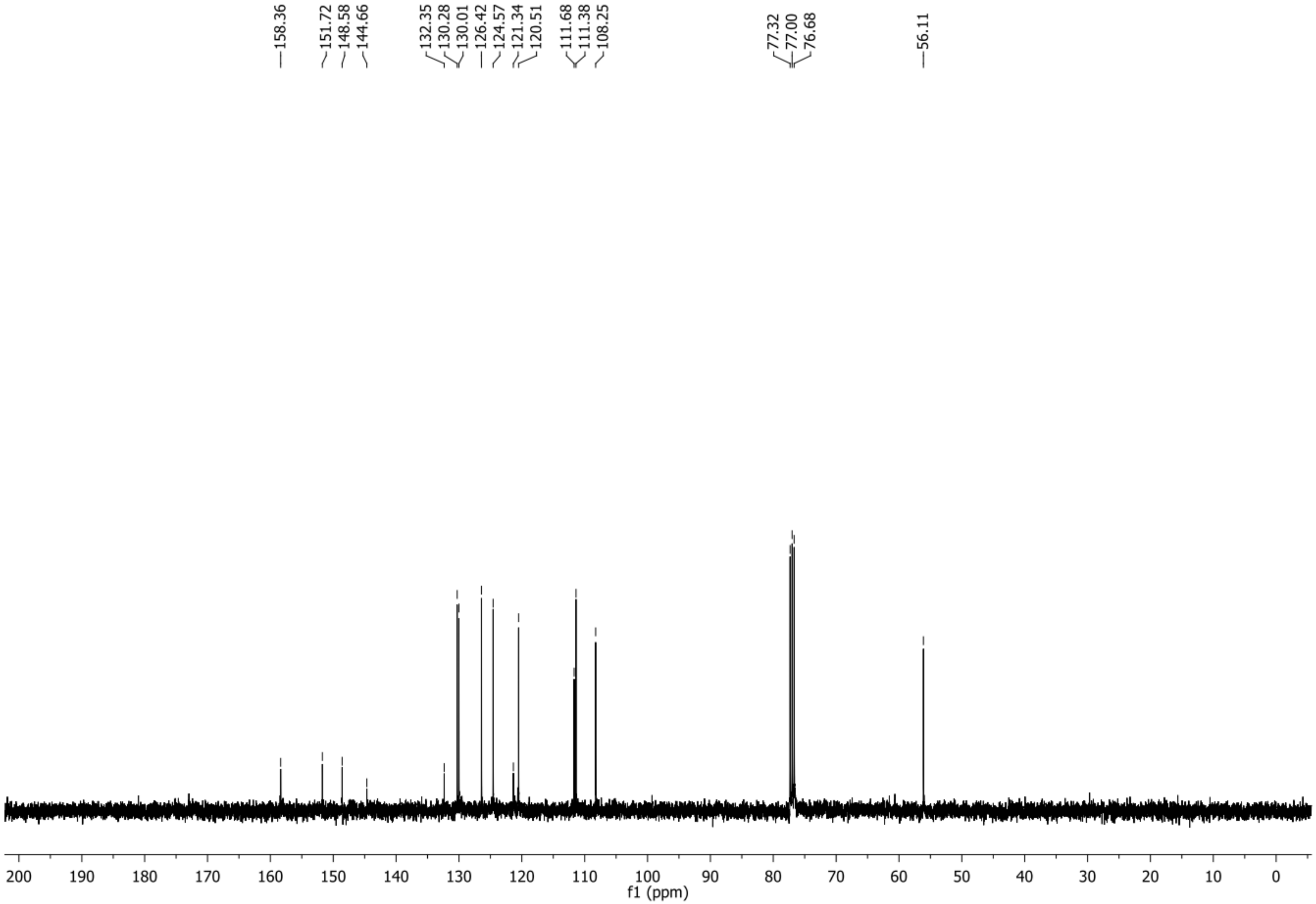

Figure S40. The ^{13}C NMR of 6-methoxydibenzo[*b*,*f*]oxepin-3-amine (**3b**).

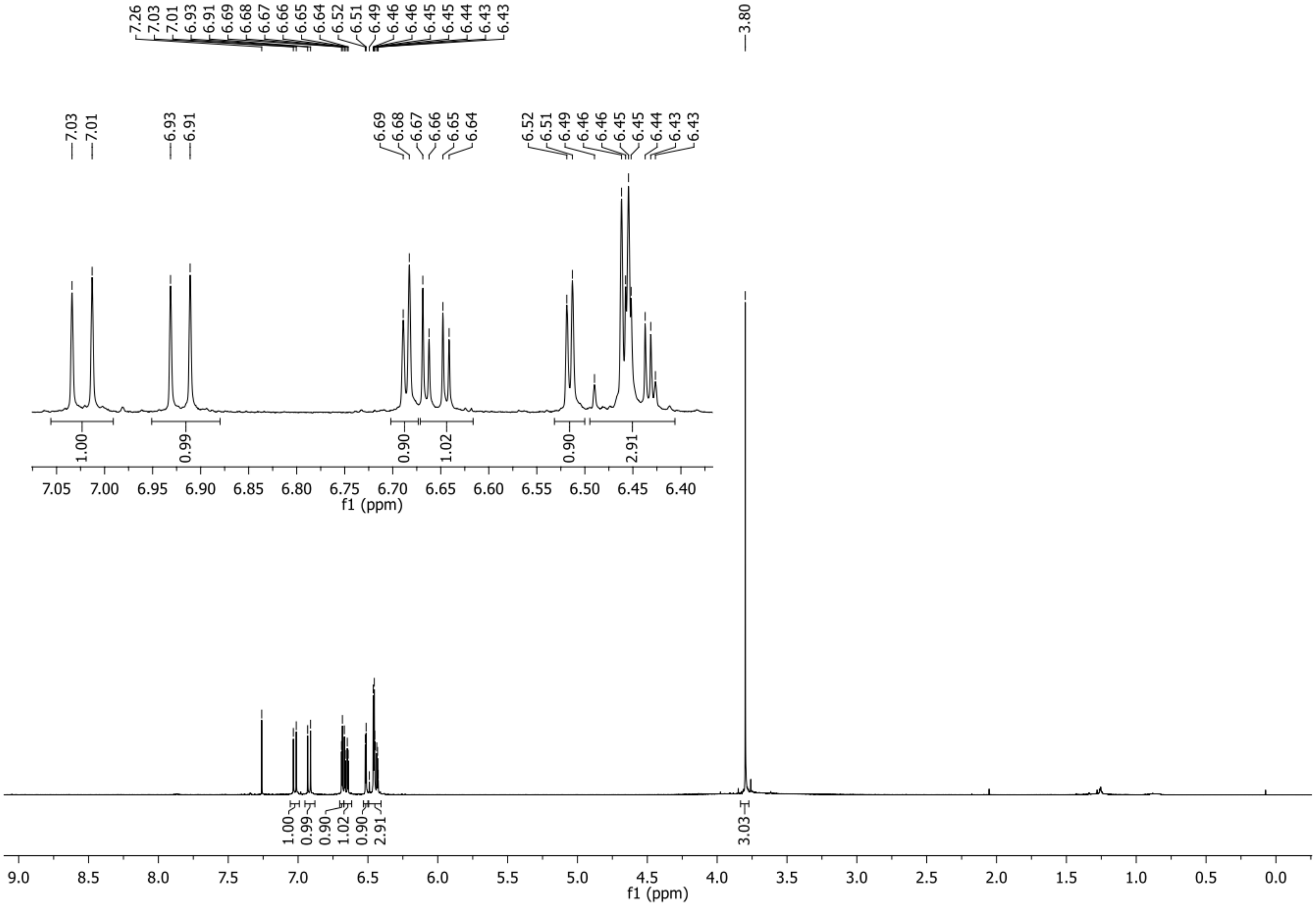

Figure S41. The ^{1}H NMR of 7-methoxydibenzo[*b,f*]oxepin-3-amine (**3c**).

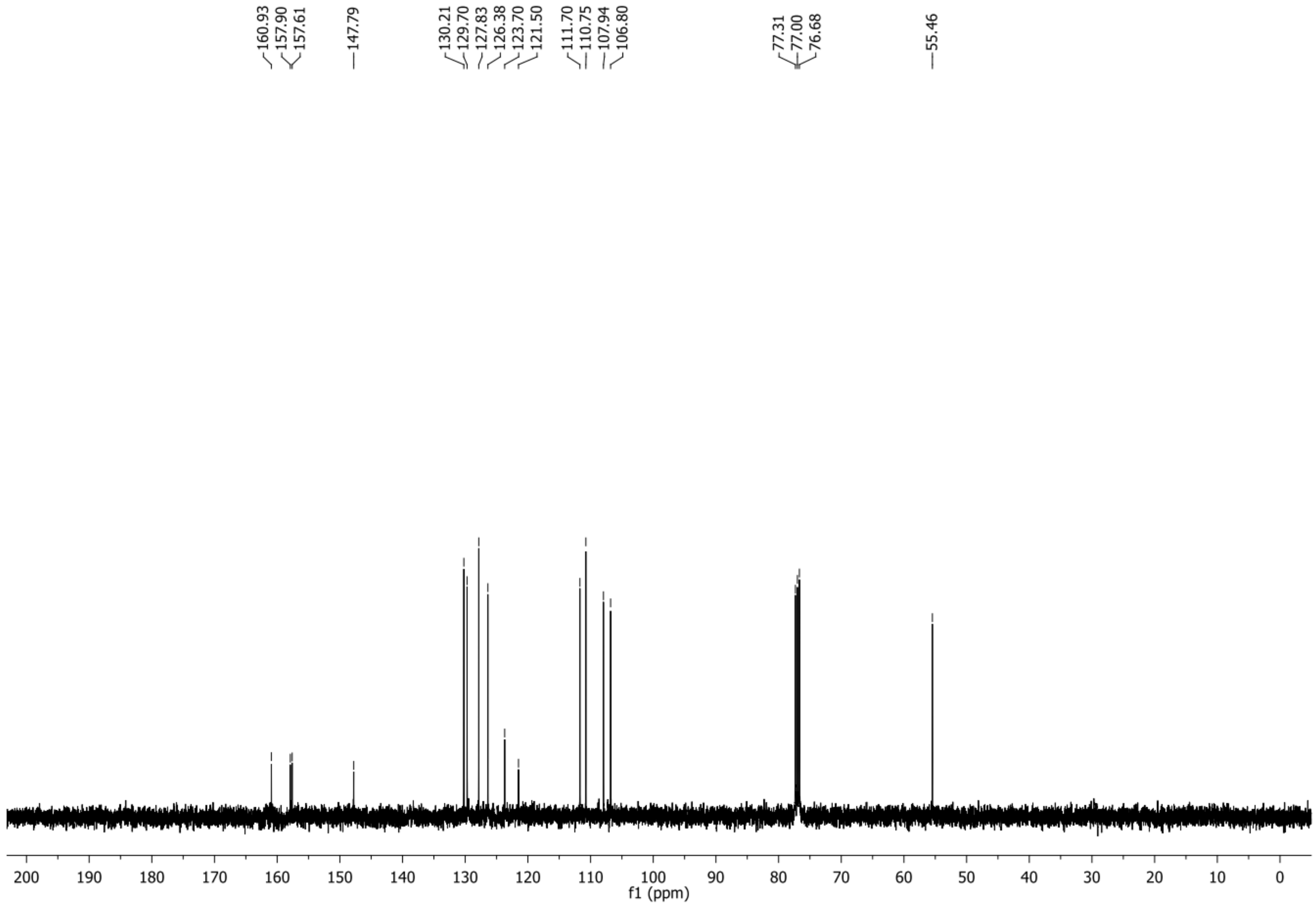

Figure S42. The ^{13}C NMR of 7-methoxydibenzo[*b*,*f*]oxepin-3-amine (**3c**).

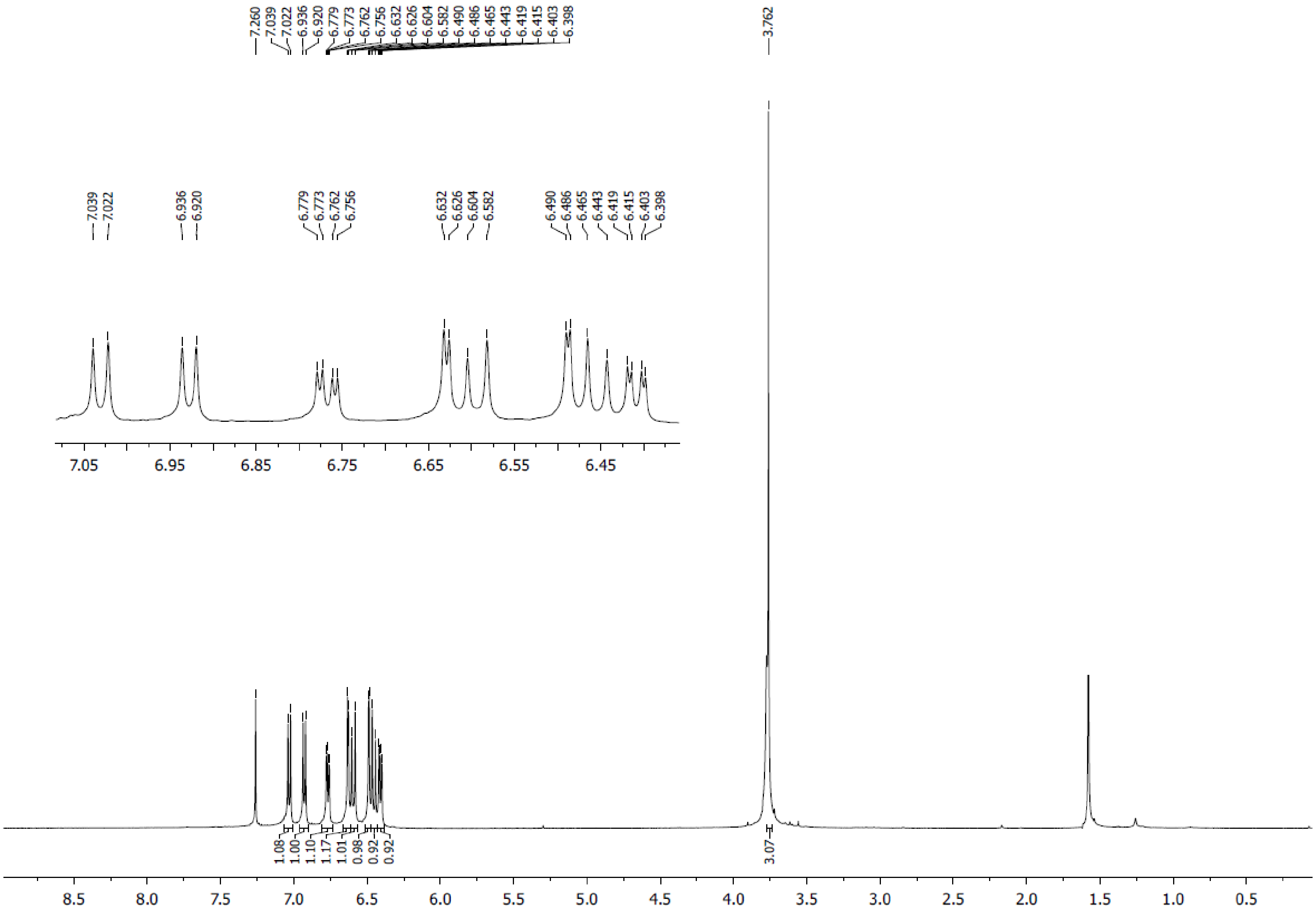

Figure S43. The ^{1}H NMR of 8-methoxydibenzo[*b*,*f*]oxepin-3-amine (**3d**).

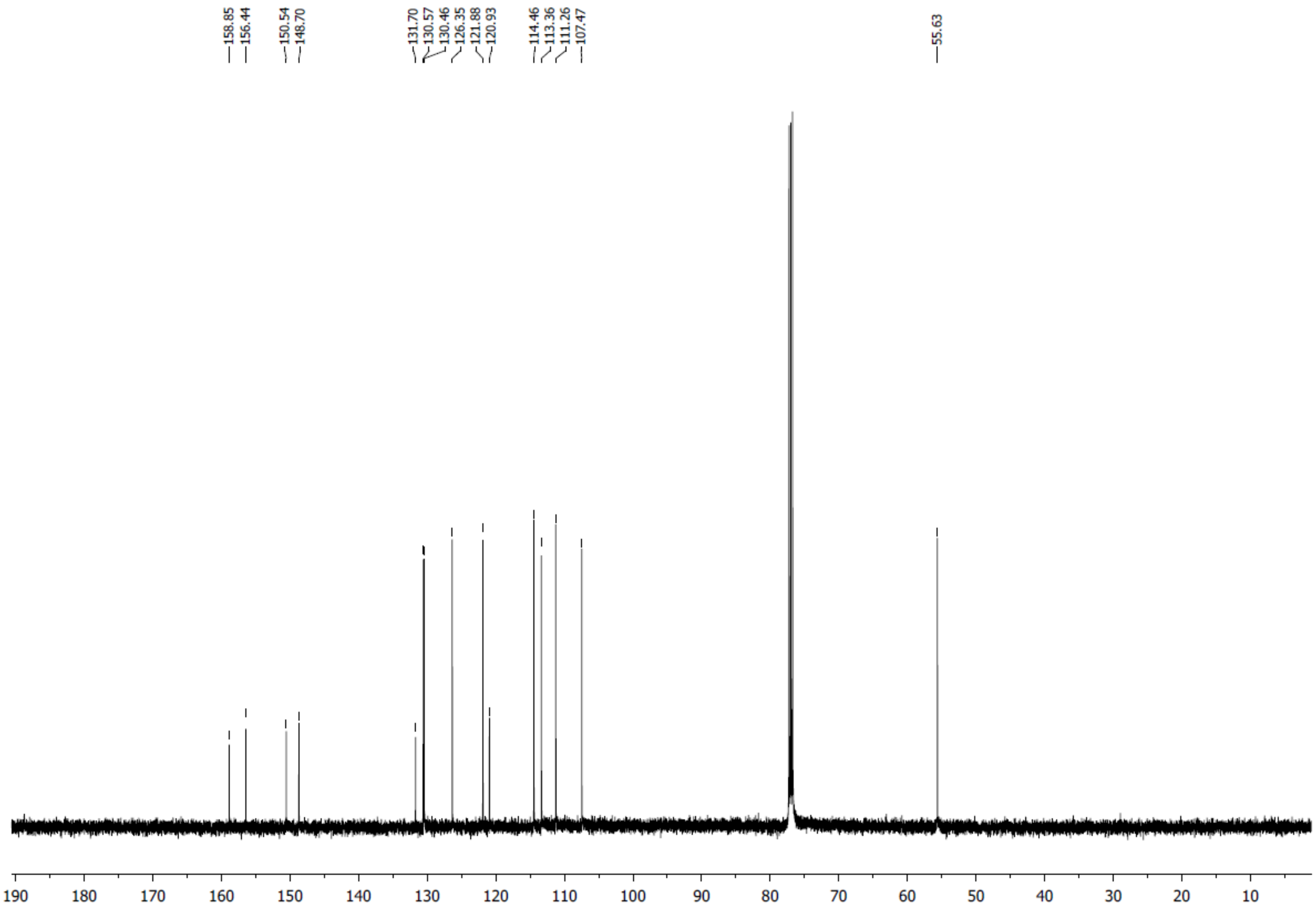

Figure S44. The ^{13}C NMR of 8-methoxydibenzo[*b*,*f*]oxepin-3-amine (**3d**).

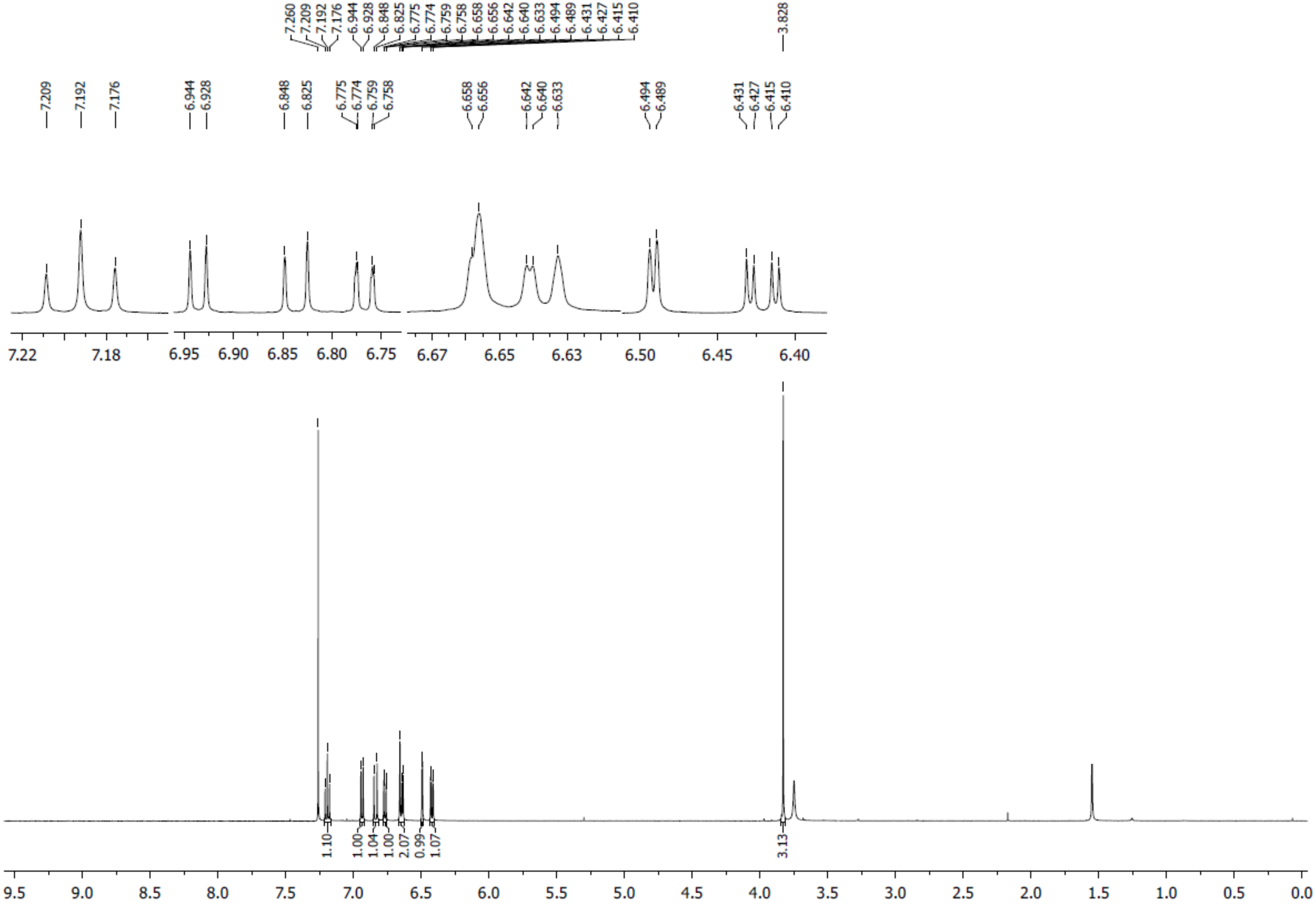

Figure S45. The ^{1}H NMR of 9-methoxydibenzo[*b*,*f*]oxepin-3-amine (**3e**).

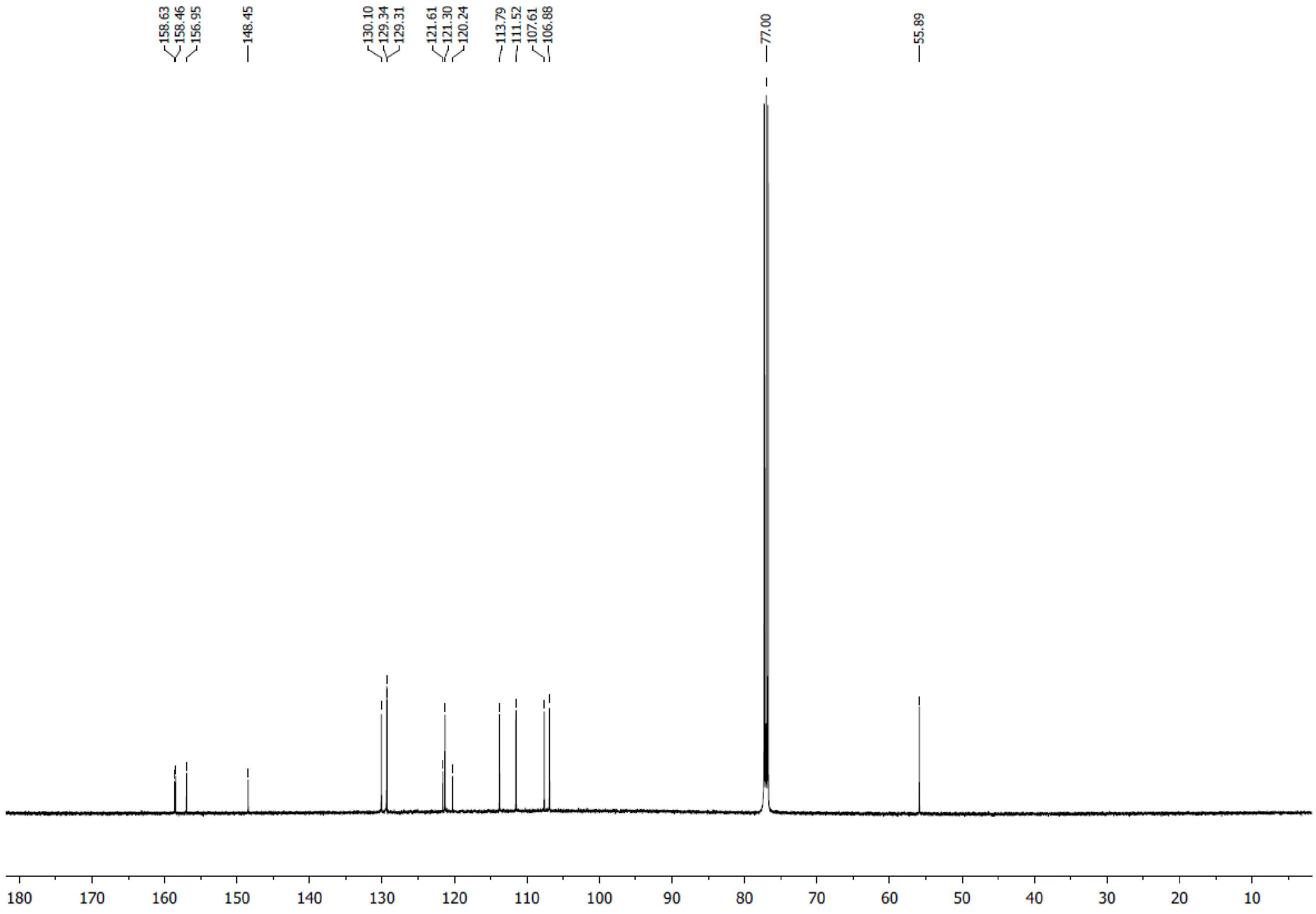

Figure S46. The ^{13}C NMR of 9-methoxydibenzo[*b*,*f*]oxepin-3-amine (**3e**).

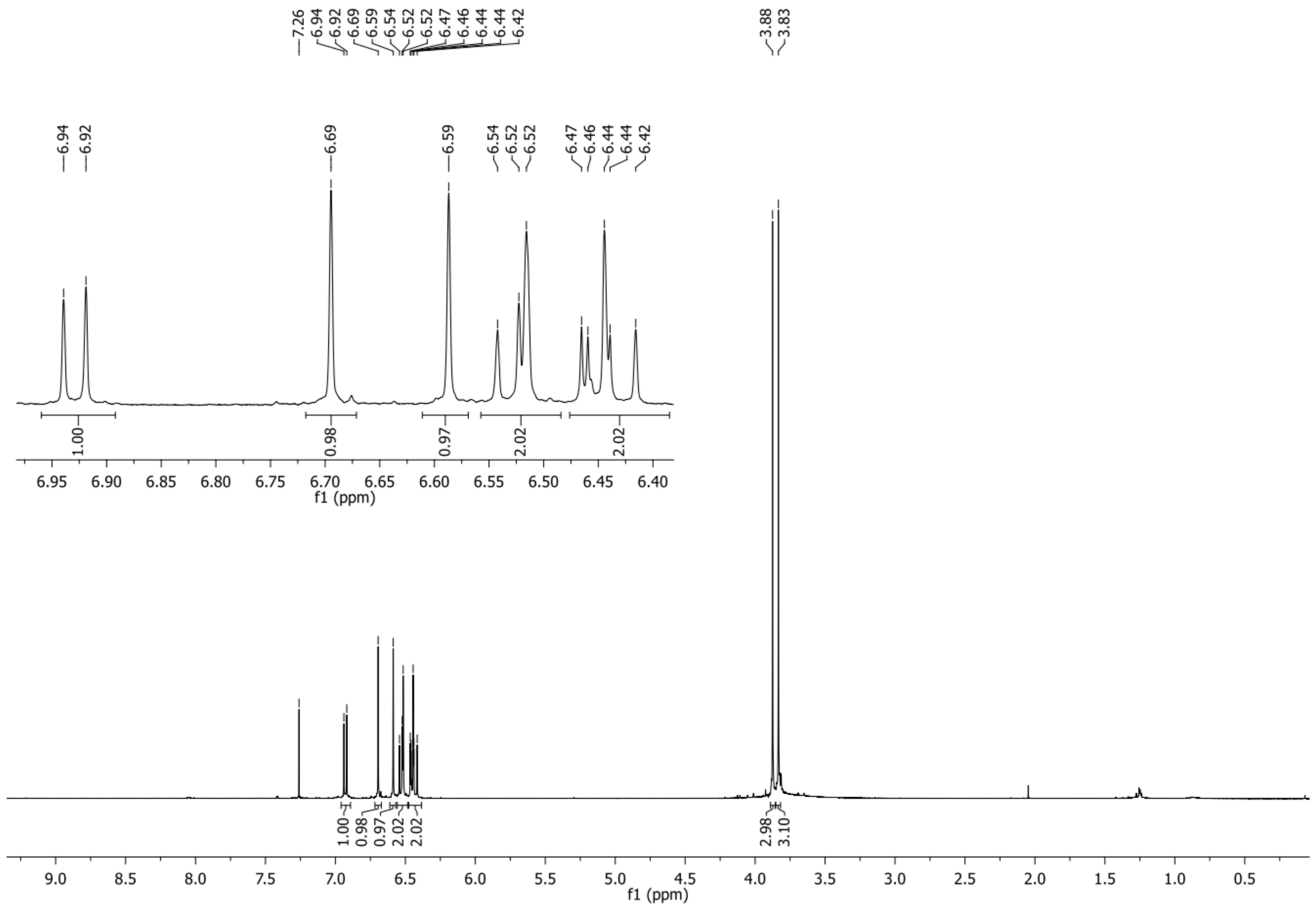

Figure S47. The ^{1}H NMR of 7,8-dimethoxydibenzo[*b,f*]oxepin-3-amine (**3f**).

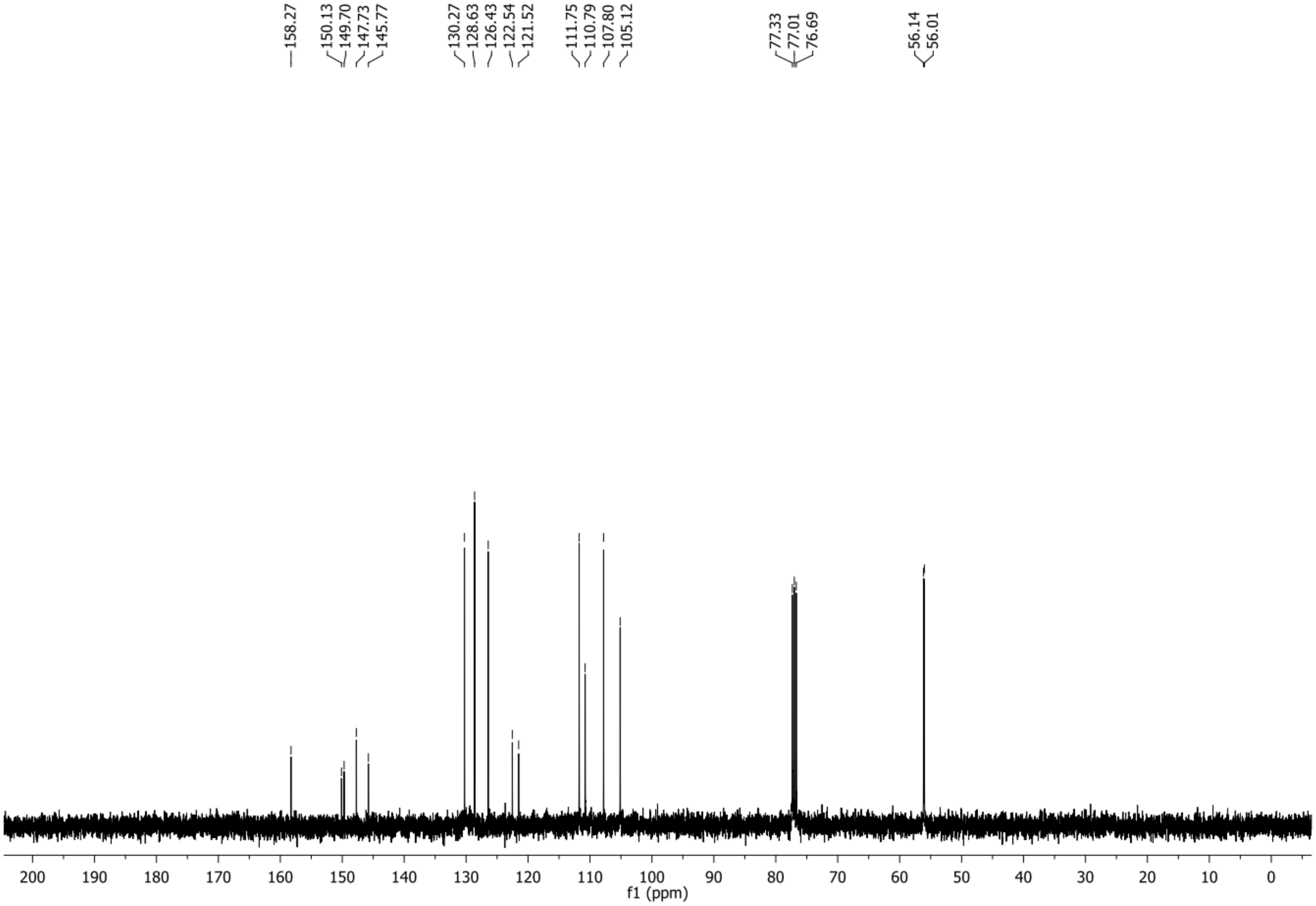

Figure S48. The ^{13}C NMR of 7,8-dimethoxydibenzo[*b*,*f*]oxepin-3-amine (**3f**).

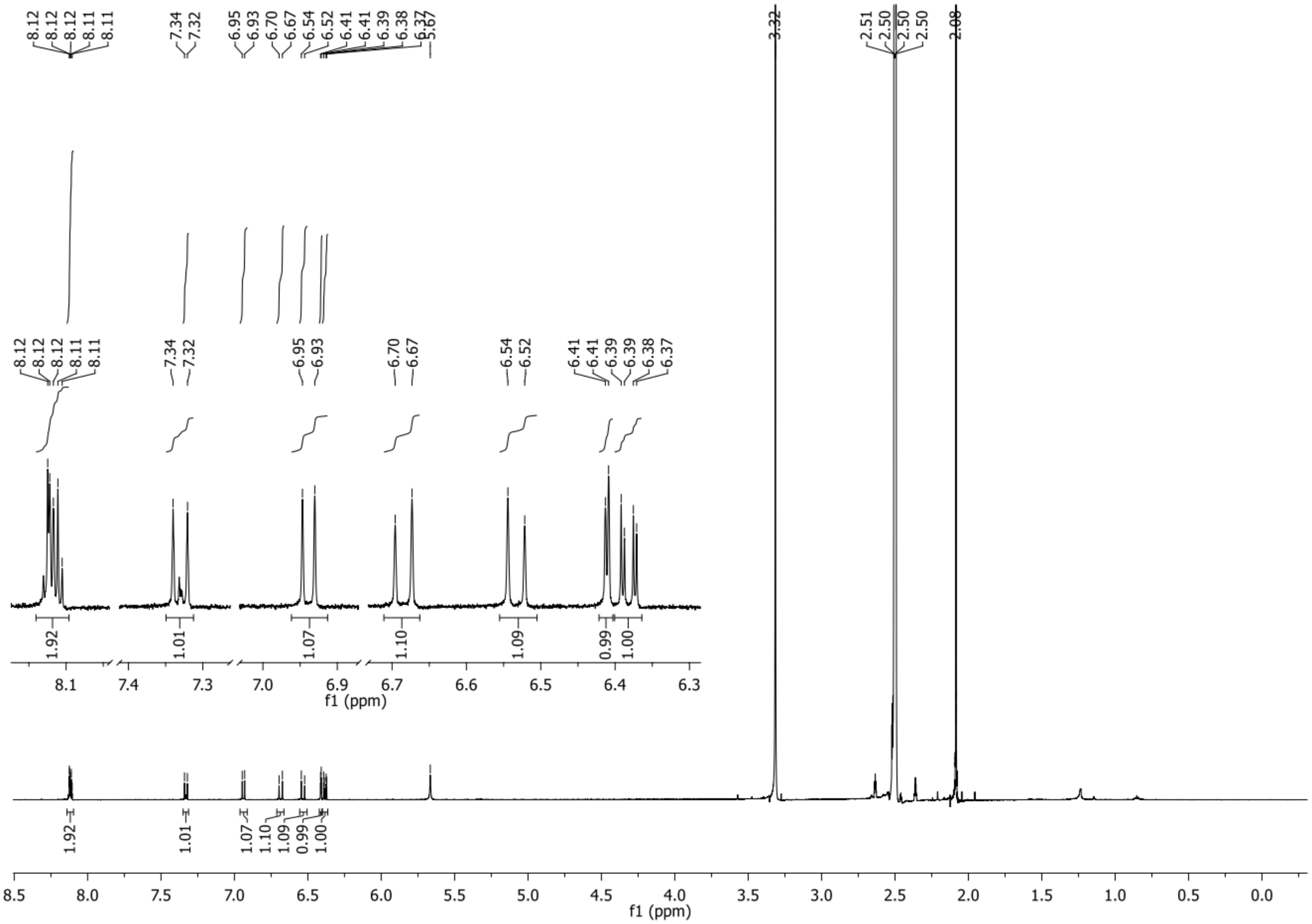

Figure S49. The ^{1}H NMR of 8-nitrodibenzo[*b*,*f*]oxepin-3-amine (**3g**).

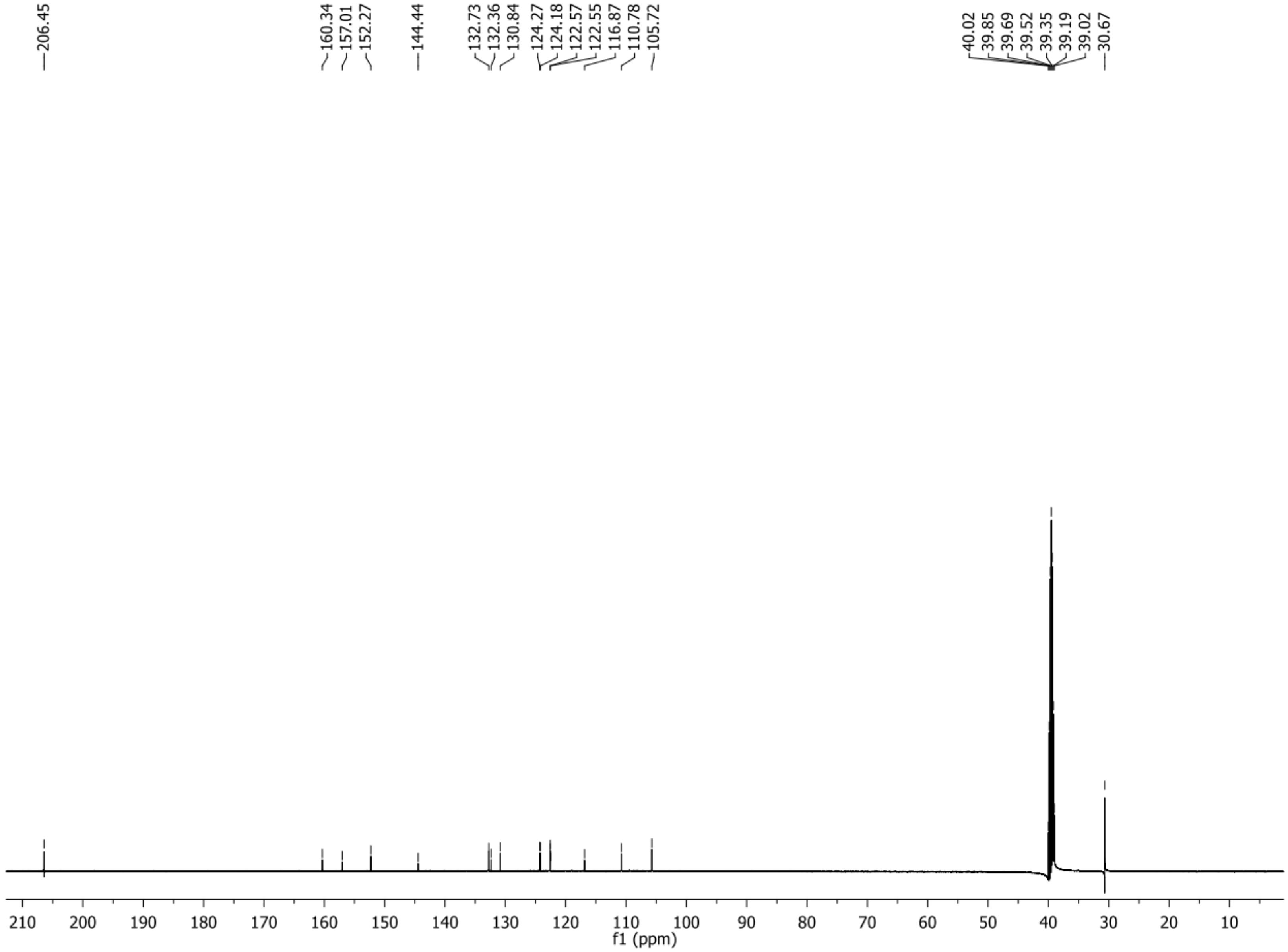

Figure S50. The ^{13}C NMR of 8-nitrodibenzo[*b,f*]oxepin-3-amine (**3g**).

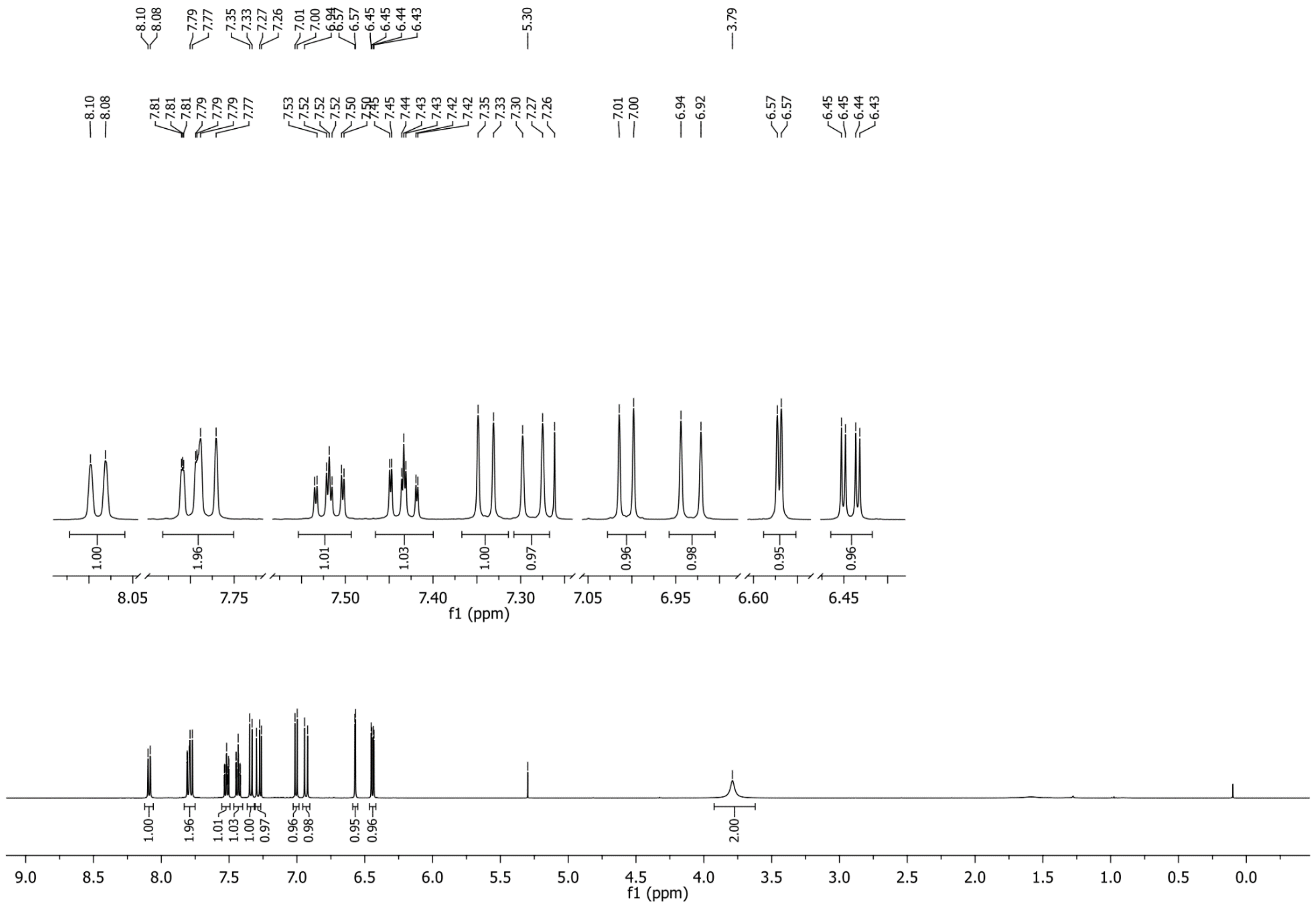

Figure S51. The ^{1}H NMR of benzo[*b*]naphtho[*1,2-f*]oxepin-9-amine (**3h**).

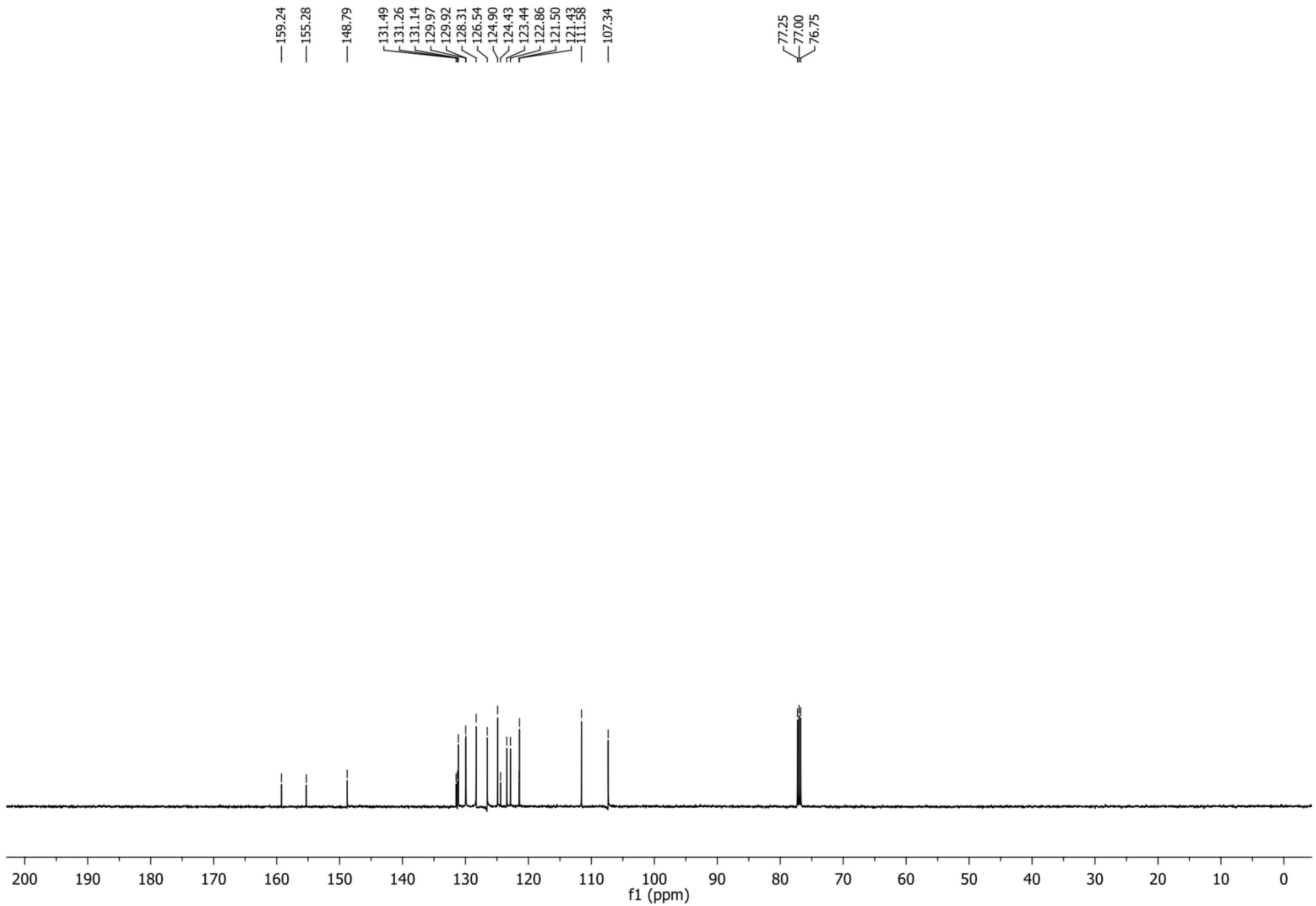

Figure S52. The ^{13}C NMR of benzo[*b*]naphtho[*1,2-f*]oxepin-9-amine (**3h**).

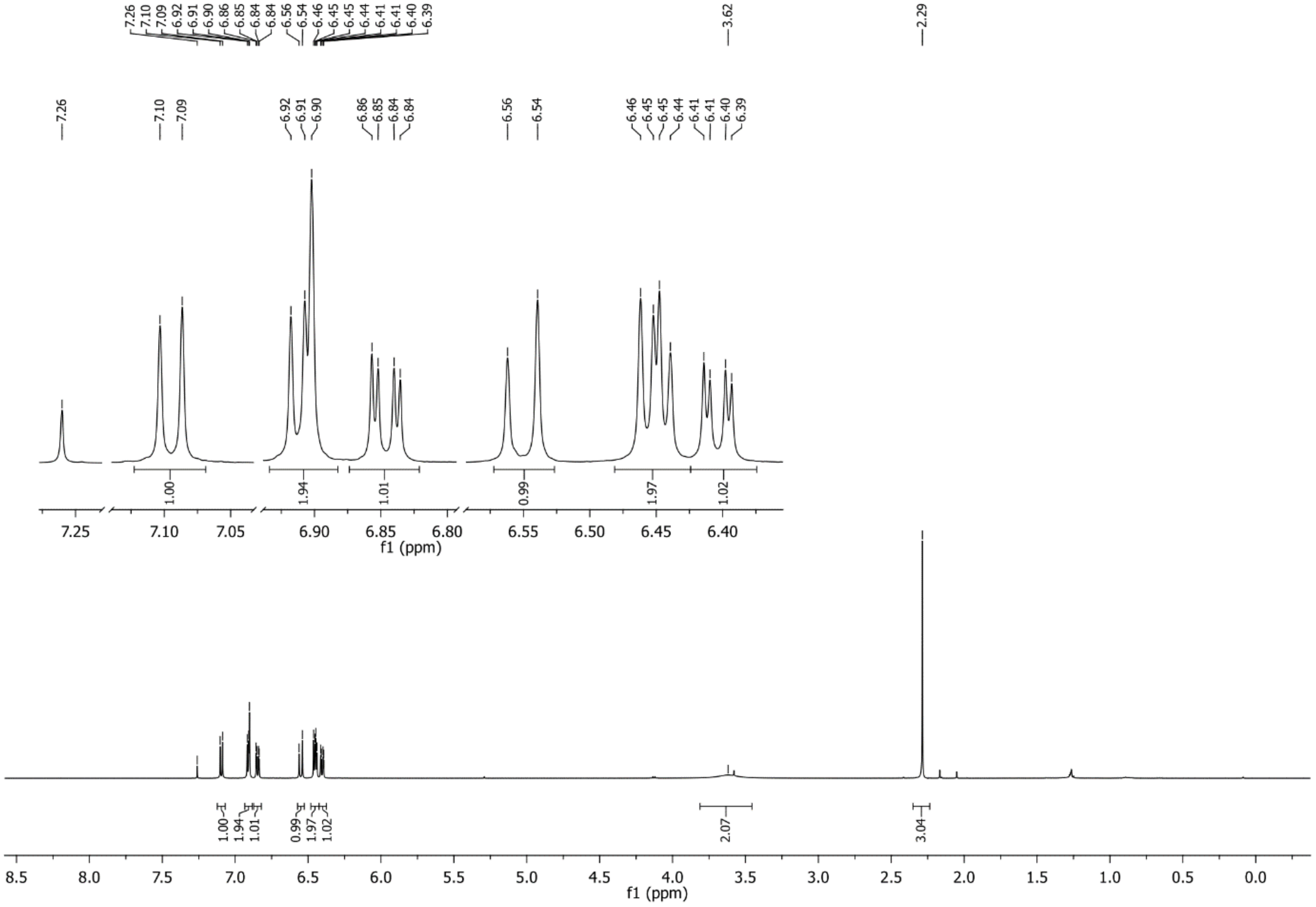

Figure S53. The ^{1}H NMR of 7-aminodibenzo[*b,f*]oxepin-3-yl acetate (**3j**).

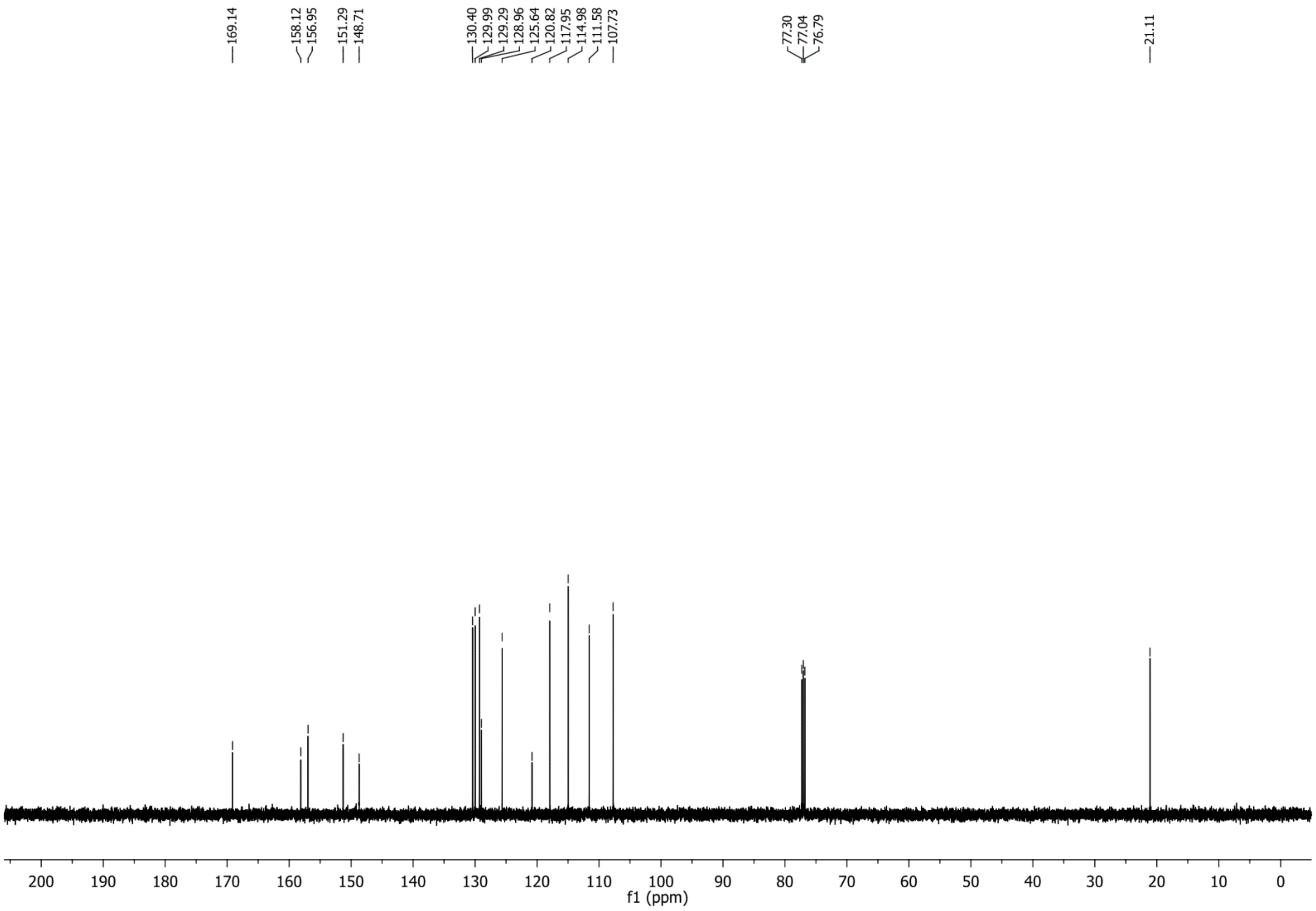

Figure S54. The ^{13}C NMR of 7-aminodibenzo[*b*,*f*]oxepin-3-yl acetate (**3j**).

Table S1-S7 The calculated coordinates of compounds (**1a**, **1c**, **1d**, **1i**, **2i**, **2j** and **3h**)
Figures S55-S61 Visualization of calculated geometry of compounds (**1a**, **1c**, **1d**, **1i**, **2i**, **2j** and **3h**)

Table S1. The calculated coordinates of (**1a**) (the part of calculated log file).

			Standard orientation		
			Coordinates(Angstroms)		
Center Number	Atomic Number	Atomic Type	X	Y	Z
1	6	0	0	5,246598	-1,82902
2	6	0	0	3,865267	-1,82381
3	6	0	0	3,128214	-0,67263
4	6	0	0	3,864096	0,50163
5	6	0	0	5,25579	0,497716
6	6	0	0	5,944337	-0,65624
7	6	0	0	1,68167	-0,79461
8	6	0	0	0,745744	0,152234
9	6	0	0	-0,68455	-0,12066
10	6	0	0	-1,18322	-1,434
11	6	0	0	-2,52524	-1,73711
12	6	0	0	-3,44284	-0,71324
13	6	0	0	-3,0242	0,59308
14	6	0	0	-1,67081	0,875698
15	8	0	0	3,182911	1,626543
16	7	0	0	-1,32961	2,299134
17	8	0	0	-0,43542	2,769427
18	8	0	0	-1,98668	2,947099
19	7	0	0	-4,86949	-1,01558
20	8	0	0	-5,65069	-0,08789
21	8	0	0	-5,21359	-2,18579
22	1	0	0	5,777135	-2,73394
23	1	0	0	3,316145	-2,73303
24	1	0	0	5,796012	1,412656
25	1	0	0	7,026776	-0,63766
26	1	0	0	1,345882	-1,80086
27	1	0	0	1,052376	1,166025
28	1	0	0	-0,48686	-2,23675
29	1	0	0	-2,87238	-2,75108
30	1	0	0	-3,73716	1,38195
31	1	0	0	3,798391	2,358281

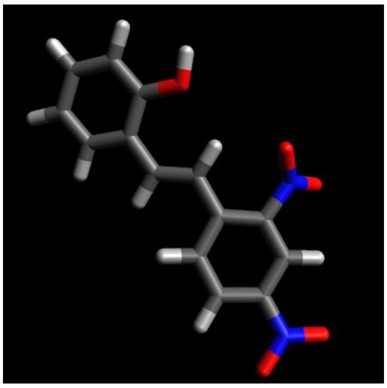

Figure S55. Visualization of calculated geometry of (**1a**).

Table S2. The calculated coordinates of (**1c**) (the part of calculated log file).

			Standard orientation		
			Coordinates(Angstroms)		
Center Number	Atomic Number	Atomic Type	X	Y	Z
1	6	0	4,600514	-1,46898	0,246864
2	6	0	3,220456	-1,53361	0,201936
3	6	0	2,396917	-0,41594	-0,02811
4	6	0	3,059785	0,824892	-0,22232
5	6	0	4,441237	0,907331	-0,17939
6	6	0	5,219598	-0,22859	0,054537
7	6	0	0,967962	-0,63005	-0,04484
8	6	0	-0,03782	0,267229	-0,20131
9	6	0	-1,44323	-0,0921	-0,17886
10	6	0	-1,86948	-1,43601	-0,31333
11	6	0	-3,18967	-1,81485	-0,24644
12	6	0	-4,16736	-0,84319	-0,0258
13	6	0	-3,8228	0,486917	0,118554
14	6	0	-2,48904	0,847825	0,017655
15	8	0	2,303119	1,927978	-0,45363
16	8	0	6,556959	-0,02578	0,07501
17	6	0	7,417783	-1,14305	0,309267
18	7	0	-2,23607	2,29062	0,152358
19	8	0	-1,3686	2,803203	-0,54696
20	8	0	-2,93545	2,914453	0,946277
21	7	0	-5,57016	-1,22579	0,045533
22	8	0	-6,40568	-0,34287	0,234047
23	8	0	-5,84796	-2,41687	-0,0849
24	1	0	5,173865	-2,3663	0,427497
25	1	0	2,741717	-2,49493	0,351866
26	1	0	4,936436	1,860943	-0,32867
27	1	0	0,706023	-1,67167	0,115769
28	1	0	0,202038	1,307736	-0,32143
29	1	0	-1,13063	-2,20269	-0,49845
30	1	0	-3,47707	-2,84937	-0,36966
31	1	0	-4,57943	1,235848	0,295914
32	1	0	2,866554	2,709406	-0,56709
33	1	0	8,428756	-0,74373	0,28596
34	1	0	7,223093	-1,5899	1,287029
35	1	0	7,303892	-1,89695	-0,47326

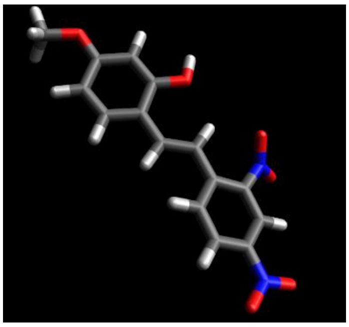

Figure S56. Visualization of calculated geometry of (**1c**).

Table S3. The calculated coordinates of (**1d**) (the part of calculated log file).

			Standard orientation		
			Coordinates(Angstroms)		
Center Number	Atomic Number	Atomic Type	X	Y	Z
1	6	0	4,513663	0,466444	-0,18315
2	6	0	3,128619	0,387569	-0,22262
3	6	0	2,447703	-0,80645	0,050849
4	6	0	3,214374	-1,94968	0,370534
5	6	0	4,6006	-1,86942	0,408702
6	6	0	5,255905	-0,67464	0,135971
7	6	0	0,998632	-0,91868	0,00963
8	6	0	0,123333	0,099683	-0,13607
9	6	0	-1,32126	-0,06589	-0,17278
10	6	0	-1,90178	-1,32919	-0,42902
11	6	0	-3,26176	-1,54192	-0,42939
12	6	0	-4,1153	-0,47369	-0,1573
13	6	0	-3,61542	0,786156	0,107564
14	6	0	-2,24358	0,981382	0,07656
15	8	0	2,548211	-3,11111	0,642291
16	7	0	-1,81649	2,364561	0,346667
17	8	0	-2,48396	3,01452	1,144599
18	8	0	-0,8411	2,806816	-0,25224
19	7	0	-5,56066	-0,67942	-0,1572
20	8	0	-6,28317	0,285219	0,081747
21	8	0	-5,97752	-1,81048	-0,3945
22	8	0	5,056556	1,687167	-0,47254
23	6	0	6,475082	1,816678	-0,44666
24	1	0	2,580117	1,282485	-0,48674
25	1	0	5,179987	-2,75304	0,656336
26	1	0	6,336025	-0,65069	0,173351
27	1	0	0,627309	-1,92773	0,135965
28	1	0	0,490468	1,111011	-0,20012
29	1	0	-1,25423	-2,16373	-0,65811
30	1	0	-3,67133	-2,51858	-0,64499
31	1	0	-4,2797	1,608359	0,325587
32	1	0	3,179351	-3,82544	0,817285
33	1	0	6,683284	2,853663	-0,70049
34	1	0	6,875828	1,599308	0,547608
35	1	0	6,947966	1,161225	-1,18376

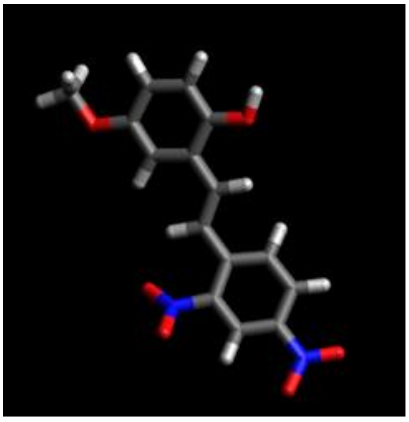

Figure S57. Visualization of calculated geometry of (**1d**).

Table S4. The calculated coordinates of (**1i**) (the part of calculated log file).

			Standard orientation		
			Coordinates(Angstroms)		
Center Number	Atomic Number	Atomic Type	X	Y	Z
1	6	0	4,918542	-1,72147	0,328398
2	6	0	3,540586	-1,73956	0,274652
3	6	0	2,763824	-0,58991	0,023282
4	6	0	3,47529	0,620502	-0,18056
5	6	0	4,861874	0,652988	-0,12884
6	6	0	5,584887	-0,50898	0,124676
7	6	0	1,327758	-0,7469	-0,00423
8	6	0	0,359681	0,18782	-0,18
9	6	0	-1,0589	-0,11498	-0,16794
10	6	0	-1,5366	-1,44264	-0,29116
11	6	0	-2,87119	-1,76862	-0,23362
12	6	0	-3,8122	-0,75681	-0,03479
13	6	0	-3,41696	0,560267	0,097519
14	6	0	-2,069	0,867646	0,006582
15	8	0	2,764847	1,748544	-0,43108
16	8	0	6,936312	-0,39973	0,159403
17	7	0	-1,76156	2,300991	0,127086
18	8	0	-0,86451	2,770348	-0,5653
19	8	0	-2,44732	2,962136	0,902574
20	7	0	-5,22949	-1,08327	0,02654
21	8	0	-6,03169	-0,16606	0,19521
22	8	0	-5,55249	-2,2642	-0,0919
23	1	0	5,47984	-2,62826	0,523856
24	1	0	3,023118	-2,67922	0,432412
25	1	0	5,393003	1,585591	-0,28675
26	1	0	1,023046	-1,77545	0,164984
27	1	0	0,641727	1,216562	-0,30874
28	1	0	-0,82687	-2,24006	-0,45948
29	1	0	-3,19788	-2,79251	-0,34746
30	1	0	-4,14517	1,340535	0,257965
31	1	0	3,358632	2,506454	-0,54976
32	1	0	7,341153	-1,26227	0,342526
1	6	0	4,918542	-1,72147	0,328398
2	6	0	3,540586	-1,73956	0,274652
3	6	0	2,763824	-0,58991	0,023282

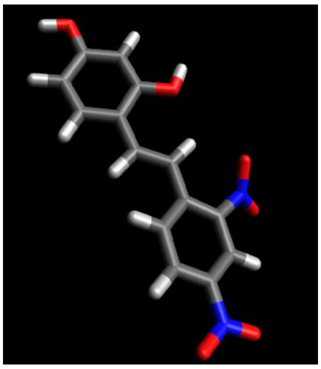

Figure S58. Visualization of calculated geometry of (**1i**).

Table S5. The calculated coordinates of (**2i**) (the part of calculated log file).

			Standard orientation		
			Coordinates(Angstroms)		
Center Number	Atomic Number	Atomic Type	X	Y	Z
1	6	0	3,636264	-1,36171	-0,23615
2	6	0	4,230331	-0,17388	-0,66455
3	6	0	3,547301	1,018694	-0,51443
4	6	0	2,26066	1,082145	0,047113
5	6	0	1,702678	-0,12915	0,480094
6	6	0	2,367158	-1,33568	0,343113
7	6	0	1,583376	2,362991	0,157186
8	6	0	0,258433	2,601504	0,223615
9	6	0	-0,80855	1,616337	0,158563
10	6	0	-0,64214	0,271827	0,541672
11	8	0	0,5058	-0,15631	1,187558
12	6	0	-2,07345	1,999872	-0,32042
13	6	0	-3,1147	1,099215	-0,44524
14	6	0	-2,89392	-0,22395	-0,07914
15	6	0	-1,6651	-0,64873	0,415449
16	8	0	4,343103	-2,51255	-0,39094
17	7	0	-3,97942	-1,19209	-0,19823
18	8	0	-5,05898	-0,80163	-0,64049
19	8	0	-3,76371	-2,35339	0,146182
20	1	0	5,220629	-0,20139	-1,10142
21	1	0	4,013203	1,940199	-0,84507
22	1	0	1,902769	-2,2433	0,712209
23	1	0	2,234087	3,230716	0,10222
24	1	0	-0,06486	3,637016	0,216316
25	1	0	-2,22912	3,033906	-0,60431
26	1	0	-4,08164	1,405717	-0,81643
27	1	0	-1,51582	-1,67275	0,725493
28	1	0	3,836853	-3,2714	-0,06115

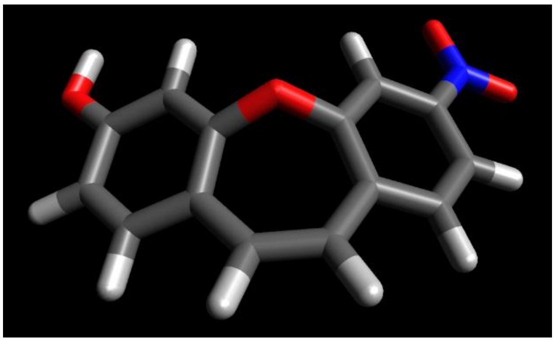

Figure S59. Visualization of calculated geometry of (**2i**).

Table S6. The calculated coordinates of (**2j**) (the part of calculated log file).

			Standard orientation		
			Coordinates(Angstroms)		
Center Number	Atomic Number	Atomic Type	X	Y	Z
1	6	0	2,947276	-0,13861	-0,55254
2	6	0	3,212441	1,164023	-0,95086
3	6	0	2,271601	2,147972	-0,68791
4	6	0	1,056761	1,860989	-0,04834
5	6	0	0,832914	0,531506	0,338406
6	6	0	1,759268	-0,46765	0,085751
7	6	0	0,09412	2,931653	0,181235
8	6	0	-1,2413	2,824814	0,304007
9	6	0	-2,03528	1,607108	0,215513
10	6	0	-1,52613	0,333679	0,526372
11	8	0	-0,2738	0,181874	1,09968
12	6	0	-3,3735	1,685733	-0,20366
13	6	0	-4,16568	0,559687	-0,33652
14	6	0	-3,61069	-0,67883	-0,03761
15	6	0	-2,29575	-0,80684	0,395095
16	8	0	3,849061	-1,15336	-0,86713
17	6	0	4,867614	-1,51977	-0,02313
18	8	0	5,595774	-2,40396	-0,38674
19	6	0	4,989975	-0,78985	1,285485
20	7	0	-4,43174	-1,88274	-0,16427
21	8	0	-5,59066	-1,75383	-0,55263
22	8	0	-3,92229	-2,96433	0,121041
23	1	0	4,140522	1,399233	-1,45705
24	1	0	2,472676	3,169143	-0,99044
25	1	0	1,558865	-1,48417	0,400397
26	1	0	0,510415	3,934113	0,169274
27	1	0	-1,81152	3,744675	0,380964
28	1	0	-3,79103	2,65902	-0,43241
29	1	0	-5,19335	0,630337	-0,66144
30	1	0	-1,88542	-1,7731	0,65027
31	1	0	5,128607	0,28016	1,120537
32	1	0	4,083817	-0,91282	1,881725
33	1	0	5,84213	-1,19225	1,826455

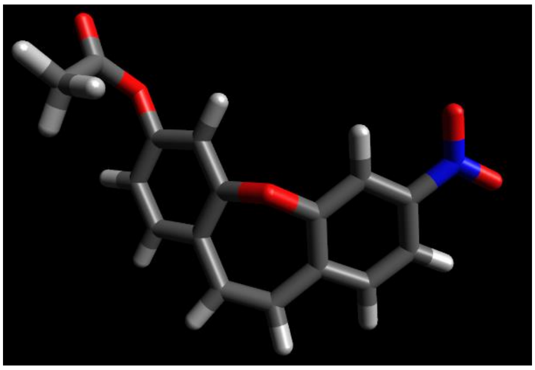

Figure S60. Visualization of calculated geometry of (**2j**).

Table S7. The calculated coordinates of (**3h**) (the part of calculated log file).

			Standard orientation		
			Coordinates(Angstroms)		
Center Number	Atomic Number	Atomic Type	X	Y	Z
1	6	0	-2,15002	-2,14578	0,085908
2	6	0	-2,93467	-0,99044	-0,14732
3	6	0	-2,35916	0,302878	0,059475
4	6	0	-1,00014	0,414836	0,525499
5	6	0	-0,29254	-0,75878	0,726912
6	6	0	-0,85245	-2,02978	0,504715
7	6	0	-0,40182	1,724661	0,775946
8	6	0	0,895666	2,060169	0,636816
9	6	0	1,993861	1,191641	0,244273
10	6	0	1,994097	-0,18597	0,503707
11	8	0	0,985223	-0,75012	1,279124
12	6	0	3,129993	1,705759	-0,40015
13	6	0	4,18287	0,900053	-0,79327
14	6	0	4,151747	-0,48063	-0,5407
15	6	0	3,036076	-1,01106	0,122826
16	7	0	5,176832	-1,30852	-0,97242
17	6	0	-4,2774	-1,09692	-0,58818
18	6	0	-5,03401	0,020738	-0,82926
19	6	0	-4,46764	1,29852	-0,64501
20	6	0	-3,16884	1,434974	-0,2183
21	1	0	-2,59051	-3,12415	-0,0695
22	1	0	-0,24033	-2,90223	0,697537
23	1	0	-1,08406	2,524114	1,037299
24	1	0	1,15442	3,10625	0,774897
25	1	0	3,177289	2,771236	-0,59832
26	1	0	5,041644	1,333591	-1,29345
27	1	0	2,988818	-2,06561	0,369026
28	1	0	6,07256	-0,86521	-1,12827
29	1	0	5,262407	-2,19899	-0,50026
30	1	0	-4,6963	-2,08653	-0,73548
31	1	0	-6,05935	-0,0734	-1,16686
32	1	0	-5,06036	2,182791	-0,84886
33	1	0	-2,75968	2,429537	-0,10716

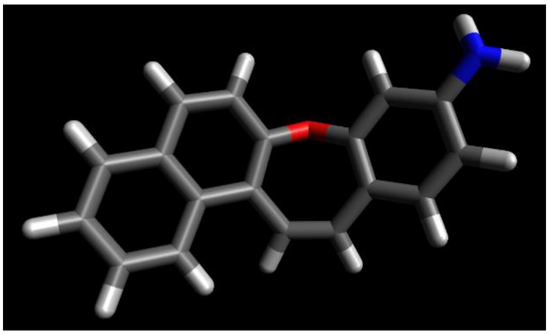

Figure S61. Visualization of calculated geometry of (**3h**).

Table S8. Estimated binding free energy, visualization of binding pose and predicted interactions with αβ-tubulin heterodimer for compounds (**1a**, **1c**, **1d**, **1i**, **2i**, **2j, 3h**). For comparison native ligand structure is superimposed as yellow sticks.

Compound	Binding pose and interactions	Binding pose and interactions superimposed with DAMA-colchicine	Estimated binding free energy (kJ/mol)	Type of interaction	Active residues
**1a**	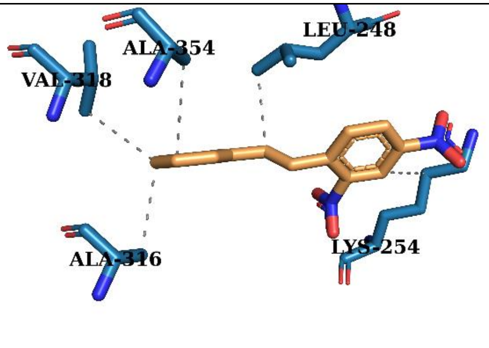	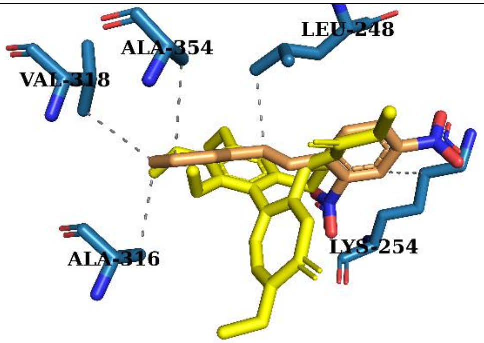	-32.6	Hydrophobic Interactions	LEU248<br>LYS254<br>ALA316<br>VAL318<br>ALA354
**1c**	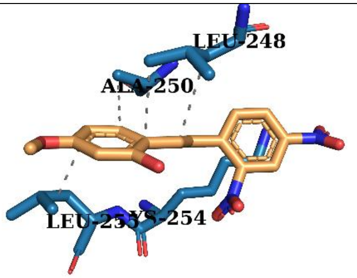	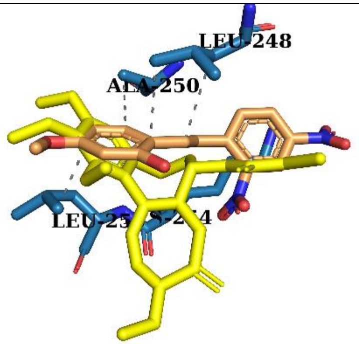	-32.2	Hydrophobic Interactions	LEU248<br>ALA250<br>LYS254<br>LEU255

<table>
<tr><td rowspan="2">1d</td><td rowspan="2">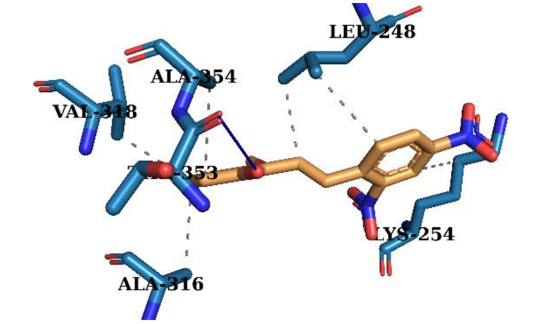</td><td rowspan="2">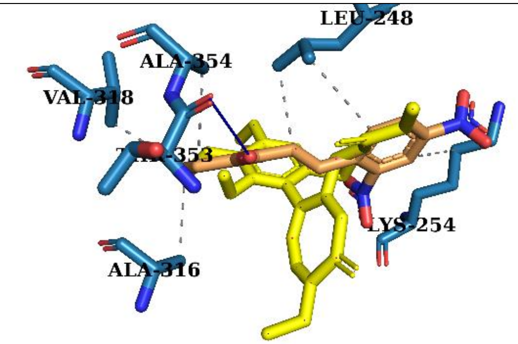</td><td rowspan="2">-32.6</td><td>Hydrophobic Interactions</td><td>LEU248
LYS254
ALA316
VAL318
ALA354</td></tr>
<tr><td>Hydrogen Bonds</td><td>THR353</td></tr>
<tr><td rowspan="2">1i</td><td rowspan="2">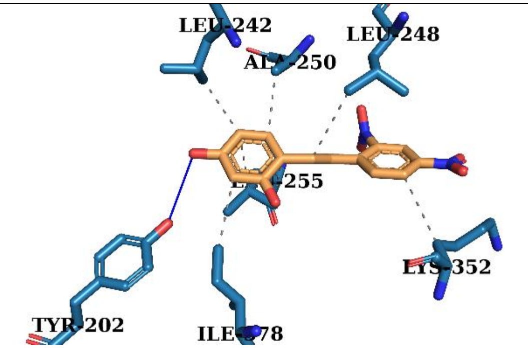</td><td rowspan="2">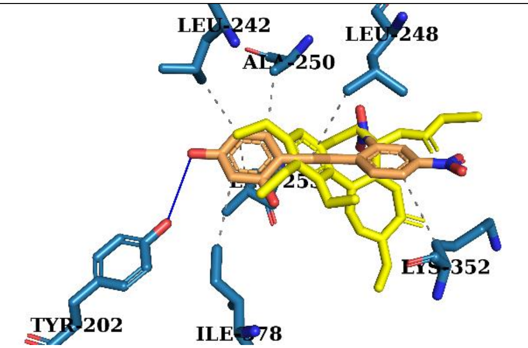</td><td rowspan="2">-32.6</td><td>Hydrophobic Interactions</td><td>LEU242
LEU248
ALA250
LEU255
LYS352
ILE378</td></tr>
<tr><td>Hydrogen Bonds</td><td>TYR202</td></tr>
<tr><td rowspan="2">2i</td><td rowspan="2">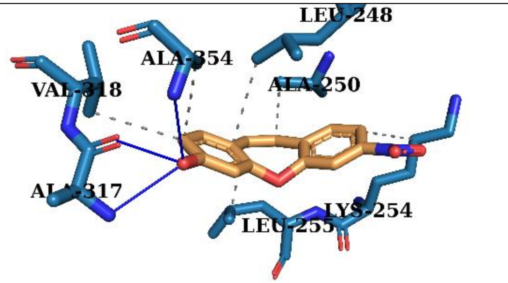</td><td rowspan="2">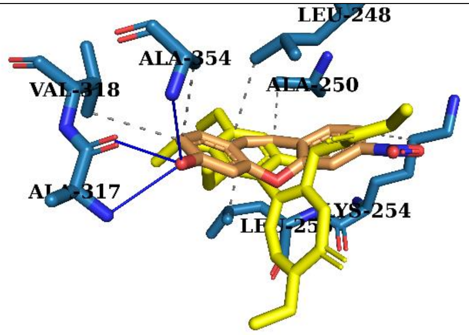</td><td rowspan="2">-33.9</td><td>Hydrophobic Interactions</td><td>LEU248
ALA250
LYS254
LEU255
VAL318
ALA354</td></tr>
<tr><td>Hydrogen Bonds</td><td>ALA316
ALA354</td></tr>
</table>

**2j**	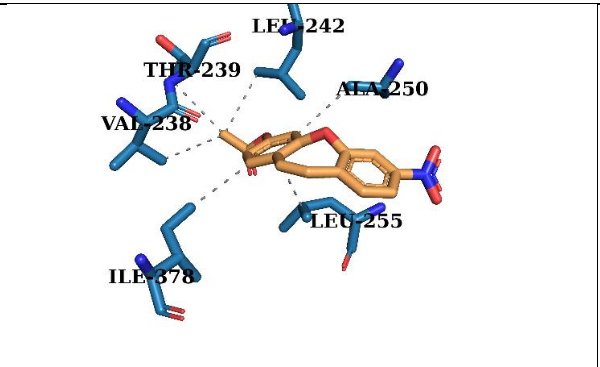	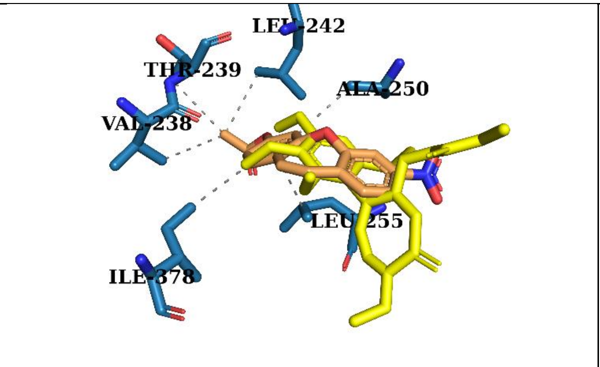	-36.0	Hydrophobic Interactions	VAL238<br>THR239<br>LEU242<br>ALA250<br>LEU255<br>ILE378
**3h**	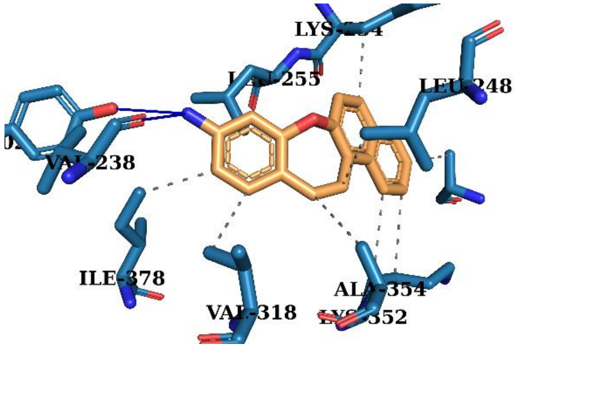	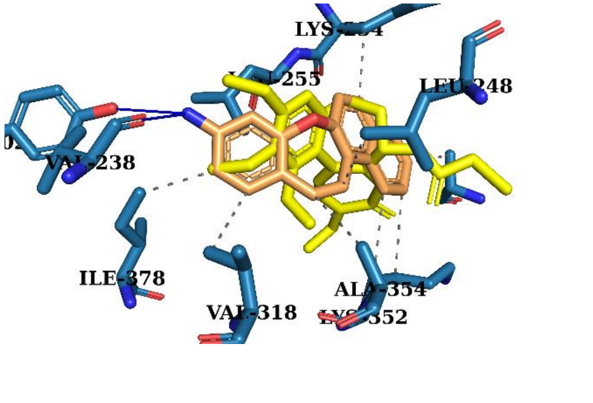	-38.9	Hydrophobic Interactions	ALA180<br>LEU248<br>LYS254<br>LEU255<br>VAL318<br>LYS352<br>ALA354<br>ILE378
				Hydrogen Bonds	TYR202<br>VAL238
